# Hearing impairment due to *Mir183/96/182* mutations suggests both loss-of-function and gain-of-function effects

**DOI:** 10.1242/dmm.047225

**Published:** 2021-02-15

**Authors:** Morag A. Lewis, Francesca Di Domenico, Neil J. Ingham, Haydn M. Prosser, Karen P. Steel

**Affiliations:** 1 Wolfson Centre for Age-Related Diseases, King's College London, London, SE1 1UL, UK; 2 Wellcome Trust Sanger Institute, Hinxton, Cambridge CB10 1SA, UK

**Keywords:** MicroRNAs, Hearing loss, miR-96, Networks, miR-183, miR-182

## Abstract

The microRNA miR-96 is important for hearing, as point mutations in humans and mice result in dominant progressive hearing loss. *Mir96* is expressed in sensory cells along with *Mir182* and *Mir183*, but the roles of these closely-linked microRNAs are as yet unknown. Here, we analyse mice carrying null alleles of *Mir182*, and of *Mir183* and *Mir96* together to investigate their roles in hearing. We found that *Mir183*/*96* heterozygous mice had normal hearing and homozygotes were completely deaf with abnormal hair cell stereocilia bundles and reduced numbers of inner hair cell synapses at 4 weeks of age. *Mir182* knockout mice developed normal hearing then exhibited progressive hearing loss. Our transcriptional analyses revealed significant changes in a range of other genes, but surprisingly there were fewer genes with altered expression in the organ of Corti of *Mir183/96* null mice compared with our previous findings in *Mir96^Dmdo^* mutants, which have a point mutation in the miR-96 seed region. This suggests that the more-severe phenotype of *Mir96^Dmdo^* mutants compared with *Mir183*/*96* mutants, including progressive hearing loss in *Mir96^Dmdo^* heterozygotes, is likely to be mediated by the gain of novel target genes in addition to the loss of its normal targets. We propose three mechanisms of action of mutant miRNAs: loss of targets that are normally completely repressed, loss of targets for which transcription is normally buffered by the miRNA, and gain of novel targets. Any of these mechanisms could lead to a partial loss of a robust cellular identity and consequent dysfunction.

## Abstract (precis)

**Summary:** Our study describes mice carrying knockout alleles of microRNAs involved in hearing and suggests that a point mutation in a microRNA can have a greater phenotypic impact than a null allele.

## INTRODUCTION

The microRNAs (miRNAs) miR-96, miR-182 and miR-183 are expressed together on a single transcript in sensory cells, including the retina and the hair cells of the inner ear (Weston et al., 2006; Xu et al., 2007). Point mutations in *Mir96* cause rapidly progressive hearing loss in the diminuendo mouse mutant [*Mir96^Dmdo^* (Lewis et al., 2009)] and progressive hearing loss with later onset in human families (Mencia et al., 2009; Solda et al., 2012), and the diminuendo mutation has also been shown to delay maturation of the central auditory system (Schluter et al., 2018). In homozygous *Mir96^Dmdo^* mice, most of the cochlear hair cells die by 28 days after birth. However, this is not the cause of the hearing loss; even before the onset of normal hearing, homozygote hair cells fail to mature both morphologically and physiologically, remaining in their immature state, and heterozygote hair cells show a developmental delay. miR-96 is thus thought to be responsible for coordinating hair cell maturation (Chen et al., 2014; Kuhn et al., 2011). Overexpression of the three miRNAs also results in cochlear defects and hearing loss (Weston et al., 2018). The complete loss of all mature miRNAs from the inner ear results in early developmental defects including a severely truncated cochlear duct (Friedman et al., 2009; Soukup et al., 2009). miR-96, miR-182 and miR-183 have also been implicated in other diseases, including glaucoma (Liu et al., 2016), ischemic injury (Cui and Yang, 2013; Duan et al., 2019) and spinal cord injury (Ling et al., 2017).

MicroRNAs regulate the expression of many other genes by targeting specific sequences in their mRNAs, leading to transcript destabilisation or translational inhibition. Transcriptome analyses of the *Mir96^Dmdo^* organ of Corti showed that many genes were misregulated in homozygotes, including several known to be important for hearing that appear to contribute to specific aspects of the diminuendo phenotype (Chen et al., 2014; Kuhn et al., 2011; Lewis et al., 2016, 2009). However, the diminuendo mutation is a single base pair change in the seed region of the miRNA that is critical for correct targeting, and it is not clear to what extent the diminuendo mutant phenotype is the result of the loss of normal targets of miR-96, and how much is due to the gain of novel targets. We previously suggested that the progressive hearing loss was most likely caused by the loss of normal target repression because all three point mutations in mouse and human *Mir96* lead to a similar phenotype, which seems unlikely if the gain of novel targets is the main mechanism involved.

The regulatory network generated from *Mir96^Dmdo^* expression data (Lewis et al., 2016) includes a number of genes known to be involved in deafness – such as *Ptprq*, *Gfi1*, *Kcna10* and *Slc26a5* – as well as new candidate genes. Manipulating this network could be a useful therapeutic approach to treating hearing loss due to hair cell dysfunction triggered by a broad range of factors, including genetic variants and environmental insults. For example, *Trp53*, *Hif1a* and *Nfe2l2* are in the *Mir96^Dmdo^* network and are involved in cellular responses to stress (Simmons et al., 2009). In order to focus our translational efforts, it is important to understand better the molecular basis of the network. For this reason, we have analysed a second mutation of *Mir96* in this study, a double knockout of *Mir96* and *Mir183*, as well as a knockout of the closely linked *Mir182* gene, generated through a mouse miRNA knockout programme (Prosser et al., 2011). Although both new mouse mutants exhibit hearing loss, their phenotypes differ from the diminuendo mouse, with no sign of hearing loss in the heterozygotes, suggesting that the more-severe phenotype of *Mir96^Dmdo^* mutants is likely to be mediated by the gain of novel target genes in addition to the loss of its normal targets.

## RESULTS

### *Mir183*/*96* and *Mir182* knockout mice

**Fig. 1.**
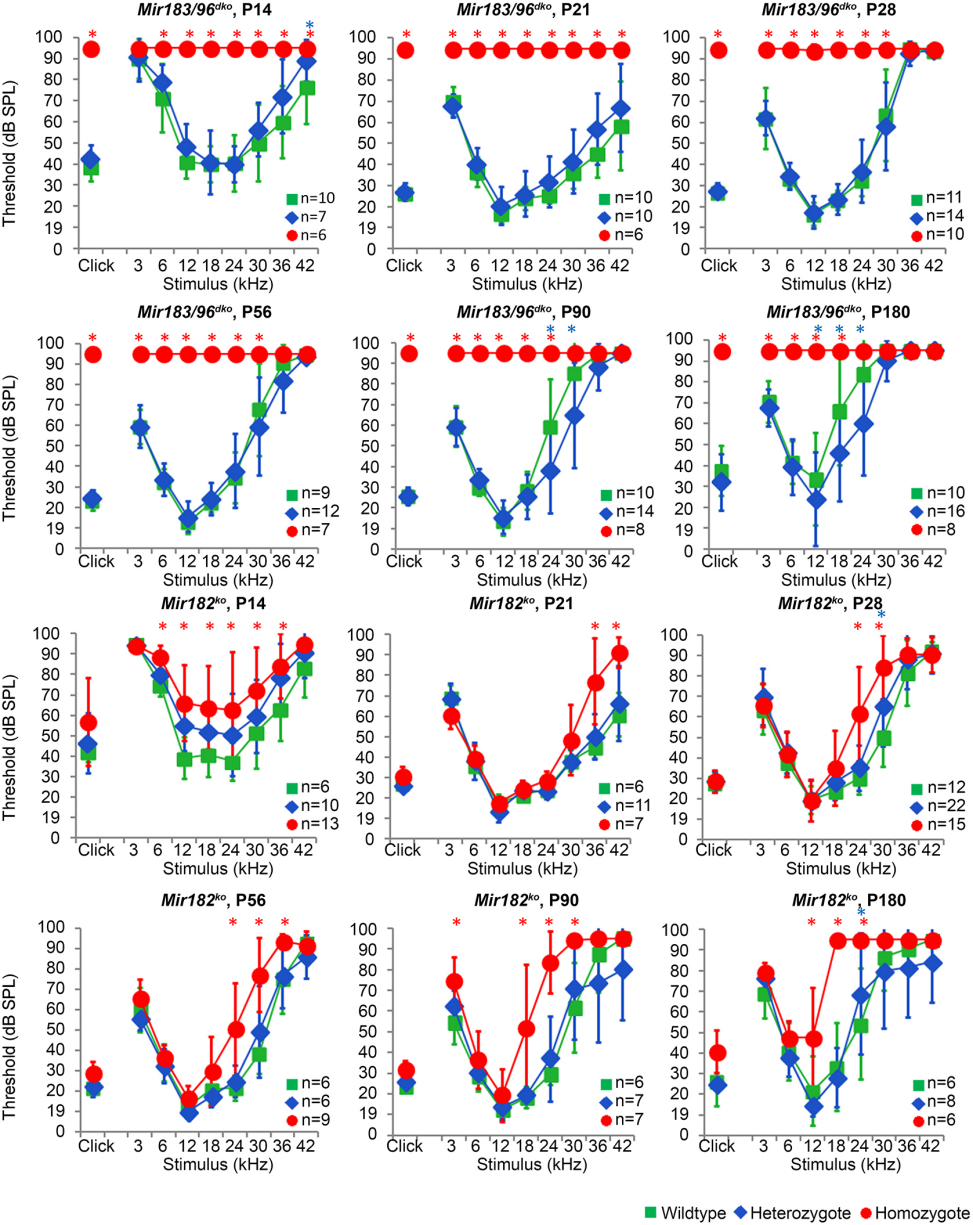
**Mean** **auditory brainstem response** **(ABR) thresholds of *Mir183/96^dko^* and *Mir182^ko^* homozygous, heterozygous and wild-type mice tested at P14, P21, P28, P56, P90 and P180.** Homozygous *Mir183/96^dko^* mice show profound hearing loss at all ages tested (points plotted at 95 dB indicate no response at this level, the maximum SPL used). *Mir182^ko^* homozygotes (red circles) display mildly raised thresholds at high frequencies, which slowly progresses to include the middle frequencies as the mice age. Heterozygotes from both lines (blue diamonds) show thresholds similar to those of wild-type mice (green squares). Numbers of each genotype tested at each age are shown on the threshold plot. Error bars are s.d. (Bonferroni-corrected *P<0.05, mixed linear model pairwise comparison; asterisk is marked in red for a significant difference between wild type and homozygote or blue for a significant difference between wild type and heterozygote). See Figs S2 and S3 for individually plotted traces. See Data S1 for the data and statistical analyses underlying these graphs.

Two mouse lines were used in this study; a knockout of *Mir182* (*Mir182^tm1Hmpr/Wtsi^*, hereafter referred to as *Mir182^ko^*) and a double knockout of both *Mir183* and *Mir96* (*Mirc40^tm1Hmpr/WtsiOulu^*, hereafter referred to as *Mir183/96^dko^*), which are only 116 bp apart, making it technically challenging to generate two separate knockouts. The mice were generated and maintained on the C57BL/6N genetic background. C57BL/6 mice are known to have age-related hearing loss, partly due to the *Cdh23^ahl^* allele (Noben-Trauth et al., 2003). Higher frequencies are affected first, after 4 weeks of age, whereas the lower frequencies remain unaffected up to 6 months of age (Li and Borg, 1991). We observed a similar pattern in wild-type mice from both the *Mir183*/*96^dko^* and *Mir182^ko^* lines, which exhibited mild progressive hearing loss at 24-42 kHz from 8 weeks of age but retained good hearing sensitivity at frequencies between 3-12 kHz up to 6 months of age (Fig. 1).

For the *Mir183*/*96^dko^* mice, 43 out of 242 mice (17.8%) from heterozygote by heterozygote matings were homozygous for the *Mir183*/*96* null allele, which is lower than expected (25%), suggesting that the absence of *Mir96* and/or *Mir183* has a small impact on viability (*P*=0.029, chi-squared test). For the *Mir182^ko^* mice, 42 homozygotes out of 152 pups in total (27.6%) were produced from heterozygote by heterozygote matings, which is consistent with the mutation having no impact on viability.

### Complete knockout of miRNA expression in the mutant organ of Corti

We carried out quantitative PCR (qPCR) to test the expression levels of the three miRNAs in the organs of Corti of wild types, heterozygotes and homozygotes of each knockout at postnatal day (P)4. In *Mir183/96^dko^* homozygotes, there was no detectable expression of *Mir183* or *Mir96*. Likewise, in *Mir182^ko^* homozygotes, there was no detectable expression of *Mir182* (Fig. S1). The levels of expression in heterozygotes of both knockouts was variable, as was the expression of *Mir182* in *Mir183/96^dko^* homozygotes, and *Mir183* and *Mir96* in *Mir182^ko^* homozygotes. It is likely that this is because we lack a proper miRNA control for sensory tissue in the inner ear. We used *Mir99a*, which is expressed in almost all cell types in the cochlea, including hair and supporting cells (Friedman et al., 2009), but because *Mir183*, *Mir182* and *Mir96* are also expressed in hair cells, it is possible that the mutant alleles affect the expression of *Mir99a* in hair cells, making it an unreliable calibrator between wild type, heterozygote and homozygote. A better calibrator would be an miRNA expressed only in supporting cells and not in hair cells.

### Impaired auditory responses in homozygotes but normal thresholds in heterozygotes

*Mir183/96^dko^* homozygous mice were profoundly deaf, with most showing no response at the highest sound level tested [95 dB sound pressure level (SPL)] at any of the ages tested (14 days to 6 months old). By contrast, *Mir182^ko^* homozygotes exhibited only a mild hearing loss starting at higher frequencies and progressing with age to lower frequencies (Fig. 1). The auditory brainstem response (ABR) thresholds of heterozygotes were normal at all ages tested (Fig. 1; see Figs S2 and S3 for individually plotted traces). ABR waveforms of *Mir183/96^dko^* heterozygotes and *Mir182^ko^* homozygotes were similar to those of wild-type littermates at the equivalent sound pressure level above threshold [sensation level (SL)] (Fig. S4). We measured distortion product otoacoustic emissions (DPOAEs) at 8 weeks of age and found no difference in the amplitudes or thresholds between wild-type and heterozygous *Mir183/96^dko^* mice, whereas homozygotes had severely abnormal responses (Fig. S6). *Mir182^ko^* homozygotes had raised DPOAE thresholds at high frequencies compared with wild types (Fig. S6), which matched the difference in their ABR thresholds at 8 weeks (Fig. 1). *Mir182^ko^* mutant mice showed no sign of a vestibular defect (circling, head-bobbing or hyperactivity) up to 6 months of age. However, *Mir183/96^dko^* homozygotes did show increasing incidence of hyperactivity with age (Fig. S5).

### Heterozygous *Mir183/96^dko^* mice recover normally from noise exposure

**Fig. 2.**
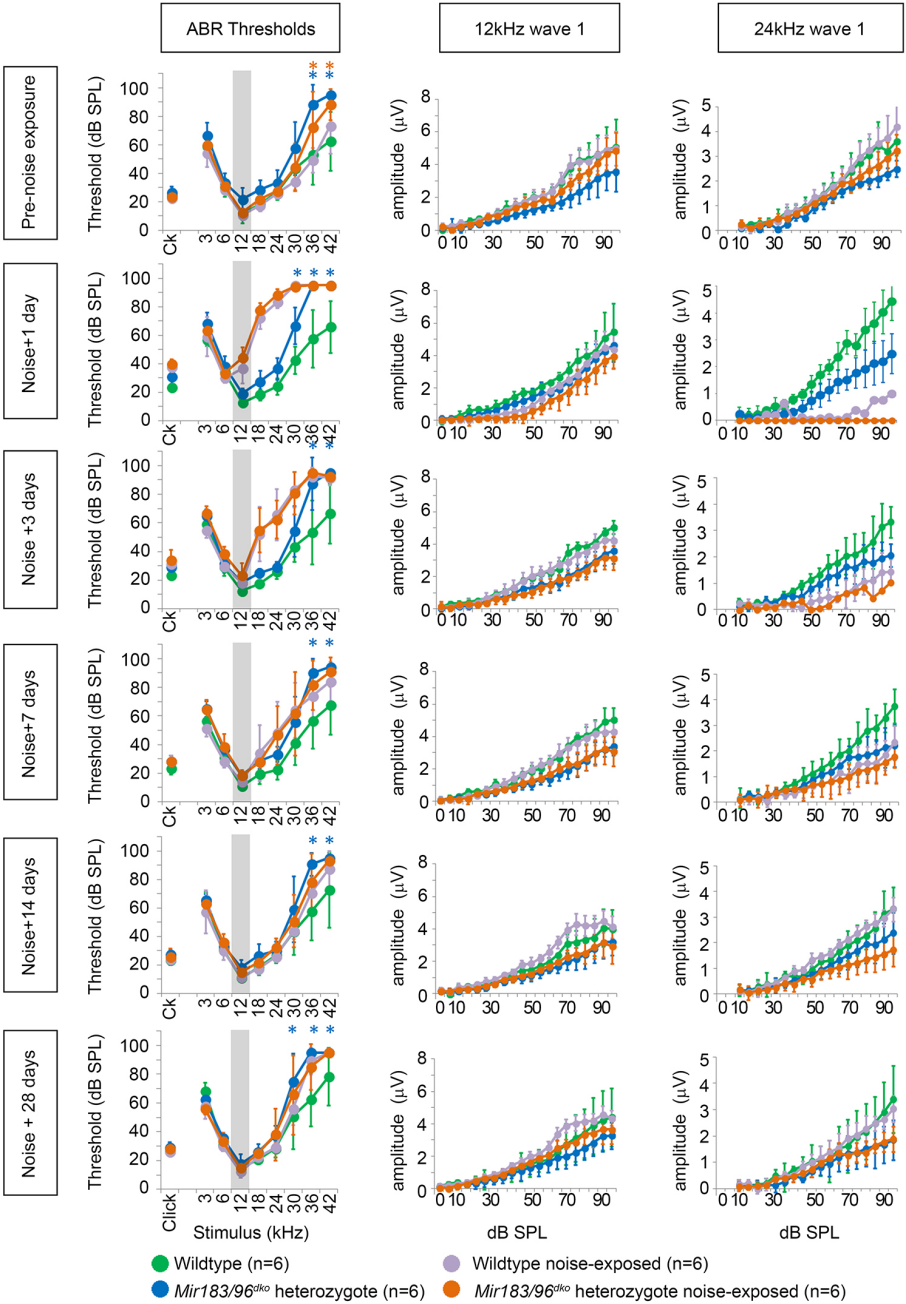
**Mean ABR thresholds and wave 1 amplitudes for wild-type and heterozygous *Mir183/96^dko^* mice before noise exposure and 1, 3, 7, 14 and 28 days after.** An increase in thresholds at 12 kHz and above is seen 1 day after exposure but thresholds have returned to normal by 28 days after (Bonferroni-corrected *P<0.05, mixed linear model pairwise comparison; asterisk is marked in blue for a significant difference between wild-type unexposed mice and heterozygous unexposed mice or orange for a significant difference between wild-type noise-exposed mice and heterozygous noise-exposed mice). Wave 1 amplitudes at 12 kHz and 24 kHz are shown for each time point. No obvious effect is visible at 12 kHz, but at 24 kHz, 1 day after noise exposure, wave 1 was too poorly defined to measure the amplitude in all heterozygotes and all but one wild type. By 28 days after exposure, both wild-type and heterozygote amplitudes have recovered to the normal range. Six wild-type mice were noise exposed (violet) with six unexposed controls (green), and six heterozygotes were noise-exposed (orange) with six unexposed controls (blue). Error bars are s.d. The grey area on the threshold plots indicates the octave band of noise (8-16 kHz). See Data S1 for the data and statistical analyses underlying these graphs.

As heterozygous *Mir183/96^dko^* mice showed no auditory deficit, in contrast to the hearing loss seen in diminuendo heterozygotes and in humans carrying one mutant *MIR96* allele, we asked whether these heterozygous mice might be more sensitive to noise-induced damage. One day after noise exposure at 8 weeks of age, both *Mir183/96^dko^* heterozygous and wild-type mice showed a marked increase in thresholds at 12 kHz and above compared with unexposed control littermates (Fig. 2). Three days after noise exposure the 12 kHz thresholds had recovered, but there was still a noticeable elevation at higher frequencies. By 7 days after exposure, all thresholds had recovered completely (Fig. 2). We measured the amplitude of wave 1 of the ABR waveform to look for a reduced neural response, which has been reported in CBA/CaJ mice after noise exposure (Kujawa and Liberman, 2009) and is thought to be due to neuronal loss in the cochlea, but no difference was observed at 12 kHz (Fig. 2). At 24 kHz, we observed a much greater effect, such that in most animals wave 1 was too poorly defined to measure the amplitude 1 day after exposure. However, both wild-type and heterozygote wave 1 amplitudes had recovered by 28 days after exposure, and did not look any different to the wave 1 amplitudes of unexposed mice (Fig. 2; Fig. S7).

Because there was a significant difference in the higher frequencies between the unexposed heterozygotes and wild types at 8 weeks of age (Fig. 2, top left panel), but we did not see any difference in our original ABR tests (Fig. 1, P56), we compared the ABR thresholds from all mice tested at 8 weeks old (Fig. S8). At high frequencies (30-42 kHz) the thresholds were very variable in both heterozygotes and wild types. The differences between the means at 36 kHz and 42 kHz are statistically significant (*P*=0.001, *P*=0.000; mixed linear model pairwise comparison), but, given the variability, we suggest that this is not biologically relevant.

### Hair cells and innervation

**Fig. 3.**
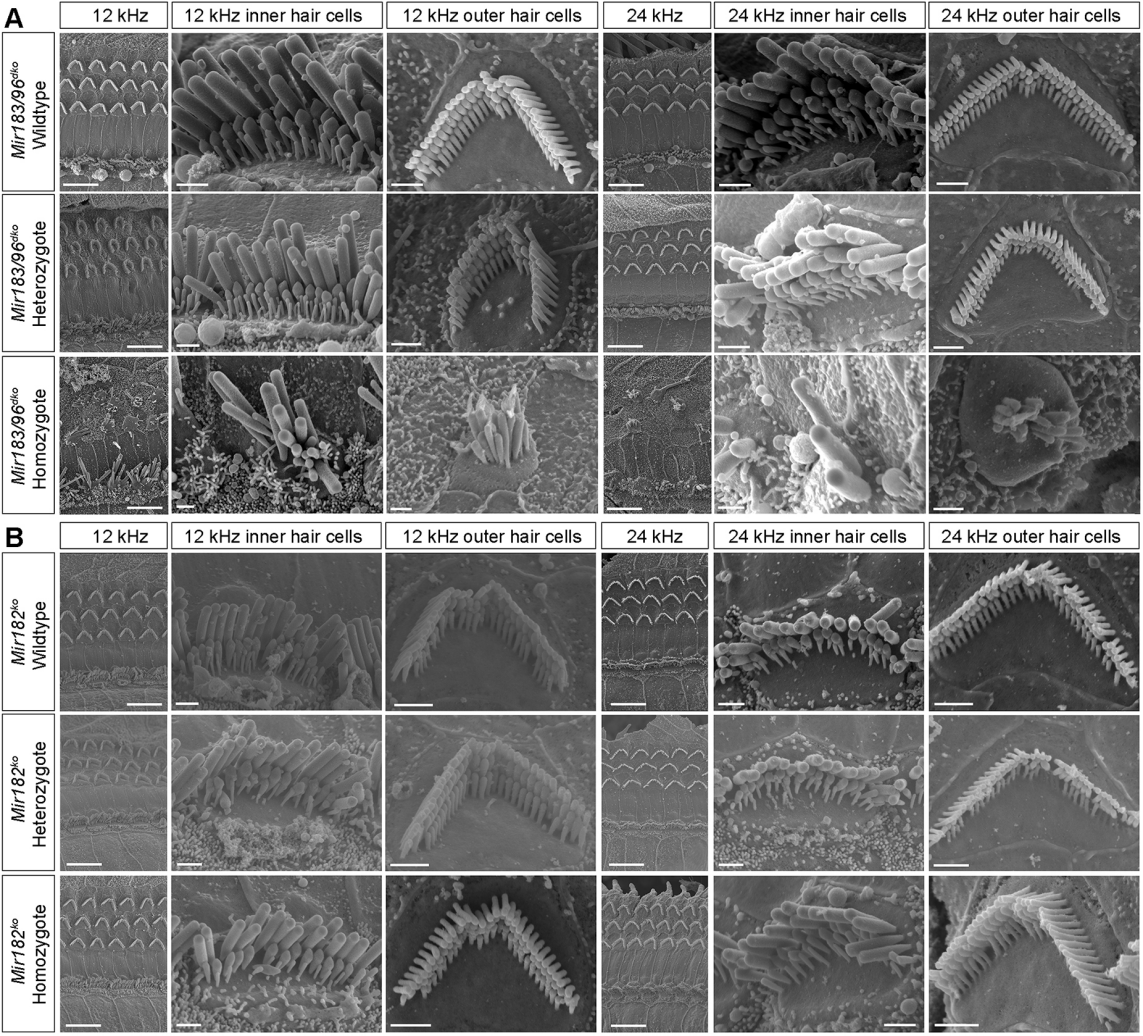
**Scanning electron micrographs of the organs of Corti of *Mir183/96^dko^* mice at P28 and *Mir182^ko^* mice at P56.** (A) *Mir183/96^dko^* mice at P28. (B) *Mir182^ko^* mice at P56. Two best-frequency regions of the organ of Corti are shown: 12 kHz and 24 kHz (68% and 43% of the way along the organ of Corti from base to apex, respectively). For each region, the left-hand column shows inner and outer hair cell rows (scale bars: 10 µm), and the other two columns show an inner and an outer hair cell close up (scale bars: 1 µm). (A) The top row shows wild-type hair cells (*n*=6 mice), the middle row shows heterozygote hair cells (*n*=6 mice) and the bottom row shows homozygote hair cells (*n*=5 mice). (B) The top row shows wild-type hair cells (*n*=1 mouse), the middle row shows heterozygote hair cells (*n*=2 mice) and the bottom row shows homozygote hair cells (*n*=3 mice).

We examined the organ of Corti using scanning electron microscopy and found that hair cells in homozygous *Mir183/96^dko^* mice were severely affected at 4 weeks of age (Fig. 3A), with many hair bundles missing entirely. Where present, the stereocilia bundles of both outer hair cells and inner hair cells (IHCs) show splaying and fusion. The IHCs of *Mir183/96^dko^* heterozygotes are unaffected, but the outer hair cells' upper surface appear slightly smaller and rounder in shape than the wild-type outer hair cells (Fig. 3A). Their stereocilia bundles also appear smaller and more rounded than normal, with more pronounced tapering in height and overlap of shorter stereocilia rows with taller rows towards the two ends of each bundle.

Four-week-old *Mir182^ko^* heterozygotes and homozygotes showed no abnormalities of hair cells by scanning electron microscopy at either the 12 kHz or 24 kHz regions (Fig. S9), corresponding to their normal ABR thresholds at that age. At 8 weeks old, when hearing loss is evident at 24 kHz and higher (Fig. 1), we also saw no systematic differences between wild types, heterozygotes and homozygotes (Fig. 3B).

**Fig. 4.**
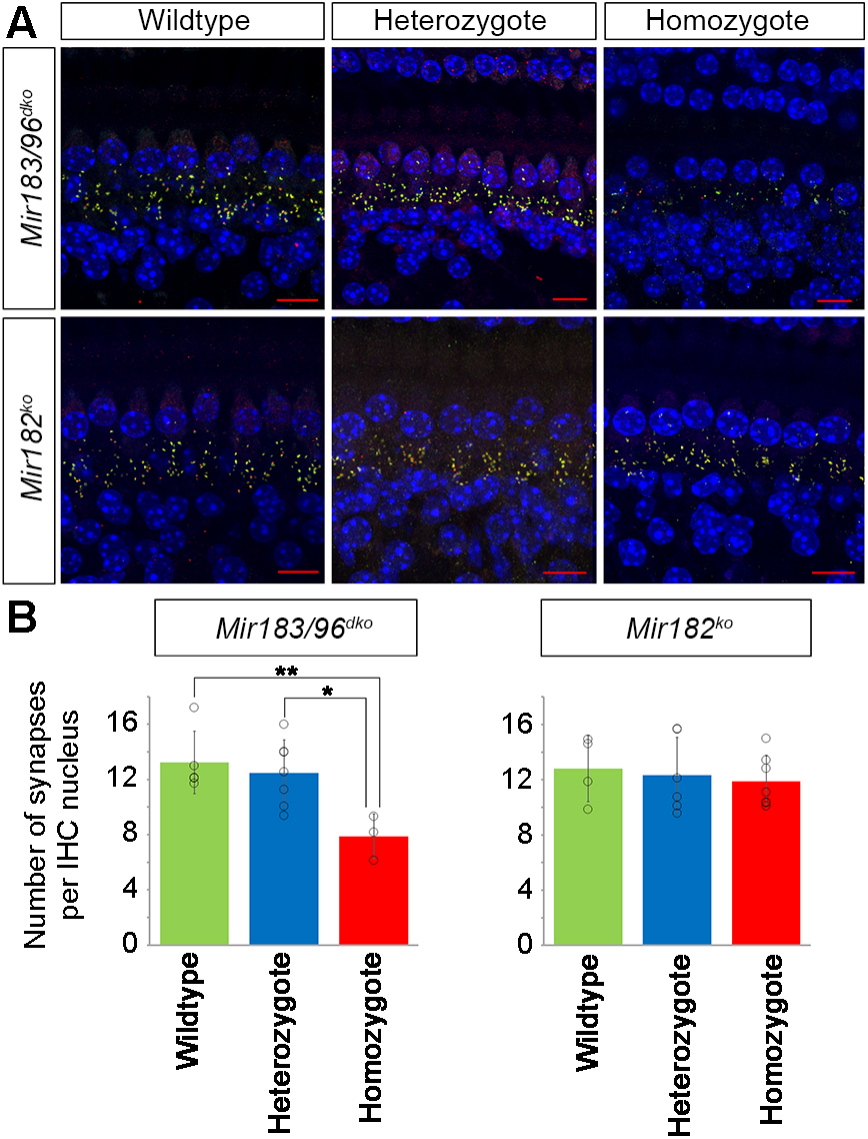
**Colocalised pre- and postsynaptic densities in *Mir183/96^dko^* and *Mir182^ko^* mice.** (A) Synapses below inner hair cells (IHCs) in *Mir183/96^dko^* (top) and *Mir182^ko^* (bottom) wild-type (left), heterozygous (middle) and homozygous (right) mice. Presynaptic ribbons are labelled with anti-Ribeye antibody (red) and postsynaptic densities with anti-GluR2 antibody (green); where they colocalise, the resulting colour is yellow. DAPI (blue) labelled the nuclei. Scale bar: 10 µm. (B) Mean counts of colocalised pre- and postsynaptic markers in wild-type (green), heterozygous (blue) and homozygous (red) *Mir183/96^dko^* (left) and *Mir182^ko^* (right) mice. There are significantly fewer colocalised synapses in *Mir183/96^dko^* homozygotes (*n*=3) compared with wild types (*n*=5, *P*=0.016, one-way ANOVA, Bonferroni-corrected **P=0.02) and also compared with heterozygotes (*n*=7, Bonferroni-corrected *P=0.035), but no significant differences in heterozygotes compared with wild types (Bonferroni-corrected *P*=1.0). No significant differences in synapse counts between *Mir182^ko^* knockout heterozygotes (*n*=6), homozygotes (*n*=7) and wild types (*n*=4, *P*=0.818, one-way ANOVA) were observed. Error bars show s.d. Individual datapoints are plotted as outlined circles for each group. See Data S1 for the data and statistical analyses underlying these graphs.

The distribution of unmyelinated neurons appeared normal in both mutants using anti-neurofilament labelling (Fig. S10). Synapses were examined using anti-Ribeye (also known as Ctbp2) antibody to mark presynaptic ribbons and anti-GluR2 (also known as Gria2) to mark postsynaptic densities, and a significant reduction was found in the number of colocalised pre- and postsynaptic markers in *Mir183/96^dko^* homozygotes (Fig. 4, *P*=0.016, one-way ANOVA). No difference in synapse counts was observed in *Mir182^ko^* homozygotes.

### Transcriptome analysis reveals misregulation of gene expression in mutants

To investigate the impact of the *Mir182^ko^* and *Mir183/96^dko^* mutations on gene expression, we carried out RNA sequencing (RNA-seq) of isolated organ of Corti preparations from P4 homozygotes and sex-matched littermate wild-type controls. This age was chosen to ensure that all hair cells were still present and to facilitate comparison with our previous transcriptome data from *Mir96^Dmdo^* mice (Lewis et al., 2009). Thirty-four genes were significantly misregulated [false discovery rate (FDR)<0.05] in *Mir183/96^dko^* homozygotes; of these, 22 were upregulated and 12 downregulated compared with wild-type littermates. Many of the upregulated genes have sequences complementary to either the miR-96 seed region or the miR-183 seed region in their 3′UTRs (Table 1). Of this list of 34 genes, only *Hspa2*, *Ocm*, *Myo3a*, *Slc26a5*, *Slc52a3*, *St8sia3* and *Sema3e* were previously found to be misregulated in *Mir96^Dmdo^* mice at P4 and/or P0 (Lewis et al., 2016, 2009), and in each case the misregulation is in the same direction (Table 1). We tested 19 genes of the 34, selected because they were reported to show a large difference in expression levels between sensory and non-sensory cells in the organ of Corti (http://www.umgear.org; Cai et al., 2015; Elkon et al., 2015). All but three were confirmed (Table 1; Fig. S11); those that were not were either not misregulated or failed the significance test. No genes were significantly misregulated in the opposite direction. Of the genes misregulated in *Mir183/96^dko^* homozygotes, five are known deafness genes; *Myo3a*, *Slc26a5* and *Tmc1* underlie deafness in mice and humans (Kurima et al., 2002; Liberman et al., 2002; Liu et al., 2003; Vreugde et al., 2002; Walsh et al., 2002, 2011), while mutations in *Sema3e* cause deafness in people (Lalani et al., 2004), and mice lacking *Ocm* exhibit progressive hearing loss (Tong et al., 2016).

**Table 1.**
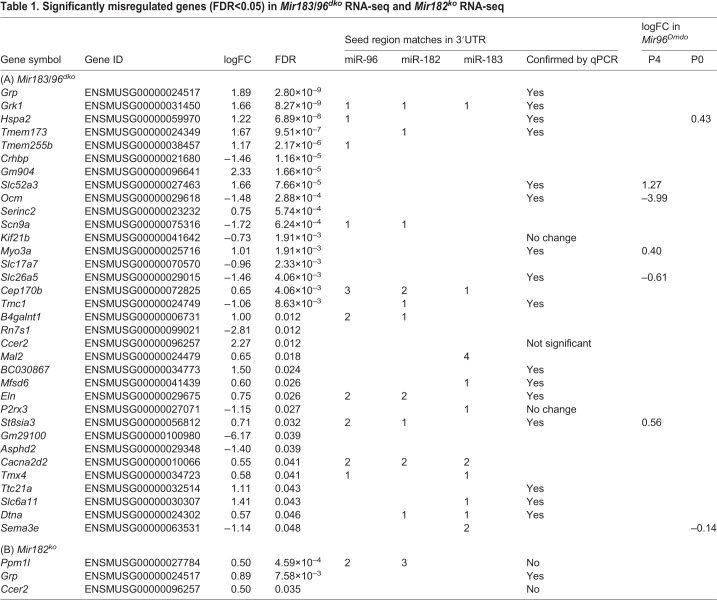
**Significantly misregulated genes (FDR<0.05) in *Mir183/96^dko^* RNA-seq and *Mir182^ko^* RNA-seq**

It is possible that the difference between the transcriptomes of *Mir183/96^dko^* knockout mice and *Mir96^Dmdo^* mice is caused by the different backgrounds. We looked for differences in predicted targets in the 3′UTRs of the C57BL/6NJ and C3H/HeJ genome sequences (Adams et al., 2015), which were the closest genome sequences to our mutant backgrounds available, and found that 1585 genes had the same number of seed matches, and 13 genes had seed matches in both strains, but not the same number of matches. Thirty-six genes had no seed matches in the C57BL/6NJ sequence but had one or more match in the C3H/HeJ sequence, and 47 genes had no seed matches in the C3H/HeJ sequence, but one or more in the C57BL/6NJ sequence (Table S1).

Three genes were found to be significantly upregulated (FDR<0.05) in the *Mir182^ko^* homozygotes by RNA-seq, one of which, *Ppm1l*, has sequences complementary to the seed region of miR-182 (Table 1). The other two, *Grp* and *Ccer2*, were also upregulated in the *Mir183/96^dko^* homozygotes. No genes were significantly downregulated in *Mir182^ko^*. We tested the upregulated genes by qPCR, and also tested *Slc26a5* and *Ocm* [which were strongly downregulated in *Mir96^Dmdo^* homozygotes (Lewis et al., 2009)] and found that only the upregulation of *Grp* was confirmed. *Ccer2* and *Ppm1l* were upregulated but not significantly, and *Slc26a5* and *Ocm* were downregulated but, again, not significantly (Table 1; Fig. S11).

**Fig. 5.**
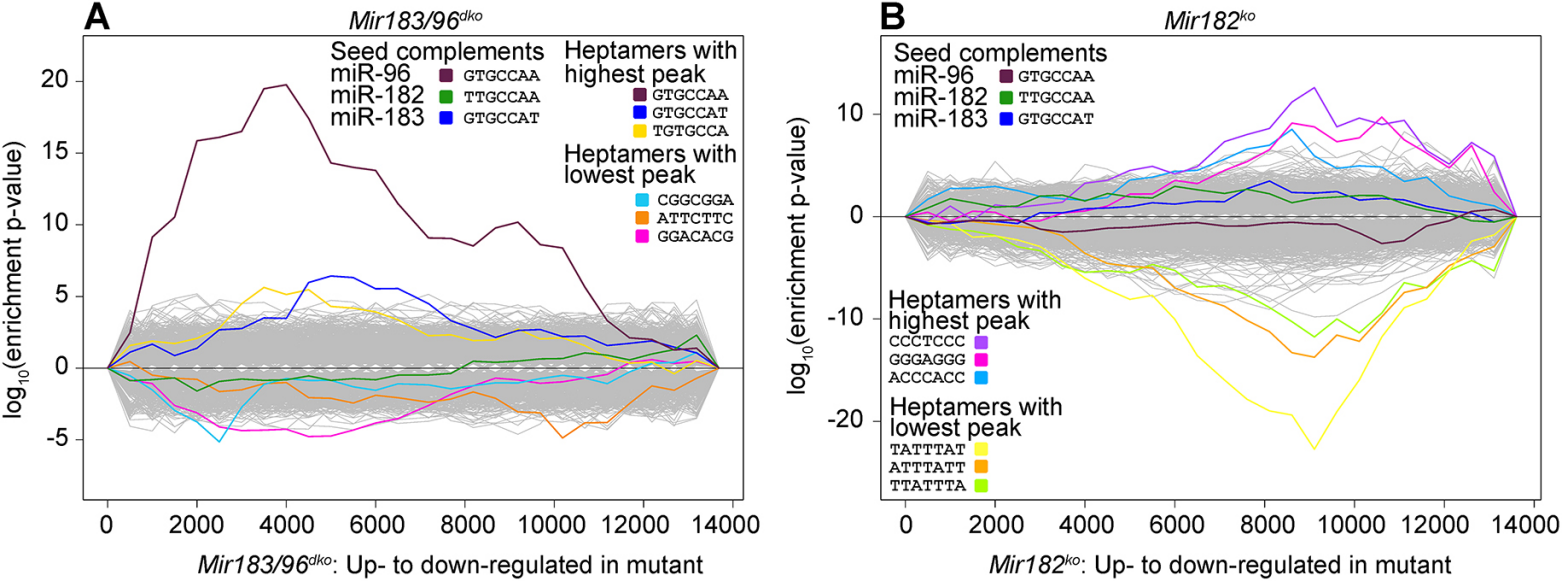
**Sylamer analysis showing enrichment and depletion of heptamers in 3′UTRs in RNA-seq data from *Mir183/96^dko^* homozygous mice and *Mir182^ko^* homozygous mice.** (A) *Mir183/96^dko^* homozygous mice. (B) *Mir182^ko^* homozygous mice. The *x*-axis represents the sorted gene list from most upregulated on the left to most downregulated on the right. The *y*-axis shows the hypergeometric significance for enrichment or depletion of heptamers in 3′UTRs. UTRs are considered in bins, starting with the 500 most upregulated genes and increasing cumulatively until all genes have been considered. Lines indicate the enrichment of each heptamer; positive values indicate enrichment and negative values indicate depletion. The three heptamers with the highest peaks and the three with the lowest peaks are highlighted in colour, as are the three heptamers complementary to the seed regions of miR-96, miR-182 and miR-183. The yellow line in the *Mir182^ko^* plot on the right, enriched in the downregulated genes of *Mir182^ko^* homozygotes, resembles a portion of an AU-rich element.

To assess the impact of these miRNA knockouts on a genome-wide level, we used Sylamer (van Dongen et al., 2008) to measure the enrichment and depletion of all possible heptamers in the 3′UTRs of each total gene list, ranked from most upregulated to most downregulated irrespective of significance. In the *Mir183/96^dko^* gene list, the sequence complementary to the seed region of miR-96 was markedly enriched in the upregulated genes (maroon line, Fig. 5A), and the sequence complementary to the seed region of miR-183 has a small peak towards the centre of the graph; although the targets of miR-183 are not notably misregulated in this dataset, its signal is still distinct from all other miRNAs (dark-blue line, Fig. 5A). There were no miRNA seed region heptamers enriched in the *Mir182^ko^* gene list (Fig. 5B), but the TATTTAT heptamer that is enriched in the *Mir182^ko^* downregulated genes (yellow line, Fig. 5B) resembles a portion of an AU-rich element. These are 50-150 bp sequence elements found in 3′UTRs, and are typically involved in mRNA destabilising via a deadenylation-dependent mechanism (reviewed in Barreau et al., 2005; Chen and Shyu, 1995). AU-rich elements, and TATTTAT in particular, are enriched in 3′UTR regions that have multiple RNA-binding protein binding sites, and it has been suggested that RNA-binding proteins compete with the RNA-induced silencing complex to bind to miRNA target sites within these regions (Plass et al., 2017). It is possible that this TATTTAT signal, which is enriched in the genes downregulated in *Mir182^ko^* homozygote hair cells, is the result of a change in the binding of RNA-binding proteins in the absence of miR-182.

### No evidence for differential splicing

MicroRNAs can affect splicing if they target a splicing factor (Boutz et al., 2007), and splicing factors are known to be important for hair cell development (Nakano et al., 2012). We looked for evidence of differential splicing in both RNA-seq datasets using three different tools [Cuffdiff (Trapnell et al., 2010), JunctionSeq (Hartley and Mullikin, 2016) and Leafcutter (Li et al., 2018)], and followed up the predictions (Table S2) by resequencing cDNA from the organ of Corti in homozygotes and wild types (Table S3). We found no evidence of the predicted differential splicing, but a novel isoform of *Stard9*, present in both wild-type and homozygous *Mir183/96^dko^* cDNA, was detected by JunctionSeq (Fig. S12).

### Immunohistochemistry confirms downregulation of *Ocm*

We carried out antibody stains on sections from the inner ear at P4, to check for the presence of Ocm (oncomodulin) and Slc26a5 (prestin) protein in the hair cells. Ocm staining is faint at P4, stronger in the basal turn of the cochlea, but prestin staining is clearly visible at that stage in wild types. We observed prestin staining in *Mir183/96^dko^* homozygotes, but no stain for Ocm, while both proteins were present in the wild-type littermate controls (Fig. S13). Although immunohistochemistry is not a quantitative technique, this correlates with the qPCR results, which showed that *Ocm* RNA was nearly absent in *Mir183/96^dko^* homozygotes, whereas *Slc26a5* RNA levels were ∼30% of wild-type levels (Fig. S11). Ocm and Prestin staining was visible in *Mir182^ko^* homozygotes and wild-type littermates (Fig. S13).

### Network analysis

In order to explore the network of regulatory interactions controlled by miR-96, we took three approaches to network construction and analysis. First, we used Ingenuity Pathway Analysis (IPA) to predict upstream regulators that could explain the misregulation we observed in our RNA-seq data. Second, we used weighted gene correlation network analysis to explore modules of co-expressed genes for which changes in expression correlated with the sample genotypes. Third, we used publicly available regulatory data to build a network of potential interactions linking miR-96 to the misregulated genes.

### Network analysis: IPA suggests upstream regulators

IPA was used to construct and score putative causal networks using the misregulated genes in each dataset with FDR<0.05, based on a manually curated set of observations from the literature and third-party databases. For the *Mir183/96^dko^* homozygotes, we obtained eight networks for which their upstream regulators were given a significant or nearly significant *z*-score; Bdnf, Gh, H2afx (also known as H2ax), Kcna3, Kit, Myocd, Prkaca and Pth, which explain between them the misregulation of 14 genes (Fig. S14). Two of these, *Bdnf* and *Kit*, are known deafness genes (Agerman et al., 2003; Deol, 1970). None of the eight are misregulated in the RNA-seq data, and all but one are predicted to be inhibited, so they cannot be directly linked to miR-96 [including the known miR-96 target, Bdnf (Li et al., 2015)]. Only Kcna3 is predicted to be activated (orange in Fig. S14), and it does not have any matches to the miR-96 or miR-183 seed regions in its 3′UTR. The lack of a connection to miR-96 could be because the causal networks have a maximum depth of 3, which might not be enough to reach the level of direct targets.

There were too few genes significantly misregulated in the *Mir182^ko^* RNA-seq data to obtain any causal networks with a significant activation *z*-score, but Ret and Adcyap1 were identified as immediate upstream regulators of Grp.

### Network analysis: gene clustering using weighted gene correlation network analysis (WGCNA) suggests further regulators

WGCNA is a method of analysing transcriptome data to cluster genes into modules based on their expression across a number of individual samples without reference to the sample traits (Zhang and Horvath, 2005). We used a Pearson correlation to cluster genes across all 24 samples (*Mir183/96^dko^* and *Mir182^ko^*) and obtained 29 consensus modules (including the reserved ‘grey’ module, which consists of genes outside all the other modules) (Table S4).

These consensus modules are groups of genes with highly correlated expression profiles. We calculated the correlation of the modules with each other and with the traits of the mice used for RNA-seq (Fig. S15), and found that three modules were highly correlated and clustered with the wild type versus *Mir183/96^dko^* homozygote trait (green, black, royal blue, Fig. S16A). This means that the expression levels of the genes of the green, black and royal blue modules are correlated with each other and the genotypes of the *Mir183/96^dko^* mice. One module was highly correlated and clustered with the wild type versus *Mir182^ko^* homozygote trait (salmon, Fig. S16B).

We chose nine modules for further exploration (Fig. S15, Table S4). We carried out enrichment analysis for gene ontology (GO) biological processes (Ashburner et al., 2000; The Gene Ontology Consortium, 2019) and Reactome pathways (Fabregat et al., 2018) using PANTHER v14 (Mi et al., 2019). The GO terms and Reactome pathways identified suggest the involvement of mRNA processing, transcriptional regulation and cell metabolism. Notably, the blue module, which contained the most differentially expressed genes, was linked to the GO terms ‘auditory receptor cell fate commitment’ and ‘inner ear receptor cell fate commitment’ (see Tables S5 and S6 for full listings of enriched GO terms and Reactome pathways).

We also carried out a transcription factor binding site enrichment analysis using oPOSSUM (Kwon et al., 2012), and identified 29 potential transcription factor binding site profiles (Fig. S17, Table S4), several of which were shared between modules (Fig. S18). Transcription factors implicated in the modules include two deafness genes [*Foxi1* (Hulander et al., 1998), *Myc* (Wei et al., 2007)] and several genes with miR-183/96/182 seed region matches, although none have yet been experimentally shown to be targets of any of the three miRNAs (Table S7), and none were misregulated in the RNA-seq data. This approach highlighted multiple transcription factors that could be involved; however, it did not offer any links to miR-96 or the significantly misregulated genes.

### Network analysis: an automated approach using publicly available regulatory data

In our previous study on genes misregulated in *Mir96^Dmdo^*, we used regulatory interactions described in the literature to create an internally consistent network of regulatory interactions connecting miR-96 to as many of the misregulated genes as possible (Lewis et al., 2016). For the current study, we automated the procedure and made use of publicly available regulatory data in addition to the manually curated regulatory data from the literature we compiled before.

**Fig. 6.**
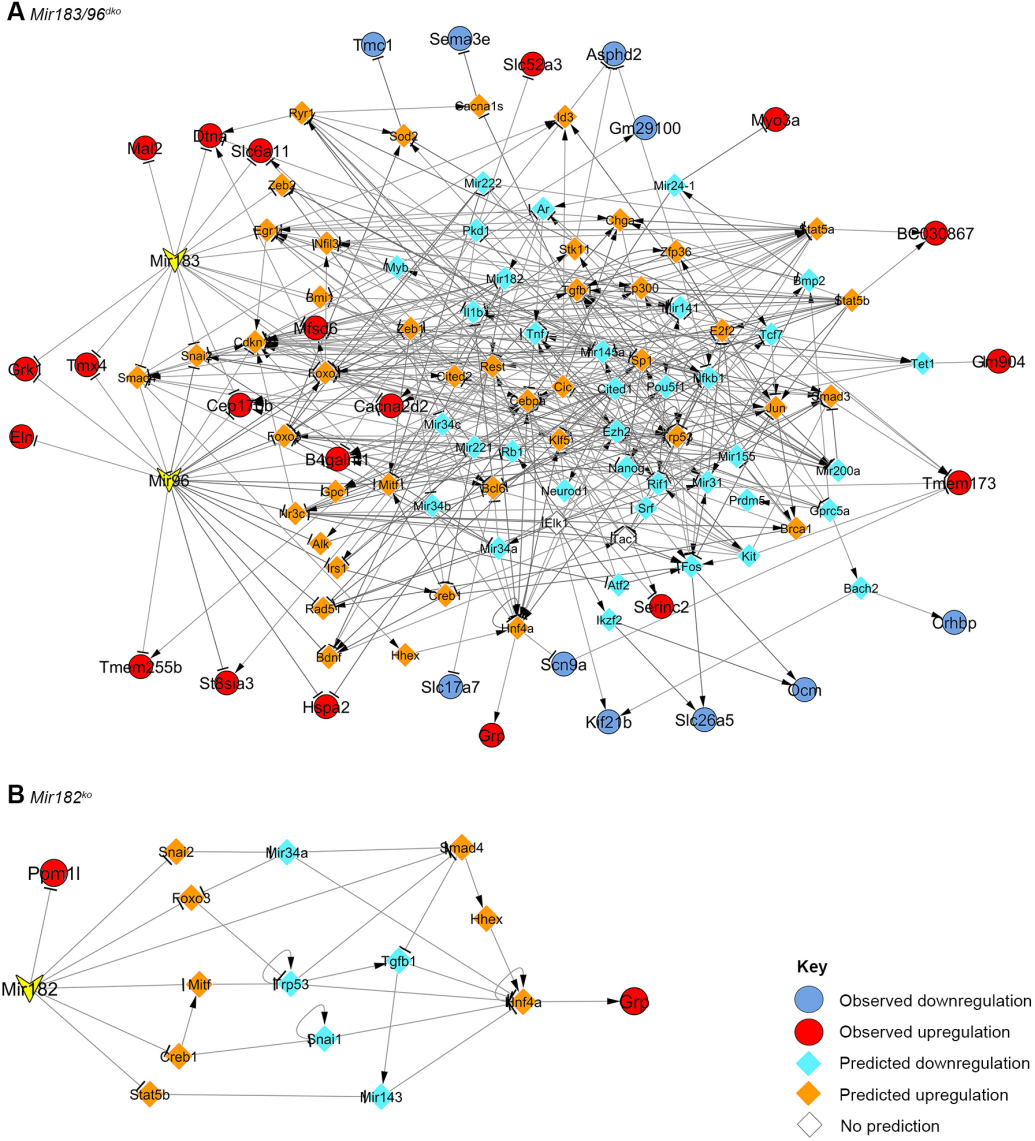
**Networks created using publicly available regulatory data, based on the *Mir183/96^dko^* and *Mir182^ko^* transcriptome data.** (A) *Mir183/96^dko^* transcriptome data. (B) *Mir182^ko^* transcriptome data. Red and orange indicate upregulation; blue and turquoise indicate downregulation; circles with black borders show known misregulation in the organ of Corti; diamonds without borders show predicted misregulation. The yellow arrowheads denote the three miRNAs: *Mir96*, *Mir182* and *Mir183*. See Data S1 for network data and references.

The resulting *Mir183/96^dko^* network consists of 114 genes and 416 links. Thirty misregulated genes have been included, 13 of them predicted targets of either miR-183 or miR-96 or both. Two misregulated genes were left out completely due to having no known upstream regulators in our compiled regulatory interactions (*Ccer2* and *Rn7s1*) (Fig. 6A). The gene with the most interactions is *Trp53*, with 23 interactions. Most genes have nine or fewer interactions (Fig. 6A), but 34 have ten or more, including seven that are direct targets of miR-96, miR-183 or both (listed in Table S8). None of these 34 genes with ten or more interactions are known to be misregulated in *Mir183/96^dko^* homozygotes, but eight are known deafness genes: *Fos* (Paylor et al., 1994), *Foxo3* (Gilels et al., 2013), *Kit* (Deol, 1970), *Mir96*, *Nfkb1* (Lang et al., 2006), *Pkd1* (Steigelman et al., 2011), *Rest* (Nakano et al., 2018) and *Tnf* (Oishi et al., 2013).

The *Mir182^ko^* network is very small, being based on three input genes: *Ppm1l*, *Ccer2* and *Grp*. *Ccer2* could not be included, owing to the lack of any known regulators, and *Ppm1l* is a predicted direct target of miR-182. Multiple potential pathways link *Grp* to miR-182, but all of them work through *Hnf4a*, which upregulates *Grp* (Fig. 6B).

To validate the network analyses, we carried out quantitative PCR (qPCR) on 11 genes from the *Mir183/96^dko^* network and two from the *Mir182^ko^* network (Fig. 6). We found that all the genes had very variable expression levels in homozygotes compared with littermate wild types (Fig. S11). It is possible that this is the result of expression in different areas of the inner ear. The inner ear is a complex organ with many cellular subtypes, and small changes in mRNA levels in the sensory tissue alone may be hard to detect in bulk cDNA. In addition, qPCR can only detect mRNA levels, and cannot directly measure protein levels or protein activity.

## DISCUSSION

### *Mir183*/*96^dko^* mice have a less-severe phenotype than *Mir96^Dmdo^* mice

Mice heterozygous for the *Mir96^Dmdo^* point mutation exhibit early-onset rapidly progressive hearing loss; even at P15 they have very raised thresholds (Kuhn et al., 2011). We initially concluded that this was more likely to be due to haploinsufficiency than to the gain of novel targets, because humans heterozygous for different point mutations also had progressive hearing loss (Mencia et al., 2009). However, heterozygous *Mir183/96^dko^* mice have ABR thresholds and DPOAE responses resembling those of the wild type, and there is no difference in thresholds or wave 1 amplitudes between heterozygotes and wild types after noise exposure. Two recent studies of different knockout alleles targeting the entire miRNA cluster (miR-183/96/182) found that heterozygotes also exhibited normal hearing (Fan et al., 2017; Geng et al., 2018). It is possible that the more-severe phenotype seen in *Mir96^Dmdo^* heterozygotes is due to the acquisition of new targets by the mutant miRNA, or it could be an effect of the different background, as the *Mir96^Dmdo^* allele was generated by N-ethyl-N-nitrosourea (ENU) mutagenesis on a C3HeB/FeJ background, in contrast to the C57BL/6N background of the *Mir183/96^dko^* allele or the reported knockouts of the entire cluster, which were created in 129S2 (Lumayag et al., 2013) or 129SV6 (Fan et al., 2017) embryonic stem (ES) cells and then crossed onto the C57BL/6J background (Fan et al., 2017; Lumayag et al., 2013). Mice homozygous for the *Mir183/96^dko^* allele showed no ABRs at all ages tested from 14 days onwards, and in this they resemble the *Mir96^Dmdo^* homozygotes, in which the compound action potentials recorded from the round window of the cochlea were undetectable at 4 weeks of age (Lewis et al., 2009).

*Mir96^Dmdo^* homozygotes circle by 3 weeks of age, whereas a milder phenotype of variable hyperactivity was observed in some *Mir183/96^dko^* homozygotes, the prevalence increasing with age (Fig. S5). Circling is a more-severe manifestation of vestibular dysfunction than hyperactivity. This could again be due to the C3HeB/FeJ background, because *Mir96^Dmdo^* mice carry the *Pde6b^rd1^* mutation causing retinal degeneration (Pittler and Baehr, 1991) and are blind by adulthood. The *Mir183/96^dko^* allele was generated on a C57BL/6N background, which lacks the *Pde6b^rd1^* mutation but has the *Crb1rd^8^* mutation, which leads to variably penetrant retinal dysplasia (Moore et al., 2018). Other ocular abnormalities have also been observed in C57BL/6N mice, such as lens abnormalities and vitreous crystalline deposits (Moore et al., 2018). However, no ocular abnormalities have been reported for these mice in the International Mouse Phenotyping Consortium (IMPC) pipeline (https://www.mousephenotype.org/data/genes/MGI:3619440), so it is likely that they have sufficient vision to partially compensate for the lack of vestibular sensory input, reducing the severity of the observed phenotype. It is notable that mice lacking the entire miR-183/96/182 cluster (on a mixed background of C57BL/6J and either 129S2 or 129SV6) exhibit both persistent circling behaviour and retinal defects (Fan et al., 2017; Lumayag et al., 2013).

### Stereocilia bundles and innervation in *Mir183*/*96^dko^* mice and *Mir96^Dmdo^* mice

The hair cells of *Mir96^Dmdo^* homozygous mice are present at 4 days old but appear abnormal, and by 28 days old have degenerated almost completely (Lewis et al., 2009). In *Mir183/96^dko^* homozygotes, however, some hair cell stereocilia bundles are still visible at P28, although they are severely disorganised (Fig. 3A). In *Mir183/96^dko^* heterozygotes, IHC stereocilia are mostly normal and the OHC stereocilia bundles appear to be slightly rounded in arrangement (Fig. 3A), but this is not reflected in their ABR thresholds or DPOAE responses, which are normal (Fig. 1; Fig. S5). *Mir96^Dmdo^* heterozygotes, which are deaf by P28, have a more-severe phenotype, with fused stereocilia and hair cell degeneration as well as misshapen stereocilia bundles in outer hair cells, and smaller stereocilia bundles in IHCs (Lewis et al., 2009).

*Mir96^Dmdo^* homozygotes exhibit disorganised innervation (Kuhn et al., 2011), but we did not see that degree of disorganisation in neurofilament-labelled preparations of *Mir183/96^dko^* homozygotes (Fig. S10). However, we found significantly fewer colocalised pre- and postsynaptic densities under IHCs of *Mir183/96^dko^* homozygotes indicating synaptic defects (Fig. 4). Immature IHC ribbon shapes have previously been reported in *Mir96^Dmdo^* homozygotes (Kuhn et al., 2011), but the synapses have not been quantified in the same way so cannot be directly compared. *Mir183/96^dko^* heterozygotes showed no differences in innervation or synapse counts compared with wild types (Fig. 4; Fig. S10). Similar to the physiological phenotype, the structural phenotype of *Mir183/96^dko^* heterozygotes is much less severe than that of *Mir96^Dmdo^* heterozygotes.

### Fewer genes are affected in the *Mir183/96^dko^* transcriptome than in the *Mir96^Dmdo^* transcriptome

The *Mir183/96^dko^* transcriptome bears some resemblance to that of the *Mir96^Dmdo^* transcriptome from our previous studies (Lewis et al., 2016, 2009), but, as with the physiological and structural phenotypes, the effect of missing both miR-183 and miR-96 appears to be milder than the effect of a point mutation in the miR-96 seed region. Only 34 genes were identified as significantly misregulated by RNA-seq in the current study of *Mir183/96^dko^*, compared with 86 genes found to be significantly misregulated in the *Mir96^Dmdo^* P4 microarray (Lewis et al., 2009), and only seven genes are similarly misregulated in both mutants: *Hspa2*, *Ocm*, *Myo3a*, *Slc26a5*, *Slc52a3*, *St8sia3* and *Sema3e* (Table 1). Some of the differences will be due to certain genes not being represented on the microarray assay when it was carried out, such as *Ccer2*, and some may be the result of the different genetic background. We aimed to minimise this by use of the sex-matched wild-type littermates as controls, but it is not possible to entirely eliminate the effect of genetic background; there are 83 genes that lack miR-96 seed region matches in one background compared with the other (Table S1), any of which could be contributing to the phenotype. Much of the difference, however, is likely to be due to the loss of miR-183 in the *Mir183/96^dko^* homozygotes and the presence of the mutant miR-96 in *Mir96^Dmdo^* homozygotes.

### Network analyses of the *Mir183/96^dko^* transcriptome suggest multiple potential regulators

We took three approaches to find intermediate regulators, and obtained eight upstream regulators from the IPA causal network analysis, 29 transcription factor profiles from the WGCNA module analyses (Table S7), and 34 highly connected nodes from our regulatory data-based network construction (Table S8). There are no genes suggested by all three approaches, although each one suggests multiple plausible candidates, such as *Fos*, *Kit*, *Foxi1*, *Myc*, *Zeb1* and *Foxo1*. However, when we tested a selection of intermediate genes, we found that their expression was very variable between different homozygotes (Fig. S11). This was true even of *Ikzf2*, which is known to directly regulate *Ocm* and *Slc26a5* (Chessum et al., 2018), two critical genes for outer hair cell function, which are strongly downregulated in both *Mir96^Dmdo^* (Lewis et al., 2016, 2009) and *Mir183/96^dko^*.

### Identifying candidate direct targets of miR-96 in hair cells

The only network approach that suggested direct targets of miR-96 in the inner ear was our regulatory data-based approach. It suggested 21 direct targets of miR-96 in total, ten of which were upregulated in the RNA-seq data. We used the gEAR dataset comparison tool (https://umgear.org/compare_datasets.html, accessed July 2020) and data from mouse hair cells compared with the rest of the cochlear duct at P0 (Cai et al., 2015) to identify which of the 21 direct targets were excluded from hair cells but present in the rest of the cochlear duct. We found eight genes that showed this pattern of expression: *Snai2*, *Zeb1*, *Irs1*, *Nr3c1*, *Foxo1*, *Alk*, *Eln* and *Rad51*. Three of these are known deafness genes: *Snai2*, which is involved in melanocyte development (Sanchez-Martin et al., 2002), *Zeb1*, which is required for the specification of mesenchymal identity and repression of epithelial identity (Hertzano et al., 2011), and *Irs1* (DeMambro et al., 2010). The precise role of Irs1 in the function of the cochlea has not yet been elucidated, but because Zeb1 and Snai2 are known to be required for the development of non-sensory cells in the cochlear duct, it may be that miR-96 plays a role in establishing and/or maintaining the repression of non-hair cell genes in developing hair cells.

*Nr3c1* and *Foxo1* have the most downstream links of the 21 direct targets, predicted to regulate nine and 12 genes, respectively*. Foxo1* encodes a forkhead family transcription factor, and the *Nr3c1* gene encodes the glucocorticoid receptor GR, which is known to be expressed in the inner ear (Erichsen et al., 1998; Ten Cate et al., 1993). No hearing or vestibular phenotypes have been reported on the Mouse Genome Informatics (MGI) resource (http://www.informatics.jax.org, accessed October 2020) (Smith et al., 2018) for mice carrying mutations in either gene, but it is likely that the hearing of *Nr3c1* mutants has simply never been checked. The hearing of *Foxo1* knockout heterozygotes was reported as normal in the IMPC phenotyping pipeline (http://www.mousephenotype.org; Dickinson et al., 2016), but because the knockout is homozygous lethal, there are no data for the effect of the absence of *Foxo1* on the inner ear.

Only one of these eight targets was upregulated in *Mir183/96^dko^* homozygotes in the RNA-seq data. Eln is a connective tissue protein and a component of the extracellular matrix (Daamen and Quaglino, 2019), and plays a regulatory role in controlling vascular smooth muscle cells via a G-protein-coupled receptor pathway (Karnik et al., 2003), but its downstream targets, and its role in the inner ear, are not yet known.

### The gain of novel targets plays an important role in the phenotype resulting from a point mutation in *Mir96*

The difference between the *Mir96^Dmdo^* and *Mir183/96^dko^* transcriptomes, along with the less-severe phenotype including normal ABR thresholds of *Mir183/96^dko^* heterozygotes, suggests that the gain of novel target mRNAs is important for the diminuendo phenotype. From the microarray carried out on *Mir96^Dmdo^* P4 organ of Corti, we found 19 genes that were significantly downregulated in the mutant and that bore matches to the mutant seed region in their 3′UTR (Lewis et al., 2009). The list includes one known deafness gene, *Ptprq* (Goodyear et al., 2003), as well as *Chrna1*, which is expressed in the organ of Corti from early postnatal stages onwards (Roux et al., 2016). The hair cells of mice homozygous for a null allele of *Ptprq* closely resemble those seen in the *Mir96^Dmdo^* homozygotes and heterozygotes at P4 (Chen et al., 2014), so it is possible that the more-severe phenotype seen in *Mir96^Dmdo^* homozygotes is caused, in part, by the downregulation of *Ptprq* by the mutant miR-96.

### A model for mechanisms of action of mutant miRNAs

It has been suggested that miRNAs act in two ways; first, they repress targets to prevent translation, resulting in mutually exclusive expression of the miRNA and the target, and second, they buffer transcriptional noise, in which case the miRNA and its targets are co-expressed in the same cell (Hornstein and Shomron, 2006). Our approach should be able to detect both effects, but if a target is highly expressed in the non-sensory epithelial cells, the difference in expression between wild type and homozygote as a result of derepression in the hair cells might not be detectable. Our current transcriptome data, therefore, are more likely to highlight targets that are being buffered rather than targets that are completely repressed by miR-96 (Hertzano et al., 2011). This may explain why likely target genes, such as *Zeb1*, *Foxo1* and *Nr3c1*, have not been found to be significantly misregulated in our transcriptome data. It may also explain the variability of the network genes we tested (Fig. S11E,F) – in the absence of the miRNA buffering, transcriptional noise has increased. This effect would be exacerbated by a mutated seed region, such as in the *Mir96^Dmdo^* mutant; not only would the normal buffering effect be gone, but multiple other genes would be misregulated within the hair cell, further disrupting normal cell function, as indeed we observed in the *Mir96^Dmdo^* transcriptome analyses (Lewis et al., 2016, 2009).

**Fig. 7.**
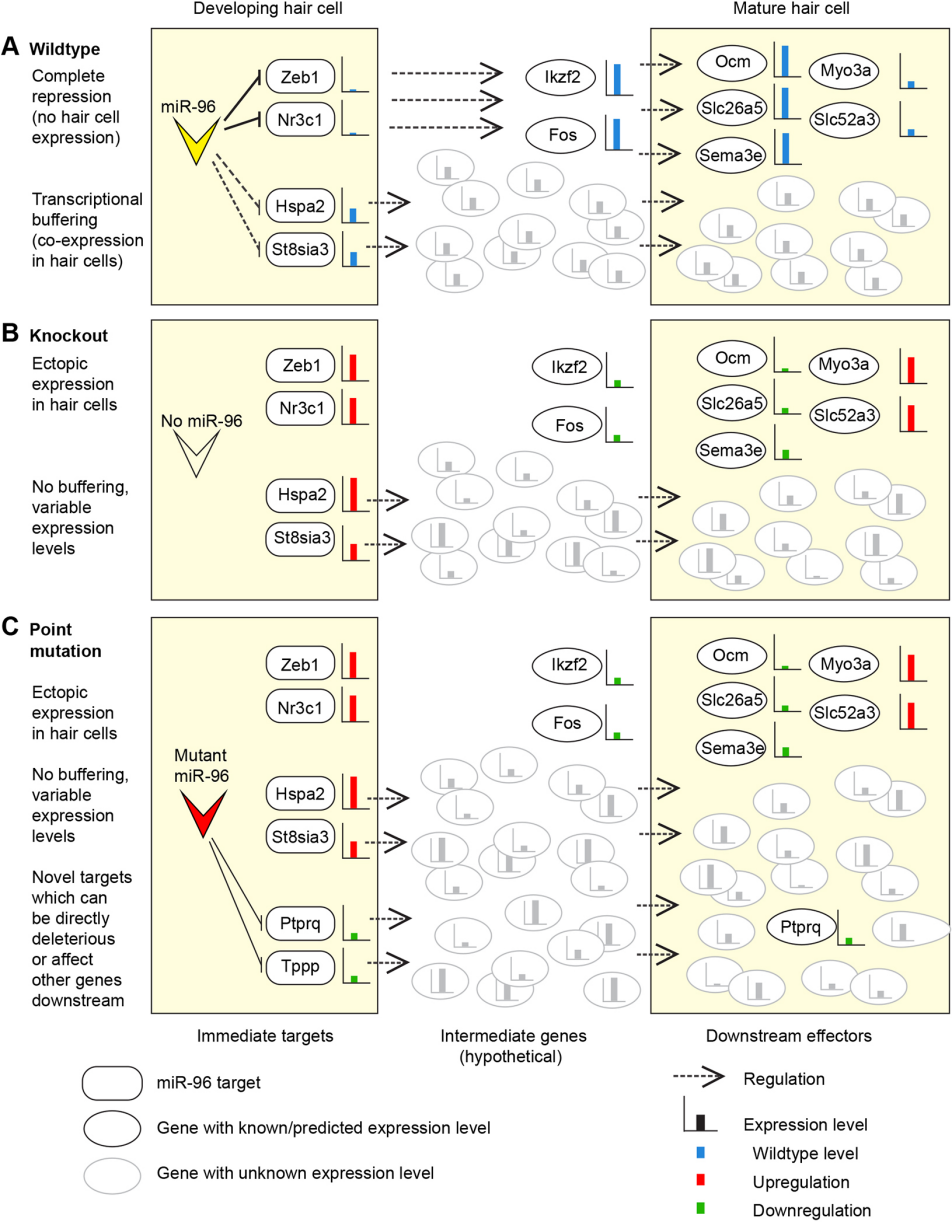
**Diagram of the modes of action of mutant miRNAs in the outer hair cell.** (A) In a wild-type hair cell, miR-96 represses some genes completely (here represented by *Zeb1* and *Nr3c1*) and buffers the expression levels of other genes (*Hspa2*, *St8sia3*). The result is a mature, functional outer hair cell with wild-type expression levels of important genes (e.g. *Ocm*, *Slc26a5*). (B) In the absence of miR-96, as in the *Mir183/96^dko^* homozygote, there is both ectopic expression of targets such as *Zeb1* and *Nr3c1*, and variable expression of the buffered targets such as *Hspa2* and *St8sia3*. This leads to very variable expression levels of many genes (shown in grey), and the downregulation of genes critical for outer hair cell function, such as *Ocm* and *Slc26a5*. (C) If the miRNA bears a point mutation, as in the *Mir96^Dmdo^* homozygote, then a third effect comes into play, namely the downregulation of genes that bear a match to the mutant seed region in their 3′UTR. In the case of *Mir96^Dmdo^*, this includes *Ptprq*, which is an essential gene for stereocilia bundle development (Goodyear et al., 2003). Many other genes (shown in grey) will also be misregulated through the gain of novel targets, further contributing to the functional failure of the outer hair cell. Expression levels are indicated by bar charts: blue bars show wild-type expression levels in A; in B and C, red bars indicate upregulation and green bars indicate downregulation.

We suggest that the consistent misregulation of *Ocm*, *Slc26a5*, *Myo3a*, *Sema3e* and *Slc52a3* observed in both the *Mir96^Dmdo^* (Lewis et al., 2016, 2009) and *Mir183/96^dko^* homozygotes is the result of the lack of repression of genes that would usually not be expressed in hair cells at all; for example, *Zeb1*, *Foxo1* and *Nr3c1*. In the wild-type hair cell, miR-96 acts to reinforce hair cell fate by maintaining and/or reinforcing repression of these non-hair cell genes. The links between the direct targets of miR-96 and the consistently misregulated downstream genes have yet to be discovered, but we suggest that *Ikzf2* and *Fos* are likely to be involved (Fig. 7A,B).

There are multiple genes that are known or potential miR-96 targets and are upregulated in miR-96 mutants, but which are known to be expressed in wild-type hair cells, such as *Hspa2*, *St8sia3* and *Grk1*. We suggest that these are examples of genes for which expression in wild-type hair cells is buffered by miR-96, not fully repressed but maintained at a consistent, intermediate level. The loss of this buffering in mutant hair cells results in variable expression and transcriptional noise, not just of these genes (which will all be upregulated to varying degrees) but also of any genes that they regulate. This transcriptional noise is less likely to be consistent between mice but will nonetheless contribute to the degraded functionality of the hair cell (Fig. 7A,B).

Finally, genes that are novel targets of a mutant miRNA have the potential to fulfil both roles. In the case of *Mir96^Dmdo^*, *Ptprq* is downregulated and it is possible that this is due to the mutant miRNA, because *Ptprq* bears a complementary match to the *Mir96^Dmdo^* seed region in its 3′UTR. A reduction in Ptprq in the hair cells will contribute directly to the failure of the hair cells to mature properly. Other novel targets may also be directly affecting the hair cells, or may be adding to the overall transcriptional noise, or both (Fig. 7C).

### The role of *Mir183* in hearing remains unclear

The lack of a *Mir183*-specific mutation means that the effect of miR-183 alone is difficult to ascertain from our data. The relatively low mid-range peak for the miR-183 seed region in our Sylamer analysis of the RNA-seq data (Fig. 5A) implies that the lack of miR-183 has less of a global effect on the transcriptome than the lack of miR-96, and the literature-based regulatory network analysis reflects this; apart from its predicted targets, there are no downstream genes for which misregulation can be attributed to miR-183 alone (Fig. 6A).

### Mice lacking *Mir182* display mild hearing loss with no obvious changes in stereocilia bundles or hair cell innervation

The *Mir182^ko^* heterozygotes have audiograms that resemble those of the wild type, whereas the homozygotes exhibit mild hearing loss at the higher frequencies (Fig. 1). No difference in hair cell stereocilia bundles, innervation or synapse counts was observed between *Mir182^ko^* wild-type, heterozygous and homozygous mice at P28 (Fig. 4; Figs S9 and S10), but, at that age, *Mir182^ko^* homozygotes still have normal hearing. However, even at P56, when *Mir182^ko^* homozygotes exhibit high-frequency hearing loss, the hair cells appear unaffected (Fig. 3B). This is a different phenotype from either the *Mir96^Dmdo^* or the *Mir183/96^dko^* mice and may explain the paucity of significantly misregulated genes in the RNA-seq data from the P4 organ of Corti. This may be too early to see much effect of the absence of miR-182 on the transcriptome. The lack of significantly enriched heptamers corresponding to the miR-182 seed region in the Sylamer analysis for miR-182 also supports this hypothesis (Fig. 5B).

### Relevance to human *MIR96* mutations

In this study, we have shown that the phenotype of mice lacking miR-96 entirely is less severe than that of mice carrying a point mutation in the miR-96 seed region, suggesting that the gain of novel targets plays an important role in the phenotype caused by mutations in miR-96. This has important implications for understanding the effect of mutant miRNAs in the human population. So far, all three reported human mutations in miR-96 have been point mutations, two in the seed region and one in the stem region of the pre-miRNA (Mencia et al., 2009; Solda et al., 2012), and although all the individuals carrying those point mutations have exhibited some degree of progressive hearing loss, the phenotypes differ between people, as do the phenotypes of the mice carrying the *Mir96^Dmdo^* mutation and the *Mir183/96^dko^* allele. Determining the misregulated pathways common to all *Mir96* mutations will be important not only for furthering our understanding of the role of this miRNA in hair cell development but also for developing therapeutic interventions, not only for people with *MIR96* mutations but also more generally for hearing loss associated with hair cell defects.

## MATERIALS AND METHODS

### Ethics approval

Mouse studies were carried out in accordance with UK Home Office regulations and the UK Animals (Scientific Procedures) Act of 1986 (ASPA) under UK Home Office licences, and the study was approved by the Wellcome Trust Sanger Institute and the King's College London Ethical Review Committees. Mice were culled using methods approved under these licences to minimise any possibility of suffering.

### Mice (*Mus musculus*)

The miR-183/96 and miR-182 knockouts in C57BL/6N-derived JM8.A3 ES cells were generated as previously described (Prosser et al., 2011). On chromosome 6, regions 30,169,424 bp to 30,169,772 bp (NCBIM38) for miR-182 and 30,169,424 bp to 30,169,772 bp (NCBIM38) for miR-183/96 were replaced with the *PuroΔtk* selection cassette. For both knockouts, the *PuroΔtk* gene was subsequently deleted by transient transfection with Cre recombinase leading to recombination of the loxP sites that flanked the selection marker under 2-fluoro-2-deoxy-1D-arabinofuranosyl-5-iodouracil (FIAU) selection. Heterozygous targeted ES cells were microinjected into C57BL/6N embryos for chimaera production. Mice were maintained on a C57BL/6N background. Both lines are available through the European Mouse Mutant Archive (*Mir183/96^dko^* mice, EM:10856; *Mir182^ko^* mice, EM:12223). Both males and females were used for all experiments.

### Experimental design

Mutant mice were compared with wild-type littermate controls of the same age and, where possible, sex, although, unless specified, not to the exclusion of mice of the required genotype available. Randomisation is not appropriate for experiments with paired mutant and littermate controls. ABR measurements taken at P14, and samples taken for RNA extraction and immunohistochemistry, were collected prior to genotyping, effectively blinding the collection. No other blinding was carried out. Sample sizes were calculated using the power calculator at sphanalytics.com (https://www.sphanalytics.com/statistical-power-calculator-using-average-values/) along with data from previous experiments of the same kind, with a 5% significance level in all cases. Individual power calculations and exclusion criteria are listed under each relevant method; if no exclusion criteria are listed, all experimental mice were included.

### ABR

The hearing of *Mir183/96^dko^* and *Mir182^ko^* homozygote, heterozygote and wild-type littermates was tested using the ABR, as described previously (Ingham et al., 2011). Animals were sedated using a ketamine/xylazine mix (10 mg ketamine and 0.1 mg xylazine in 0.1 ml/10 g body weight) and recovered using atipamezole (0.01 mg atipamezole in 0.1 ml/10 g body weight) to enable recurrent testing of the same cohort of mice. All injections were intraperitoneal. Responses were recorded from three subcutaneous needle electrodes placed one over the left bulla (reference), one over the right bulla (ground) and one on the vertex (active). We used a broadband click stimulus and 3, 6, 12, 18, 24, 30, 36 and 42 kHz pure tone frequencies, at sound levels from 0 dB to 95 dB, in 5 dB steps. Responses to 256 stimulus presentations were averaged to produce each ABR waveform. The threshold for each stimulus was defined as the lowest intensity at which a waveform could be distinguished visually. Mice were tested at 14 days old (P14), P21, P28±1 day, P56±2 days, P90±2 days and P180±3 days. Any hyperactivity was noted prior to anaesthesia. Wave 1 amplitudes were calculated using ABR Notebook software (courtesy of M. C. Liberman, Harvard Medical School, Boston, MA, USA).

Power calculation: six animals per genotype are required for 99.7% power to detect a meaningful effect size of 15 dB given a standard deviation of 8.45 dB.

Exclusion criteria: if a mouse showed evidence of poor physiological condition (e.g. reduced heartbeat) during ABR recording, recording was stopped, and the data from that session were not included. This is standard procedure and thus pre-established.

### DPOAE measurements

We measured DPOAEs in mice aged 8 weeks old, anaesthetised with an intraperitoneal injection of 0.1 ml/10 g of a solution of 20% urethane. Experiments were performed using Tucker Davis Technologies (TDT) BioSigRZ software driving a TDT RZ6 auditory processor and a pair of TDT MF1 magnetic loudspeakers. Signals were recorded via an Etymotic ER-10B+ low-noise DPOAE microphone. Stimulus tones (f1 and f2) were presented and microphone signals recorded via a closed-field acoustic system sealed into the auditory meatus of the mouse. Stimulus tones were presented at an f2:f1 ratio of 1.2. f2 tones were presented at frequencies to match ABR measurements (6, 12, 18, 24, 30 and 36 kHz). f1 was presented at levels from 0 dB to 85 dB in 5 dB steps. f2 was presented at 10 dB below the level of f1. The magnitude of the 2f1-f2 DPOAE component was extracted from a fast Fourier transform of the recorded microphone signal and plotted as a function of f2 level. For each f2 level, the 20 spectral line magnitudes surrounding the 2f1-f2 frequency were averaged to form a mean noise floor estimate for each measurement. DPOAE threshold was defined as the lowest f2 stimulus level at which the emission magnitude exceeded 2 standard deviations above the mean noise floor.

### Noise exposure

Wild-type and heterozygous *Mir183/96^dko^* mice (P55±1 day) were subjected to an 8-16 kHz octave-band noise at 96 dB SPL for 2 h while awake and unrestrained in separate small cages within an exposure chamber designed to provide a uniform sound field (for chamber details, see Holme and Steel, 2004). Band pass noise was generated digitally using TDT RPvdsEx software, converted to an analogue signal using a TDT RZ6 auditory processor, and amplified using a Brüel and Kjær Type 2716C power amplifier. It was delivered to a compression driver (2446H, JBL, Northridge, CA, USA) connected to a flat front biradial horn (2380A, JBL) secured to the roof of the sound box. ABRs were recorded the day before, and 1, 3, 7, 14 and 28 days after the noise exposure. Unexposed littermates were used as controls and went through the same set of ABR measurements.

### Genotyping

*Mir183/96^dko^* knockout mice were genotyped by PCR analysis using primers spanning the introduced deletion (Table S3). The wild-type band is 841 bp and the mutant band 645 bp. *Mir182^ko^* mice were genotyped in a similar fashion with one of two primer sets (Table S3). For the first set, the wild-type band was 495 bp and the mutant band 457 bp. For the second set, the wild-type band was 247 bp and the mutant band 209 bp.

### Scanning electron microscopy

The inner ears of wild-type, heterozygote and homozygote mice at P28 (*Mir183/96^dko^*, *Mir182^ko^*) and P56 (*Mir182^ko^*) were fixed in 2.5% glutaraldehyde in 0.1 M sodium cacodylate buffer with 3 mM CaCl_2_ at room temperature for 2 h. Cochleae were finely dissected in PBS and processed according to the OTOTO (osmium tetroxide/thiocarbohydrazide/osmium tetroxide/thiocarbohydrazide/osmium tetroxide) method (Hunter-Duvar, 1978). Samples were dehydrated using an ethanol series, critical point dried and mounted for examination. Low-resolution images were taken to identify the 12 kHz region of the cochlea [using the frequency-place map described by Muller et al. 2005)]. Higher-resolution images were taken using a JEOL JSM 7800 Prime scanning electron microscope under a standard magnification (60×) to show the whole organ of Corti, and at higher magnifications to examine hair cell rows (2000×) and individual hair cells (15,000-23,000×). Whole images have been adjusted in Adobe Photoshop to normalise dynamic range across all panels.

Exclusion criteria: if dissection damage was too great to observe any hair cells at the 12 kHz or 24 kHz location in either cochlea, the mouse was not counted. This is standard procedure and thus pre-established.

### Whole-mount immunostaining and confocal microscopy

The cochleae of P28 mice were fixed in 4% paraformaldehyde in PBS, washed in PBS, and decalcified in 0.1 M ethylenediaminetetraacetic acid (EDTA) for 2 h. After fine dissection, samples were blocked in 5% normal horse serum (NHS), 1% bovine serum albumin (BSA) and 0.3% Triton X-100 in PBS for 45 min at room temperature, then immunostained in 1% NHS and 0.3% Triton X-100 in PBS, as described in Buniello et al. (2016). The primary antibodies used were anti-NFH (Abcam, ab4680; 1:800), anti-GluR2 (Millipore, MAB397; 1:200) and anti-Ribeye (Synaptic Systems, 192 103; 1:500), and the secondary antibodies were Alexa Fluor 488 goat anti-chicken (Invitrogen, A11039; 1:300), Alexa Fluor 546 goat anti-rabbit (Invitrogen, A11035; 1:300) and Alexa Fluor 488 goat anti-mouse (Invitrogen, A21131; 1:300). Samples were mounted in ProLong Gold antifade mounting medium with 4′,6-diamidino-2-phenylindole (DAPI) (Life Technologies, P36931), or Vectashield Mounting Medium with DAPI (Vector Laboratories, H-1200), and imaged with a Zeiss Imager 710 confocal microscope (plan-APOCHROMAT 63× oil DIC objective) interfaced with ZEN 2010 software (Carl Zeiss, Germany), or a Nikon A1R point-scanning confocal microscope (Plan Apo VC 60×/1.4NA oil objective) using NIS Elements v4.2 software (Nikon Instruments UK). Confocal *z*-stacks were obtained with a *z*-step size of 0.25 µm (for synapses) or 0.4 µm (for innervation). For synapse counting, two non-overlapping maximum-intensity projection images close to the 12 kHz best-frequency region were collected and synapses were counted using the FIJI plugin of ImageJ, and divided by the number of hair cell nuclei visible in each field of view (between five and 13, depending on which microscope was used) to obtain the number of synapses/hair cell. Where synapses were counted in both ears from the same mouse, the counts were averaged for each mouse before inclusion in the data. Whole images were processed in Adobe Photoshop and adjusted so that all channels were equally visible.

Power calculation for synapse counts: a sample size of four mice (at least one cochlea each) of each genotype and a standard deviation of 2.5 gives 97.7% power to identify a biologically meaningful difference of seven synapses per hair cell. Our actual *Mir183/96^dko^* data (with five wild-type mice and three homozygotes) has a power of 97.2% to detect the effect seen (five synapses per hair cell difference).

Exclusion criteria: if dissection damage was too great to observe any synapses at the 12 kHz or 24 kHz location in either cochlea, the mouse was not counted. This is standard procedure and thus pre-established.

### Dissection and RNA extraction

The organs of Corti of 4-day-old (P4) mice were dissected during a fixed time window (between 6 h and 7.5 h after lights on) to avoid circadian variation, and stored at −20°C in RNAlater stabilisation reagent (Ambion). RNA was extracted from both organs of Corti using either QIAshredder columns (Qiagen, 79654) and the RNeasy mini kit (QIAgen, 74104), or the Lexogen SPLIT kit (Lexogen, 008.48), following the manufacturer's instructions. RNA concentration was measured using a Nanodrop spectrophotometer (ND-8000).

### RNA-seq

RNA from both ears of six wild-type and six sex-matched homozygote mutant littermates from each mutant line were used for RNA-seq. Samples were not pooled. Strand-specific libraries were prepared using the NuGEN Ovation Mouse RNA-Seq System 1-16 kit (NuGEN, 0348) and sequenced on an Illumina HiSeq 2500 machine as paired-end 125 bp reads. The resulting reads were quality checked using FastQC 0.11.4 (https://www.bioinformatics.babraham.ac.uk/projects/fastqc/) and trimmed with Trimmomatic 0.35 (Bolger et al., 2014); adapters were removed, trailing ends were clipped where the quality was low, and a sliding window approach used to control for quality across the entire read. Finally, reads with 36 bp or fewer were discarded, because the shorter a read, the less likely it is to map uniquely to the genome. Reads were assembled to GRCm38 using Hisat2 version 2.0.2beta (Kim et al., 2015). Bam files were soft-clipped beyond end-of-reference alignments and MAPQ scores set to 0 for unmapped reads using Picard 2.1.0 (http://broadinstitute.github.io/picard) and checked for quality using QoRTS (Hartley and Mullikin, 2015). The QoRTS tool also generates count data (in the same format as HTSeq), and these were used with edgeR (Robinson et al., 2010) to carry out a generalised linear model likelihood ratio test. Splicing analyses were performed using Cuffdiff (Cufflinks) (Trapnell et al., 2010), JunctionSeq (Hartley and Mullikin, 2016) and Leafcutter (Li et al., 2018 ).

Power calculations: RNASeqPower was used (Hart et al., 2013). Our depth of sequencing was 83.3 million reads/sample, and we assumed a coefficient of variation of counts of 0.1, which is common for inbred animals (Hart et al., 2013). Given these figures, we calculated that a sample size of six per genotype would result in a 98% chance of detecting a logFC of 0.5 or more.

Exclusion criteria: no RNA-seq data were excluded. During preparation, some samples failed quality control and were replaced.

### Transcriptome and network analysis

Sylamer 18-131 (van Dongen et al., 2008) was used to examine genes ranked in order of their misregulation from up- to downregulated for over- and under-represented heptamers in their 3′ UTRs. IPA (Qiagen, Germany) was used to generate potential upstream regulators for the affected genes, using the causal network analysis module. Upstream regulators that could explain the observed misregulation were identified through the Ingenuity Knowledge Base, a manually curated set of observations from the literature and third-party databases. The activation or inhibition of each upstream regulator was calculated based on the observed misregulation of its target genes. Causal networks were then constructed to link the upstream regulators through a ‘root’ regulator further upstream, again using the Ingenuity Knowledge Base [described in detail in Krämer et al. (2014)]. Causal networks are assessed by their activation *z*-score, which is a measure of the match of observed and predicted misregulation patterns (Krämer et al., 2014). *Z*-scores below −2 are classed as significant by IPA and indicate predicted downregulation or reduced activation of the root regulator, while scores above 2 are also considered significant and indicate predicted upregulation or increased activation of the root regulator. We chose a slightly less stringent cutoff of >1.7 or <–1.7 in order to include upstream regulators predicted to be activated, as would be expected of a miRNA target no longer under repression. The WGCNA R package (Langfelder and Horvath, 2008) was used to carry out weighted gene correlation network analysis. Gene counts from the QoRTS tool were prepared for WGCNA using DESeq2 (Love et al., 2014) and transformed with a variance stabilising transformation. Batch effects were controlled for using the limma R package (Ritchie et al., 2015). Module enrichment analysis was carried out using PANTHER v14 (Mi et al., 2019). Significant enrichment of gene sets was defined as an enrichment score ≥5 and corrected *P*-value (FDR) <0.05. oPOSSUM (Kwon et al., 2012) was used to assess transcription factor binding site overrepresentation in each module. Motif detection in a set of genes can be affected by differing GC composition in the genes used as a ‘background’ set, so background gene sets of 4000-5000 genes were selected for each module such that the GC content matched that of the genes of interest. oPOSSUM assigns two scores to each transcription factor profile, the *z*-score (which assesses whether the rate of occurrence of a given motif in the genes of interest differs significantly from the expected rate calculated from the background genes) and the Fisher score (which compares the proportion of genes of interest which contain a given motif to the proportion of the background genes containing that motif in order to determine the probability of a non-random association between the motif and the genes of interest) (Kwon et al., 2012). We chose to use a threshold of the mean+1 s.d. for each score.

### cDNA creation and qPCR

Organ of Corti RNA was normalised to the same concentration within each litter, then treated with DNAse 1 (Sigma-Aldrich, AMPD1) before cDNA creation. cDNA was made using Superscript II Reverse Transcriptase (Invitrogen, 11904-018) or M-MLV Reverse Transcriptase (Invitrogen, 28025-013) or Precision Reverse Transcription Premix (PrimerDesign, RT-premix2). MicroRNA cDNA was made using the miRCURY LNA RT Kit (Qiagen, 339340). Primers for sequencing cDNA for testing differential splicing were designed using Primer3 (Untergasser et al., 2012) (Table S3). Sanger sequencing was carried out by Source Bioscience and analysed using Gap4 (Bonfield et al., 1995). qPCR was carried out on a CFX Connect qPCR machine (Bio-Rad, USA), using probes from Applied Biosystems and Qiagen (see Table S3 for primer/probe details) and Sso-Advanced Master Mix (Bio-Rad, 1725281) or the miRCURY LNA SYBR Green PCR Kit (Qiagen, 339345) for miRNA qPCR. Relative expression levels were calculated using the 2^−ΔΔct^ equation (Livak and Schmittgen, 2001), with *Hprt* as an internal control for all protein-coding genes except *Ocm* and *Slc26a5*, which are specifically expressed in hair cells, for which *Jag1* was used as an internal control, because it is expressed in supporting cells of the organ of Corti (Morrison et al., 1999; Zine et al., 2000). For all other genes and miRNAs, the quantity of sensory tissue present was checked using *Jag1*, and pairs were only used if their *Jag1* levels did not differ by more than ±20%. For the miRNA qPCR, the internal control was *Mir99a*, which is expressed in most cell types in the cochlea (Friedman et al., 2009). At least three technical replicates of each sample were carried out for each reaction, and at least four biological replicates were tested per probe (see legends of Figs S1 and S11 for numbers for each probe).

Power calculations: we estimated the power to detect a difference of 40% for a sample size of four wild types and four homozygotes, with a standard deviation of 0.01 for wild types and 0.2 for homozygotes, which is based on previous data (the discrepancy in standard deviation is the result of the 2^−ΔΔct^ calculation of relative expression levels). The power is 97.9%.

Exclusion criteria: data from wild-type/homozygote pairs in which the *Jag1* levels differed by more than 20% were not included. This is a pre-established criterion based on our previous work.

### Statistics

For qPCR data, the Wilcoxon rank sum test (Mann–Whitney *U-*test) was chosen to determine significance, because it is a suitable test for small sample sizes and populations of unknown characteristics (Bridge and Sawilowsky, 1999). For the miRNA qPCR and synapse count data, we used a one-way ANOVA because three groups were being compared. Post-hoc test *P*-values were adjusted using the Bonferroni correction. For repeated ABR threshold analyses, the thresholds were not normally distributed, so the data were first transformed using the arcsine transformation then analysed using separate linear models for each frequency with a compound symmetric covariance structure and restricted Maximum Likelihood Estimation (Duricki et al., 2016). This allowed the inclusion of all available data, unlike the repeated measures ANOVA, which requires the discarding of a subject if any data points are missed (for example, if a mouse died before the final ABR measurement) (Krueger and Tian, 2004). For each stimulus, the double interaction of genotype and age was measured, followed by Bonferroni correction for multiple testing. Wilcoxon rank sum tests were carried out using R, and the one-way ANOVAs, arcsine transformation and mixed model linear pairwise comparison were done with SPSS v25 (IBM SPSS Statistics, IBM, USA).

### Immunohistochemistry

Samples from P4 pups were collected, fixed in 10% formalin, embedded in paraffin wax and cut into 8 μm sections. Immunohistochemistry was carried out using a Ventana Discovery machine and reagents according to the manufacturer's instructions [DABMap™ Kit (760-124), Haematoxylin (760-2021), Bluing reagent (760-2037), CC1 (950-124), EZPrep (950-100), LCS (650-010), RiboWash (760-105), Reaction Buffer (95-300), and RiboCC (760-107)]. For each antibody, at least three wild-type/homozygote littermate pairs were tested, and from each animal, at least five mid-modiolar sections were used per antibody. Primary antibodies used were rabbit anti-Ocm (Abcam, ab150947; 1:50) and goat anti-Prestin (*Slc26a5*) (Santa Cruz Biotechnology, sc-22692; 1:50), and the secondary antibodies were anti-goat (Jackson ImmunoResearch, 705-065-147; 1:100), and anti-rabbit (Jackson ImmunoResearch, 711-065-152; 1:100). The expression of Ocm and Prestin in outer hair cells has been previously described (Liberman et al., 2002; Sakaguchi et al., 1998), and these antibodies have previously been used for mouse outer hair cells (Lewis et al., 2009; Tang et al., 2019; Zhang et al., 2016). Antibodies were diluted in staining solution (10% foetal calf serum, 0.1% Triton X-100, 2% BSA and 0.5% sodium azide in PBS). A Zeiss Axioskop 2 microscope with a Plan Neofluar 63×1.4 NA objective was used to examine slides, and photos were taken using a Zeiss Axiocam camera and the associated Axiocam software (Carl Zeiss, Germany). Images were processed in Adobe Photoshop; minimal adjustments were made, including rotation and resizing. Where image settings were altered, the adjustment was applied equally to wild-type and mutant photos and to the whole image.

### Prediction of potential causal regulatory networks

In our previous analysis (Lewis et al., 2016), we used interactions from the literature to connect miR-96 to as many of the misregulated genes in *Mir96^Dmdo^* homozygotes as possible. In order to automate this procedure, a custom perl script [prediction of potential causal regulatory networks (PoPCoRN)] was written to make use of publicly available regulatory data from ArrayExpress and the associated Expression Atlas (Athar et al., 2019; Petryszak et al., 2016), ORegAnno (Lesurf et al., 2016), miRTarBase (Chou et al., 2018), TransmiR (Tong et al., 2019) and TRRUST (Han et al., 2018) (Table S9). All these data are based on experimental evidence, although we did make use of human regulatory interactions by converting human gene IDs to mouse gene IDs where there was a one-to-one orthologue (using Ensembl). Interactions from our previous network (Lewis et al., 2016), which were obtained from the literature using Ingenuity IPA, were also added, as were regulations reported in Hertzano et al. (2007), which is not available through ArrayExpress but is a particularly relevant study, because it reports the results of microarrays carried out on RNA from mouse inner ears. MicroRNA targets confirmed in the literature were included, as were experimentally validated targets listed in miRTarBase as having ‘strong experimental evidence’, either reporter assay or western blotting (Chou et al., 2018), and genes predicted or confirmed to be miR-96 targets by our previous studies (Lewis et al., 2016, 2009). Finally, genes upregulated in *Mir183/96^dko^* homozygotes with heptamers complementary to either the miR-96 or the miR-183 seed region in their 3′UTR were included as targets of the relevant miRNA(s) (Table 1A). Similarly, genes upregulated in *Mir182^ko^* homozygotes with heptamers complementary to the miR-182 seed region in their 3′UTR were included as targets of miR-182 (Table 1B). This resulted in a list of 97,062 unique links of the form <gene A> <interaction> <gene B>.

All potential links between the ultimate regulators (*Mir183* and *Mir96*, *Mir96* alone, or *Mir182*) and the misregulated genes were then identified, and the direction of misregulation of intermediate genes predicted. Starting with the known misregulated genes, each upstream regulator was given a score based on the direction of regulation of the known misregulated genes (Fig. S19). This process iterated until the gene(s) at the top of the cascade (in this case, *Mir96*, *Mir183* or *Mir182*) were reached. Consistent links were kept, the shortest paths between miRNA and misregulated genes identified, and the final network was written out in the simple interaction format (sif) and viewed using Cytoscape (Shannon et al., 2003) (Fig. S18). Further network analyses were carried out using the Cytoscape Network Analyser tool to calculate the degree and betweenness centrality of each node (gene). A node's degree is the number of edges connecting it to other nodes (in this case, interactions between genes and gene products), and its betweenness centrality measures how important it is for connecting distant parts of the network.

In order to test the PoPCoRN tool, we searched for studies that measured the misregulation of a set of genes in a system in which a specific upstream regulator was either induced or silenced, and that also identified and confirmed the misregulation of an intermediate gene (where necessary, the data for these intermediate genes were removed from the input prior to network creation). We found ten suitable studies from which we were able to create networks and test the predicted misregulation of 14 intermediate genes. The tool made correct predictions for seven of the genes and incorrect predictions for three of them (Table S10). For the remaining four, it did not predict either up- or downregulation, which points to a deficiency of underlying data (Table S10). This will always be a problem for a tool based on existing data that does not attempt extrapolation.

Many similar approaches to network creation have been described previously (for example, Chindelevitch et al., 2012; Fakhry et al., 2016; Krämer et al., 2014; Pollard et al., 2005), but they address the case in which the upstream regulator or regulators remain unknown; for example, when comparing samples from healthy and diseased tissues, and one of their main aims is to identify potential upstream regulators. Our implementation asks a different question, exploring the downstream regulatory cascade of a known regulator. In the case of miR-96, and potentially many other regulators involved in disease, the genes involved in mediating their effect are candidate therapeutic targets.

### Strain differences in seed region matches

The C57BL/6NJ genomic sequence is available from the Mouse Genomes Project (Adams et al., 2015) via the Ensembl browser (Zerbino et al., 2018), but the *Mir96^Dmdo^* mice were made and maintained on the C3HeB/FeJ background, which is not one of the sequenced strains. Instead, we made use of genomic sequence from the C3H/HeJ strain, which is closely related to C3HeB/FeJ and is one of the other strains sequenced as part of the Mouse Genomes Project. We searched the 3′UTRs of each strain for the complement of the miR-96 seed region (GTGCCAA). We removed genes without an MGI ID, because without a consistent ID the corresponding gene in the other strain could not be identified. We found that 1733 genes were left with at least one match to the miR-96 seed region in their 3′UTRs in one or both strains, 21 genes had no 3′UTR sequence available for C57BL/6NJ, and 31 genes had no 3′UTR sequence available for C3H/HeJ.

## Supplementary Material

Supplementary information

## References

[DMM047225C1] Adams, D. J., Doran, A. G., Lilue, J. and Keane, T. M. (2015). The mouse genomes project: a repository of inbred laboratory mouse strain genomes. *Mamm. Genome* 26, 403-412. 10.1007/s00335-015-9579-6 26123534

[DMM047225C2] Agerman, K., Hjerling-Leffler, J., Blanchard, M. P., Scarfone, E., Canlon, B., Nosrat, C. and Ernfors, P. (2003). BDNF gene replacement reveals multiple mechanisms for establishing neurotrophin specificity during sensory nervous system development. *Development* 130, 1479-1491. 10.1242/dev.00378 12620975

[DMM047225C3] Ashburner, M., Ball, C. A., Blake, J. A., Botstein, D., Butler, H., Cherry, J. M., Davis, A. P., Dolinski, K., Dwight, S. S., Eppig, J. T. et al. (2000). Gene ontology: tool for the unification of biology. The gene ontology consortium. *Nat. Genet.* 25, 25-29. 10.1038/75556 10802651 PMC3037419

[DMM047225C4] Athar, A., Fullgrabe, A., George, N., Iqbal, H., Huerta, L., Ali, A., Snow, C., Fonseca, N. A., Petryszak, R., Papatheodorou, I. et al. (2019). ArrayExpress update - from bulk to single-cell expression data. *Nucleic Acids Res.* 47, D711-D715. 10.1093/nar/gky964 30357387 PMC6323929

[DMM047225C5] Barreau, C., Paillard, L. and Osborne, H. B. (2005). AU-rich elements and associated factors: are there unifying principles? *Nucleic Acids Res.* 33, 7138-7150. 10.1093/nar/gki1012 16391004 PMC1325018

[DMM047225C6] Bolger, A. M., Lohse, M. and Usadel, B. (2014). Trimmomatic: a flexible trimmer for Illumina sequence data. *Bioinformatics* 30, 2114-2120. 10.1093/bioinformatics/btu170 24695404 PMC4103590

[DMM047225C7] Bonfield, J. K., Smith, K. and Staden, R. (1995). A new DNA sequence assembly program. *Nucleic Acids Res.* 23, 4992-4999. 10.1093/nar/23.24.4992 8559656 PMC307504

[DMM047225C8] Boutz, P. L., Chawla, G., Stoilov, P. and Black, D. L. (2007). MicroRNAs regulate the expression of the alternative splicing factor nPTB during muscle development. *Genes Dev.* 21, 71-84. 10.1101/gad.1500707 17210790 PMC1759902

[DMM047225C9] Bridge, P. D. and Sawilowsky, S. S. (1999). Increasing physicians’ awareness of the impact of statistics on research outcomes: comparative power of the *t*-test and and Wilcoxon Rank-Sum test in small samples applied research. *J. Clin. Epidemiol.* 52, 229-235. 10.1016/S0895-4356(98)00168-1 10210240

[DMM047225C10] Buniello, A., Ingham, N. J., Lewis, M. A., Huma, A. C., Martinez-Vega, R., Varela-Nieto, I., Vizcay-Barrena, G., Fleck, R. A., Houston, O., Bardhan, T. et al. (2016). Wbp2 is required for normal glutamatergic synapses in the cochlea and is crucial for hearing. *EMBO Mol. Med.* 8, 191-207. 10.15252/emmm.201505523 26881968 PMC4772953

[DMM047225C11] Cai, T., Jen, H.-I., Kang, H., Klisch, T. J., Zoghbi, H. Y. and Groves, A. K. (2015). Characterization of the transcriptome of nascent hair cells and identification of direct targets of the Atoh1 transcription factor. *J. Neurosci.* 35, 5870-5883. 10.1523/JNEUROSCI.5083-14.2015 25855195 PMC4388939

[DMM047225C12] Chen, C.-Y. and Shyu, A.-B. (1995). AU-rich elements: characterization and importance in mRNA degradation. *Trends Biochem. Sci.* 20, 465-470. 10.1016/S0968-0004(00)89102-1 8578590

[DMM047225C13] Chen, J., Johnson, S. L., Lewis, M. A., Hilton, J. M., Huma, A., Marcotti, W. and Steel, K. P. (2014). A reduction in Ptprq associated with specific features of the deafness phenotype of the miR-96 mutant mouse diminuendo. *Eur. J. Neurosci.* 39, 744-756. 10.1111/ejn.12484 24446963 PMC4065360

[DMM047225C14] Chessum, L., Matern, M. S., Kelly, M. C., Johnson, S. L., Ogawa, Y., Milon, B., Mcmurray, M., Driver, E. C., Parker, A., Song, Y. et al. (2018). Helios is a key transcriptional regulator of outer hair cell maturation. *Nature* 563, 696-700. 10.1038/s41586-018-0728-4 30464345 PMC6542691

[DMM047225C15] Chindelevitch, L., Ziemek, D., Enayetallah, A., Randhawa, R., Sidders, B., Brockel, C. and Huang, E. S. (2012). Causal reasoning on biological networks: interpreting transcriptional changes. *Bioinformatics* 28, 1114-1121. 10.1093/bioinformatics/bts090 22355083

[DMM047225C16] Chou, C.-H., Shrestha, S., Yang, C.-D., Chang, N.-W., Lin, Y.-L., Liao, K.-W., Huang, W.-C., Sun, T.-H., Tu, S.-J., Lee, W.-H. et al. (2018). miRTarBase update 2018: a resource for experimentally validated microRNA-target interactions. *Nucleic Acids Res.* 46, D296-D302. 10.1093/nar/gkx1067 29126174 PMC5753222

[DMM047225C17] Cui, H. and Yang, L. (2013). Analysis of microRNA expression detected by microarray of the cerebral cortex after hypoxic-ischemic brain injury. *J. Craniofac. Surg.* 24, 2147-2152. 10.1097/SCS.0b013e3182a243f3 24220425

[DMM047225C18] Daamen, W. F. and Quaglino, D. (2019). Signaling pathways in elastic tissues. *Cell. Signal.* 63, 109364 10.1016/j.cellsig.2019.109364 31351217

[DMM047225C19] Demambro, V. E., Kawai, M., Clemens, T. L., Fulzele, K., Maynard, J. A., Marin De Evsikova, C., Johnson, K. R., Canalis, E., Beamer, W. G., Rosen, C. J. et al. (2010). A novel spontaneous mutation of Irs1 in mice results in hyperinsulinemia, reduced growth, low bone mass and impaired adipogenesis. *J. Endocrinol.* 204, 241-253. 10.1677/JOE-09-0328 20032200 PMC3033737

[DMM047225C20] Deol, M. S. (1970). The origin of the acoustic ganglion and effects of the gene dominant spotting (Wv) in the mouse. *J. Embryol. Exp. Morphol.* 23, 773-784. 5473311

[DMM047225C21] Dickinson, M. E., Flenniken, A. M., Ji, X., Teboul, L., Wong, M. D., White, J. K., Meehan, T. F., Weninger, W. J., Westerberg, H., Adissu, H. et al. (2016). High-throughput discovery of novel developmental phenotypes. *Nature* 537, 508-514. 10.1038/nature19356 27626380 PMC5295821

[DMM047225C22] Duan, X., Gan, J., Peng, D. Y., Bao, Q., Xiao, L., Wei, L. and Wu, J. (2019). Identification and functional analysis of microRNAs in rats following focal cerebral ischemia injury. *Mol. Med. Rep.* 19, 4175-4184. 10.3892/mmr.2019.10073 30896823 PMC6471137

[DMM047225C23] Duricki, D. A., Soleman, S. and Moon, L. D. (2016). Analysis of longitudinal data from animals with missing values using SPSS. *Nat. Protoc.* 11, 1112-1129. 10.1038/nprot.2016.048 27196723 PMC5582138

[DMM047225C24] Elkon, R., Milon, B., Morrison, L., Shah, M., Vijayakumar, S., Racherla, M., Leitch, C. C., Silipino, L., Hadi, S., Weiss-Gayet, M. et al. (2015). RFX transcription factors are essential for hearing in mice. *Nat. Commun.* 6, 8549 10.1038/ncomms9549 26469318 PMC4634137

[DMM047225C25] Erichsen, S., Stierna, P., Bagger-Sjoback, D., Curtis, L. M., Rarey, K. E., Schmid, W. and Hultcrantz, M. (1998). Distribution of Na,K-ATPase is normal in the inner ear of a mouse with a null mutation of the glucocorticoid receptor. *Hear. Res.* 124, 146-154. 10.1016/S0378-5955(98)00117-8 9822912

[DMM047225C26] Fabregat, A., Jupe, S., Matthews, L., Sidiropoulos, K., Gillespie, M., Garapati, P., Haw, R., Jassal, B., Korninger, F., May, B. et al. (2018). The reactome pathway knowledgebase. *Nucleic Acids Res.* 46, D649-D655. 10.1093/nar/gkx1132 29145629 PMC5753187

[DMM047225C27] Fakhry, C. T., Choudhary, P., Gutteridge, A., Sidders, B., Chen, P., Ziemek, D. and Zarringhalam, K. (2016). Interpreting transcriptional changes using causal graphs: new methods and their practical utility on public networks. *BMC Bioinformatics* 17, 318 10.1186/s12859-016-1181-8 27553489 PMC4995651

[DMM047225C28] Fan, J., Jia, L., Li, Y., Ebrahim, S., May-Simera, H., Wood, A., Morell, R. J., Liu, P., Lei, J., Kachar, B. et al. (2017). Maturation arrest in early postnatal sensory receptors by deletion of the miR-183/96/182 cluster in mouse. *Proc. Natl. Acad. Sci. USA* 114, E4271-E4280. 10.1073/pnas.1619442114 28484004 PMC5448201

[DMM047225C29] Friedman, L. M., Dror, A. A., Mor, E., Tenne, T., Toren, G., Satoh, T., Biesemeier, D. J., Shomron, N., Fekete, D. M., Hornstein, E. et al. (2009). MicroRNAs are essential for development and function of inner ear hair cells in vertebrates. *Proc. Natl. Acad. Sci. USA* 106, 7915-7920. 10.1073/pnas.0812446106 19416898 PMC2683084

[DMM047225C30] Geng, R., Furness, D. N., Muraleedharan, C. K., Zhang, J., Dabdoub, A., Lin, V. and Xu, S. (2018). The microRNA-183/96/182 cluster is essential for stereociliary bundle formation and function of cochlear sensory hair cells. *Sci. Rep.* 8, 18022 10.1038/s41598-018-36894-z 30575790 PMC6303392

[DMM047225C31] Gilels, F., Paquette, S. T., Zhang, J., Rahman, I. and White, P. M. (2013). Mutation of Foxo3 causes adult onset auditory neuropathy and alters cochlear synapse architecture in mice. *J. Neurosci.* 33, 18409-18424. 10.1523/JNEUROSCI.2529-13.2013 24259566 PMC6618809

[DMM047225C32] Goodyear, R. J., Legan, P. K., Wright, M. B., Marcotti, W., Oganesian, A., Coats, S. A., Booth, C. J., Kros, C. J., Seifert, R. A., Bowen-Pope, D. F. et al. (2003). A receptor-like inositol lipid phosphatase is required for the maturation of developing cochlear hair bundles. *J. Neurosci.* 23, 9208-9219. 10.1523/JNEUROSCI.23-27-09208.2003 14534255 PMC6740823

[DMM047225C33] Han, H., Cho, J. W., Lee, S., Yun, A., Kim, H., Bae, D., Yang, S., Kim, C. Y., Lee, M., Kim, E. et al. (2018). TRRUST v2: an expanded reference database of human and mouse transcriptional regulatory interactions. *Nucleic Acids Res.* 46, D380-D386. 10.1093/nar/gkx1013 29087512 PMC5753191

[DMM047225C34] Hart, S. N., Therneau, T. M., Zhang, Y., Poland, G. A. and Kocher, J. P. (2013). Calculating sample size estimates for RNA sequencing data. *J. Comput. Biol.* 20, 970-978. 10.1089/cmb.2012.0283 23961961 PMC3842884

[DMM047225C35] Hartley, S. W. and Mullikin, J. C. (2015). QoRTs: a comprehensive toolset for quality control and data processing of RNA-Seq experiments. *BMC Bioinformatics* 16, 224 10.1186/s12859-015-0670-5 26187896 PMC4506620

[DMM047225C36] Hartley, S. W. and Mullikin, J. C. (2016). Detection and visualization of differential splicing in RNA-Seq data with JunctionSeq. *Nucleic Acids Res.* 44, e127 10.1093/nar/gkw501 27257077 PMC5009739

[DMM047225C37] Hertzano, R., Dror, A. A., Montcouquiol, M., Ahmed, Z. M., Ellsworth, B., Camper, S., Friedman, T. B., Kelley, M. W. and Avraham, K. B. (2007). Lhx3, a LIM domain transcription factor, is regulated by Pou4f3 in the auditory but not in the vestibular system. *Eur. J. Neurosci.* 25, 999-1005. 10.1111/j.1460-9568.2007.05332.x 17331196

[DMM047225C38] Hertzano, R., Elkon, R., Kurima, K., Morrisson, A., Chan, S. L., Sallin, M., Biedlingmaier, A., Darling, D. S., Griffith, A. J., Eisenman, D. J. et al. (2011). Cell type-specific transcriptome analysis reveals a major role for Zeb1 and miR-200b in mouse inner ear morphogenesis. *PLoS Genet.* 7, e1002309 10.1371/journal.pgen.1002309 21980309 PMC3183091

[DMM047225C39] Holme, R. H. and Steel, K. P. (2004). Progressive hearing loss and increased susceptibility to noise-induced hearing loss in mice carrying a Cdh23 but not a Myo7a mutation. *J. Assoc. Res. Otolaryngol.* 5, 66-79. 10.1007/s10162-003-4021-2 14648237 PMC2538366

[DMM047225C40] Hornstein, E. and Shomron, N. (2006). Canalization of development by microRNAs. *Nat. Genet.* 38 Suppl. S6, S20-S24. 10.1038/ng1803 16736020

[DMM047225C41] Hulander, M., Wurst, W., Carlsson, P. and Enerback, S. (1998). The winged helix transcription factor Fkh10 is required for normal development of the inner ear. *Nat. Genet.* 20, 374-376. 10.1038/3850 9843211

[DMM047225C42] Hunter-Duvar, I. M. (1978). A technique for preparation of cochlear specimens for assessment with the scanning electron microscope. *Acto. Otoloaryng. Suppl.* 351, 3-23. 10.3109/00016487809122718 352089

[DMM047225C43] Ingham, N. J., Pearson, S. and Steel, K. P. (2011). Using the auditory brainstem response (ABR) to determine sensitivity of hearing in mutant mice. *Curr. Protoc. Mouse Biol.* 1, 279-287. 10.1002/9780470942390.mo110059 26069055

[DMM047225C44] Karnik, S. K., Brooke, B. S., Bayes-Genis, A., Sorensen, L., Wythe, J. D., Schwartz, R. S., Keating, M. T. and Li, D. Y. (2003). A critical role for elastin signaling in vascular morphogenesis and disease. *Development* 130, 411-423. 10.1242/dev.00223 12466207

[DMM047225C45] Kim, D., Langmead, B. and Salzberg, S. L. (2015). HISAT: a fast spliced aligner with low memory requirements. *Nat. Methods* 12, 357-360. 10.1038/nmeth.3317 25751142 PMC4655817

[DMM047225C46] Krämer, A., Green, J., Pollard, J., Jr and Tugendreich, S. (2014). Causal analysis approaches in Ingenuity Pathway Analysis. *Bioinformatics* 30, 523-530. 10.1093/bioinformatics/btt703 24336805 PMC3928520

[DMM047225C47] Krueger, C. and Tian, L. (2004). A comparison of the general linear mixed model and repeated measures ANOVA using a dataset with multiple missing data points. *Biol. Res. Nurs.* 6, 151-157. 10.1177/1099800404267682 15388912

[DMM047225C48] Kuhn, S., Johnson, S. L., Furness, D. N., Chen, J., Ingham, N., Hilton, J. M., Steffes, G., Lewis, M. A., Zampini, V., Hackney, C. M. et al. (2011). miR-96 regulates the progression of differentiation in mammalian cochlear inner and outer hair cells. *Proc. Natl. Acad. Sci. USA* 108, 2355-2360. 10.1073/pnas.1016646108 21245307 PMC3038748

[DMM047225C49] Kujawa, S. G. and Liberman, M. C. (2009). Adding insult to injury: cochlear nerve degeneration after “temporary” noise-induced hearing loss. *J. Neurosci.* 29, 14077-14085. 10.1523/JNEUROSCI.2845-09.2009 19906956 PMC2812055

[DMM047225C50] Kurima, K., Peters, L. M., Yang, Y., Riazuddin, S., Ahmed, Z. M., Naz, S., Arnaud, D., Drury, S., Mo, J., Makishima, T. et al. (2002). Dominant and recessive deafness caused by mutations of a novel gene, TMC1, required for cochlear hair-cell function. *Nat. Genet.* 30, 277-284. 10.1038/ng842 11850618

[DMM047225C51] Kwon, A. T., Arenillas, D. J., Worsley Hunt, R. and Wasserman, W. W. (2012). oPOSSUM-3: advanced analysis of regulatory motif over-representation across genes or ChIP-Seq datasets. *G3 (Bethesda)* 2, 987-1002. 10.1534/g3.112.003202 22973536 PMC3429929

[DMM047225C52] Lalani, S. R., Safiullah, A. M., Molinari, L. M., Fernbach, S. D., Martin, D. M. and Belmont, J. W. (2004). SEMA3E mutation in a patient with CHARGE syndrome. *J. Med. Genet.* 41, e94 10.1136/jmg.2003.017640 15235037 PMC1735828

[DMM047225C53] Lang, H., Schulte, B. A., Zhou, D., Smythe, N., Spicer, S. S. and Schmiedt, R. A. (2006). Nuclear factor kappaB deficiency is associated with auditory nerve degeneration and increased noise-induced hearing loss. *J. Neurosci.* 26, 3541-3550. 10.1523/JNEUROSCI.2488-05.2006 16571762 PMC2897814

[DMM047225C54] Langfelder, P. and Horvath, S. (2008). WGCNA: an R package for weighted correlation network analysis. *BMC Bioinformatics* 9, 559 10.1186/1471-2105-9-559 19114008 PMC2631488

[DMM047225C55] Lesurf, R., Cotto, K. C., Wang, G., Griffith, M., Kasaian, K., Jones, S. J., Montgomery, S. B. and Griffith, O. L. (2016). ORegAnno 3.0: a community-driven resource for curated regulatory annotation. *Nucleic Acids Res.* 44, D126-D132. 10.1093/nar/gkv1203 26578589 PMC4702855

[DMM047225C56] Lewis, M. A., Quint, E., Glazier, A. M., Fuchs, H., De Angelis, M. H., Langford, C., Van Dongen, S., Abreu-Goodger, C., Piipari, M., Redshaw, N. et al. (2009). An ENU-induced mutation of miR-96 associated with progressive hearing loss in mice. *Nat. Genet.* 41, 614-618. 10.1038/ng.369 19363478 PMC2705913

[DMM047225C57] Lewis, M. A., Buniello, A., Hilton, J. M., Zhu, F., Zhang, W. I., Evans, S., Van Dongen, S., Enright, A. J. and Steel, K. P. (2016). Exploring regulatory networks of miR-96 in the developing inner ear. *Sci. Rep.* 6, 23363 10.1038/srep23363 26988146 PMC4796898

[DMM047225C58] Li, H. S. and Borg, E. (1991). Age-related loss of auditory sensitivity in two mouse genotypes. *Acta Otolaryngol.* 111, 827-834. 10.3109/00016489109138418 1759567

[DMM047225C59] Li, H., Gong, Y., Qian, H., Chen, T., Liu, Z., Jiang, Z. and Wei, S. (2015). Brain-derived neurotrophic factor is a novel target gene of the has-miR-183/96/182 cluster in retinal pigment epithelial cells following visible light exposure. *Mol. Med. Rep.* 12, 2793-2799. 10.3892/mmr.2015.3736 25955435

[DMM047225C61] Li, Y. I., Knowles, D. A., Humphrey, J., Barbeira, A. N., Dickinson, S. P., Im, H. K. and Pritchard, J. K. (2018). Annotation-free quantification of RNA splicing using LeafCutter. *Nat. Genet.* 50, 151-158. 10.1038/s41588-017-0004-9 29229983 PMC5742080

[DMM047225C62] Liberman, M. C., Gao, J., He, D. Z., Wu, X., Jia, S. and Zuo, J. (2002). Prestin is required for electromotility of the outer hair cell and for the cochlear amplifier. *Nature* 419, 300-304. 10.1038/nature01059 12239568

[DMM047225C63] Ling, W., Xu, X. and Liu, J. (2017). A causal relationship between the neurotherapeutic effects of miR182/7a and decreased expression of PRDM5. *Biochem. Biophys. Res. Commun.* 490, 1-7. 10.1016/j.bbrc.2017.05.141 28552531

[DMM047225C64] Liu, X. Z., Ouyang, X. M., Xia, X. J., Zheng, J., Pandya, A., Li, F., Du, L. L., Welch, K. O., Petit, C., Smith, R. J. et al. (2003). Prestin, a cochlear motor protein, is defective in non-syndromic hearing loss. *Hum. Mol. Genet.* 12, 1155-1162. 10.1093/hmg/ddg127 12719379

[DMM047225C65] Liu, Y., Bailey, J. C., Helwa, I., Dismuke, W. M., Cai, J., Drewry, M., Brilliant, M. H., Budenz, D. L., Christen, W. G., Chasman, D. I. et al. (2016). A common variant in MIR182 is associated with primary open-angle glaucoma in the NEIGHBORHOOD consortium. *Invest. Ophthalmol. Vis. Sci.* 57, 4528-4535. 10.1167/iovs.16-19688 27537254 PMC4991020

[DMM047225C66] Livak, K. J. and Schmittgen, T. D. (2001). Analysis of relative gene expression data using real-time quantitative PCR and the 2−ΔΔCT method. *Methods* 25, 402-408. 10.1006/meth.2001.1262 11846609

[DMM047225C67] Love, M. I., Huber, W. and Anders, S. (2014). Moderated estimation of fold change and dispersion for RNA-seq data with DESeq2. *Genome Biol.* 15, 550 10.1186/s13059-014-0550-8 25516281 PMC4302049

[DMM047225C68] Lumayag, S., Haldin, C. E., Corbett, N. J., Wahlin, K. J., Cowan, C., Turturro, S., Larsen, P. E., Kovacs, B., Witmer, P. D., Valle, D. et al. (2013). Inactivation of the microRNA-183/96/182 cluster results in syndromic retinal degeneration. *Proc. Natl. Acad. Sci. USA* 110, E507-E516. 10.1073/pnas.1212655110 23341629 PMC3568372

[DMM047225C69] Mencia, A., Modamio-Høybjor, S., Redshaw, N., Morín, M., Mayo-Merino, F., Olavarrieta, L., Aguirre, L. A., del Castillo, I., Steel, K. P., Dalmay, T. et al. (2009). Mutations in the seed region of human miR-96 are responsible for nonsyndromic progressive hearing loss. *Nat. Genet.* 41, 609-613. 10.1038/ng.355 19363479

[DMM047225C70] Mi, H., Muruganujan, A., Ebert, D., Huang, X. and Thomas, P. D. (2019). PANTHER version 14: more genomes, a new PANTHER GO-slim and improvements in enrichment analysis tools. *Nucleic Acids Res.* 47, D419-D426. 10.1093/nar/gky1038 30407594 PMC6323939

[DMM047225C71] Moore, B. A., Roux, M. J., Sebbag, L., Cooper, A., Edwards, S. G., Leonard, B. C., Imai, D. M., Griffey, S., Bower, L., Clary, D. et al. (2018). A population study of common ocular abnormalities in C57BL/6N rd8 mice. *Invest. Ophthalmol. Vis. Sci.* 59, 2252-2261. 10.1167/iovs.17-23513 29847629 PMC5935295

[DMM047225C72] Morrison, A., Hodgetts, C., Gossler, A., Hrabe De Angelis, M. and Lewis, J. (1999). Expression of Delta1 and Serrate1 (Jagged1) in the mouse inner ear. *Mech. Dev.* 84, 169-172. 10.1016/S0925-4773(99)00066-0 10473135

[DMM047225C73] Muller, M., Von Hunerbein, K., Hoidis, S. and Smolders, J. W. (2005). A physiological place-frequency map of the cochlea in the CBA/J mouse. *Hear. Res.* 202, 63-73. 10.1016/j.heares.2004.08.011 15811700

[DMM047225C74] Nakano, Y., Jahan, I., Bonde, G., Sun, X., Hildebrand, M. S., Engelhardt, J. F., Smith, R. J., Cornell, R. A., Fritzsch, B. and Banfi, B. (2012). A mutation in the Srrm4 gene causes alternative splicing defects and deafness in the Bronx waltzer mouse. *PLoS Genet.* 8, e1002966 10.1371/journal.pgen.1002966 23055939 PMC3464207

[DMM047225C75] Nakano, Y., Kelly, M. C., Rehman, A. U., Boger, E. T., Morell, R. J., Kelley, M. W., Friedman, T. B. and Banfi, B. (2018). Defects in the alternative splicing-dependent regulation of REST cause deafness. *Cell* 174, 536-548.e21. 10.1016/j.cell.2018.06.004 29961578 PMC6370011

[DMM047225C76] Noben-Trauth, K., Zheng, Q. Y. and Johnson, K. R. (2003). Association of cadherin 23 with polygenic inheritance and genetic modification of sensorineural hearing loss. *Nat. Genet.* 35, 21-23. 10.1038/ng1226 PMC2864026 12910270

[DMM047225C77] Oishi, N., Chen, J., Zheng, H.-W., Hill, K., Schacht, J. and Sha, S.-H. (2013). Tumor necrosis factor-alpha-mutant mice exhibit high frequency hearing loss. *J. Assoc. Res. Otolaryngol.* 14, 801-811. 10.1007/s10162-013-0410-3 23996384 PMC3825018

[DMM047225C78] Paylor, R., Johnson, R. S., Papaioannou, V., Spiegelman, B. M. and Wehner, J. M. (1994). Behavioral assessment of c-fos mutant mice. *Brain Res.* 651, 275-282. 10.1016/0006-8993(94)90707-2 7922576

[DMM047225C79] Petryszak, R., Keays, M., Tang, Y. A., Fonseca, N. A., Barrera, E., Burdett, T., Fullgrabe, A., Fuentes, A. M., Jupp, S., Koskinen, S. et al. (2016). Expression Atlas update--an integrated database of gene and protein expression in humans, animals and plants. *Nucleic Acids Res.* 44, D746-D752. 10.1093/nar/gkv1045 26481351 PMC4702781

[DMM047225C80] Pittler, S. J. and Baehr, W. (1991). Identification of a nonsense mutation in the rod photoreceptor cGMP phosphodiesterase beta-subunit gene of the rd mouse. *Proc. Natl. Acad. Sci. USA* 88, 8322-8326. 10.1073/pnas.88.19.8322 1656438 PMC52500

[DMM047225C81] Plass, M., Rasmussen, S. H. and Krogh, A. (2017). Highly accessible AU-rich regions in 3′ untranslated regions are hotspots for binding of regulatory factors. *PLoS Comput. Biol.* 13, e1005460 10.1371/journal.pcbi.1005460 28410363 PMC5409497

[DMM047225C82] Pollard, J., Jr., Butte, A. J., Hoberman, S., Joshi, M., Levy, J. and Pappo, J. (2005). A computational model to define the molecular causes of type 2 diabetes mellitus. *Diabetes Technol. Ther.* 7, 323-336. 10.1089/dia.2005.7.323 15857235

[DMM047225C83] Prosser, H. M., Koike-Yusa, H., Cooper, J. D., Law, F. C. and Bradley, A. (2011). A resource of vectors and ES cells for targeted deletion of microRNAs in mice. *Nat. Biotechnol.* 29, 840-845. 10.1038/nbt.1929 21822254 PMC3242032

[DMM047225C84] Ritchie, M. E., Phipson, B., Wu, D., Hu, Y., Law, C. W., Shi, W. and Smyth, G. K. (2015). limma powers differential expression analyses for RNA-sequencing and microarray studies. *Nucleic Acids Res.* 43, e47 10.1093/nar/gkv007 25605792 PMC4402510

[DMM047225C85] Robinson, M. D., Mccarthy, D. J. and Smyth, G. K. (2010). edgeR: a Bioconductor package for differential expression analysis of digital gene expression data. *Bioinformatics* 26, 139-140. 10.1093/bioinformatics/btp616 19910308 PMC2796818

[DMM047225C86] Roux, I., Wu, J. S., Mcintosh, J. M. and Glowatzki, E. (2016). Assessment of the expression and role of the alpha1-nAChR subunit in efferent cholinergic function during the development of the mammalian cochlea. *J. Neurophysiol.* 116, 479-492. 10.1152/jn.01038.2015 27098031 PMC4978794

[DMM047225C87] Sakaguchi, N., Henzl, M. T., Thalmann, I., Thalmann, R. and Schulte, B. A. (1998). Oncomodulin is expressed exclusively by outer hair cells in the organ of Corti. *J. Histochem. Cytochem.* 46, 29-40. 10.1177/002215549804600105 9405492

[DMM047225C88] Sanchez-Martin, M., Rodriguez-Garcia, A., Perez-Losada, J., Sagrera, A., Read, A. P. and Sanchez-Garcia, I. (2002). SLUG (SNAI2) deletions in patients with Waardenburg disease. *Hum. Mol. Genet.* 11, 3231-3236. 10.1093/hmg/11.25.3231 12444107

[DMM047225C89] Schluter, T., Berger, C., Rosengauer, E., Fieth, P., Krohs, C., Ushakov, K., Steel, K. P., Avraham, K. B., Hartmann, A. K., Felmy, F. et al. (2018). miR-96 is required for normal development of the auditory hindbrain. *Hum. Mol. Genet.* 27, 860-874. 10.1093/hmg/ddy007 29325119

[DMM047225C90] Shannon, P., Markiel, A., Ozier, O., Baliga, N. S., Wang, J. T., Ramage, D., Amin, N., Schwikowski, B. and Ideker, T. (2003). Cytoscape: a software environment for integrated models of biomolecular interaction networks. *Genome Res.* 13, 2498-2504. 10.1101/gr.1239303 14597658 PMC403769

[DMM047225C91] Simmons, S. O., Fan, C.-Y. and Ramabhadran, R. (2009). Cellular stress response pathway system as a sentinel ensemble in toxicological screening. *Toxicol. Sci.* 111, 202-225. 10.1093/toxsci/kfp140 19567883

[DMM047225C92] Smith, C. L., Blake, J. A., Kadin, J. A., Richardson, J. E. and Bult, C. J. (2018). Mouse genome database (MGD)-2018: knowledgebase for the laboratory mouse. *Nucleic Acids Res.* 46, D836-D842. 10.1093/nar/gkx1006 29092072 PMC5753350

[DMM047225C93] Solda, G., Robusto, M., Primignani, P., Castorina, P., Benzoni, E., Cesarani, A., Ambrosetti, U., Asselta, R. and Duga, S. (2012). A novel mutation within the MIR96 gene causes non-syndromic inherited hearing loss in an Italian family by altering pre-miRNA processing. *Hum. Mol. Genet.* 21, 577-585. 10.1093/hmg/ddr493 22038834 PMC3259013

[DMM047225C94] Soukup, G. A., Fritzsch, B., Pierce, M. L., Weston, M. D., Jahan, I., Mcmanus, M. T. and Harfe, B. D. (2009). Residual microRNA expression dictates the extent of inner ear development in conditional Dicer knockout mice. *Dev. Biol.* 328, 328-341. 10.1016/j.ydbio.2009.01.037 19389351 PMC2793102

[DMM047225C95] Steigelman, K. A., Lelli, A., Wu, X., Gao, J., Lin, S., Piontek, K., Wodarczyk, C., Boletta, A., Kim, H., Qian, F. et al. (2011). Polycystin-1 is required for stereocilia structure but not for mechanotransduction in inner ear hair cells. *J. Neurosci.* 31, 12241-12250. 10.1523/JNEUROSCI.6531-10.2011 21865467 PMC3164988

[DMM047225C96] Tang, F., Chen, X., Jia, L., Li, H., Li, J. and Yuan, W. (2019). Differential gene expression patterns between apical and basal inner hair cells revealed by RNA-Seq. *Front. Mol. Neurosci.* 12, 332 10.3389/fnmol.2019.00332 32038162 PMC6985465

[DMM047225C97] Ten Cate, W.-J. F., Curtis, L. M., Small, G. M. and Rarey, K. E. (1993). Localization of glucocorticoid receptors and glucocorticoid receptor mRNAs in the rat cochlea. *Laryngoscope* 103, 865-871. 10.1288/00005537-199308000-00007 8361289

[DMM047225C98] The Gene Ontology Consortium (2019). The gene ontology resource: 20 years and still GOing strong. *Nucleic Acids Res.* 47, D330-D338. 10.1093/nar/gky1055 30395331 PMC6323945

[DMM047225C99] Tong, B., Hornak, A. J., Maison, S. F., Ohlemiller, K. K., Liberman, M. C. and Simmons, D. D. (2016). Oncomodulin, an EF-Hand Ca2+ buffer, is critical for maintaining cochlear function in mice. *J. Neurosci.* 36, 1631-1635. 10.1523/JNEUROSCI.3311-15.2016 26843644 PMC4737773

[DMM047225C100] Tong, Z., Cui, Q., Wang, J. and Zhou, Y. (2019). TransmiR v2.0: an updated transcription factor-microRNA regulation database. *Nucleic Acids Res.* 47, D253-D258. 10.1093/nar/gky1023 30371815 PMC6323981

[DMM047225C101] Trapnell, C., Williams, B. A., Pertea, G., Mortazavi, A., Kwan, G., van Baren, M. J., Salzberg, S. L., Wold, B. J. and Pachter, L. (2010). Transcript assembly and quantification by RNA-Seq reveals unannotated transcripts and isoform switching during cell differentiation. *Nat. Biotechnol.* 28, 511-515. 10.1038/nbt.1621 20436464 PMC3146043

[DMM047225C102] Untergasser, A., Cutcutache, I., Koressaar, T., Ye, J., Faircloth, B. C., Remm, M. and Rozen, S. G. (2012). Primer3--new capabilities and interfaces. *Nucleic Acids Res.* 40, e115 10.1093/nar/gks596 22730293 PMC3424584

[DMM047225C103] Van Dongen, S., Abreu-Goodger, C. and Enright, A. J. (2008). Detecting microRNA binding and siRNA off-target effects from expression data. *Nat. Methods* 5, 1023-1025. 10.1038/nmeth.1267 18978784 PMC2635553

[DMM047225C104] Vreugde, S., Erven, A., Kros, C. J., Marcotti, W., Fuchs, H., Kurima, K., Wilcox, E. R., Friedman, T. B., Griffith, A. J., Balling, R. et al. (2002). Beethoven, a mouse model for dominant, progressive hearing loss DFNA36. *Nat. Genet.* 30, 257-258. 10.1038/ng848 11850623

[DMM047225C105] Walsh, T., Walsh, V., Vreugde, S., Hertzano, R., Shahin, H., Haika, S., Lee, M. K., Kanaan, M., King, M. C. and Avraham, K. B. (2002). From flies’ eyes to our ears: mutations in a human class III myosin cause progressive nonsyndromic hearing loss DFNB30. *Proc. Natl. Acad. Sci. USA* 99, 7518-7523. 10.1073/pnas.102091699 12032315 PMC124268

[DMM047225C106] Walsh, V. L., Raviv, D., Dror, A. A., Shahin, H., Walsh, T., Kanaan, M. N., Avraham, K. B. and King, M. C. (2011). A mouse model for human hearing loss DFNB30 due to loss of function of myosin IIIA. *Mamm. Genome* 22, 170-177. 10.1007/s00335-010-9310-6 21165622 PMC8023428

[DMM047225C107] Wei, K., Chen, J., Akrami, K., Galbraith, G. C., Lopez, I. A. and Chen, F. (2007). Neural crest cell deficiency of c-myc causes skull and hearing defects. *Genesis* 45, 382-390. 10.1002/dvg.20304 17523175

[DMM047225C108] Weston, M. D., Pierce, M. L., Rocha-Sanchez, S., Beisel, K. W. and Soukup, G. A. (2006). MicroRNA gene expression in the mouse inner ear. *Brain Res.* 1111, 95-104. 10.1016/j.brainres.2006.07.006 16904081

[DMM047225C109] Weston, M. D., Tarang, S., Pierce, M. L., Pyakurel, U., Rocha-Sanchez, S. M., Mcgee, J., Walsh, E. J. and Soukup, G. A. (2018). A mouse model of miR-96, miR-182 and miR-183 misexpression implicates miRNAs in cochlear cell fate and homeostasis. *Sci. Rep.* 8, 3569 10.1038/s41598-018-21811-1 29476110 PMC5824881

[DMM047225C110] Xu, S., Witmer, P. D., Lumayag, S., Kovacs, B. and Valle, D. (2007). MicroRNA (miRNA) transcriptome of mouse retina and identification of a sensory organ-specific miRNA cluster. *J. Biol. Chem.* 282, 25053-25066. 10.1074/jbc.M700501200 17597072

[DMM047225C111] Zerbino, D. R., Achuthan, P., Akanni, W., Amode, M. R., Barrell, D., Bhai, J., Billis, K., Cummins, C., Gall, A., Giron, C. G. et al. (2018). Ensembl 2018. *Nucleic Acids Res.* 46, D754-D761. 10.1093/nar/gkx1098 29155950 PMC5753206

[DMM047225C112] Zhang, B. and Horvath, S. (2005). A general framework for weighted gene co-expression network analysis. *Stat. Appl. Genet. Mol. Biol.* 4, Article17 10.2202/1544-6115.1128 16646834

[DMM047225C113] Zhang, J., Liu, Z., Chang, A., Fang, J., Men, Y., Tian, Y., Ouyang, X., Yan, D., Zhang, A., Sun, X. et al. (2016). Abnormal mRNA splicing but normal auditory brainstem response (ABR) in mice with the prestin (SLC26A5) IVS2-2A>G mutation. *Mutat. Res.* 790, 1-7. 10.1016/j.mrfmmm.2016.05.004 27232762 PMC5345126

[DMM047225C114] Zine, A., Van De Water, T. R. and De Ribaupierre, F. (2000). Notch signaling regulates the pattern of auditory hair cell differentiation in mammals. *Development* 127, 3373-3383. 10887092 10.1242/dev.127.15.3373

