# Supplementary material for: Hearing impairment due to Mir183/96/182 mutations suggests both loss-of-function and gain-of-function effects
Source: Dis Model Mech. 2021 Feb 15;14(2):dmm047225. doi: 10.1242/dmm.047225 (PMC7903918; doi:10.1242/dmm.047225)
Supplement: Supplementary information [file dmm-14-047225-s1.pdf]

Supporting Information

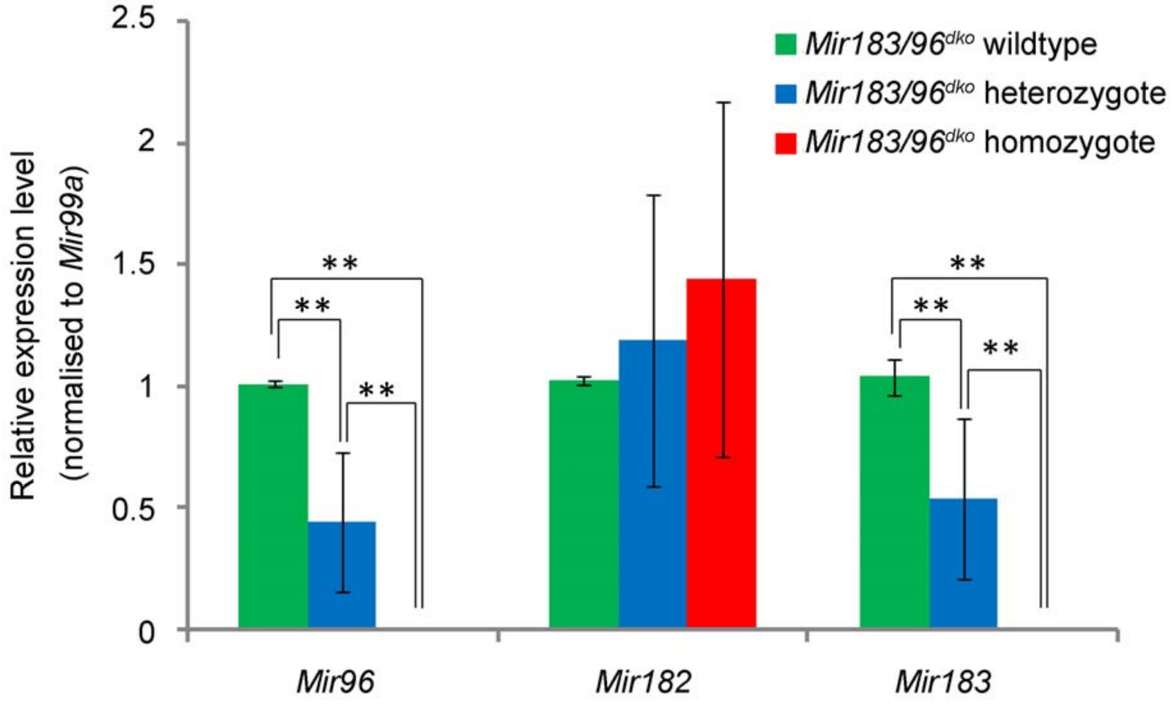

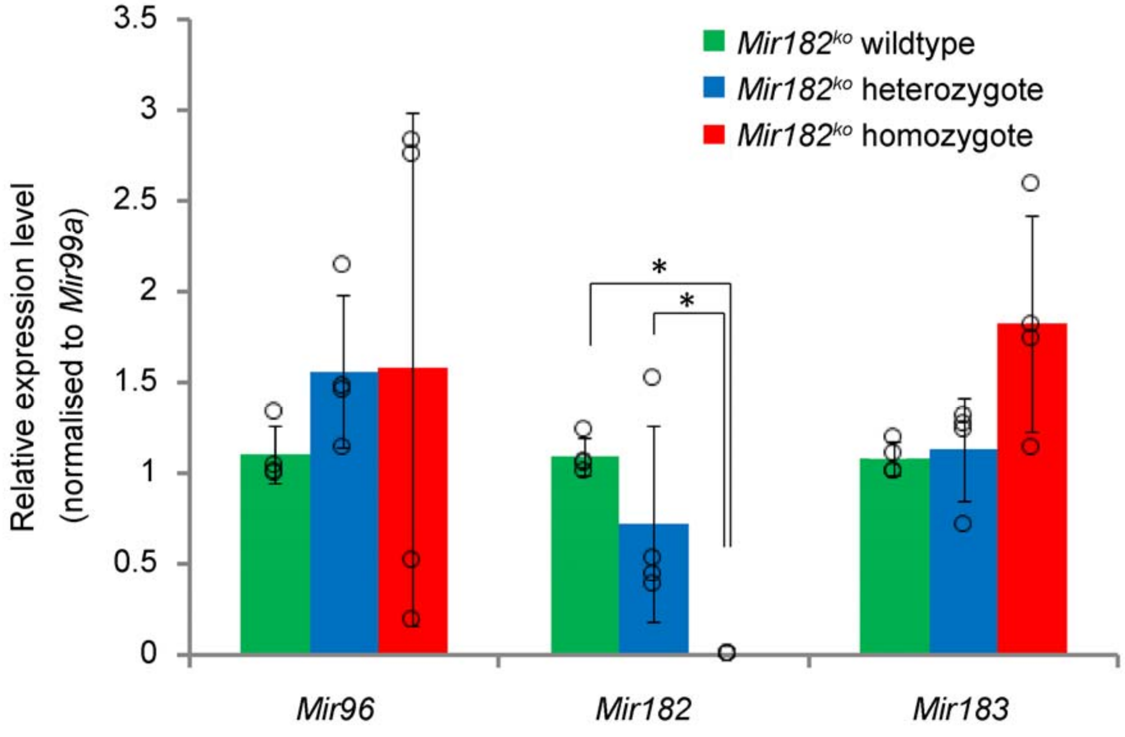

Disease Models & Mechanisms • Supplementary information

Figure S1. Expression of *Mir96*, *Mir182* and *Mir183* in *Mir183/96^{dko}* mutant mice (top) and *Mir182^{ko}* mutant mice (bottom). Mean expression levels (bars) were calculated from individual expression levels from each mouse, normalised to expression in a wildtype littermate, and relative to *Mir99a*, which is expressed in cochlear sensory epithelium. Error bars are standard deviation. Three technical replicates were carried out for each experiment. *Mir183/96^{dko}*: wildtype n=7, heterozygote n=5, homozygote n=6. One way ANOVA: *Mir96* $p<0.001$ (wildtype vs. heterozygote Bonferroni-corrected $p<0.001$; wildtype vs. homozygote Bonferroni-corrected $p<0.001$; heterozygote vs. homozygote Bonferroni-corrected p=0.001) ; *Mir182* p=0.37; *Mir183* $p<0.001$ (wildtype vs. heterozygote Bonferroni-corrected p=0.001; wildtype vs. homozygote Bonferroni-corrected $p<0.001$; heterozygote vs. homozygote Bonferroni-corrected $p<0.001$). *Mir182^{ko}*: wildtype n=4, heterozygote n=4, homozygote n=4; individual data points are plotted as outlined circles. One way ANOVA: *Mir96* p=0.685; *Mir182* p=0.003 (wildtype vs. heterozygote Bonferroni-corrected p=0.397; wildtype vs. homozygote Bonferroni-corrected p=0.003; heterozygote vs. homozygote Bonferroni-corrected p=0.032); *Mir183* p=0.04 (wildtype vs. heterozygote Bonferroni-corrected p=1.0; wildtype vs. homozygote Bonferroni-corrected p=0.068; heterozygote vs. homozygote Bonferroni-corrected p=0.094), Error bars are standard deviation (* = $P < 0.05$, ** = $P \leq 0.01$).

Disease Models & Mechanisms • Supplementary information

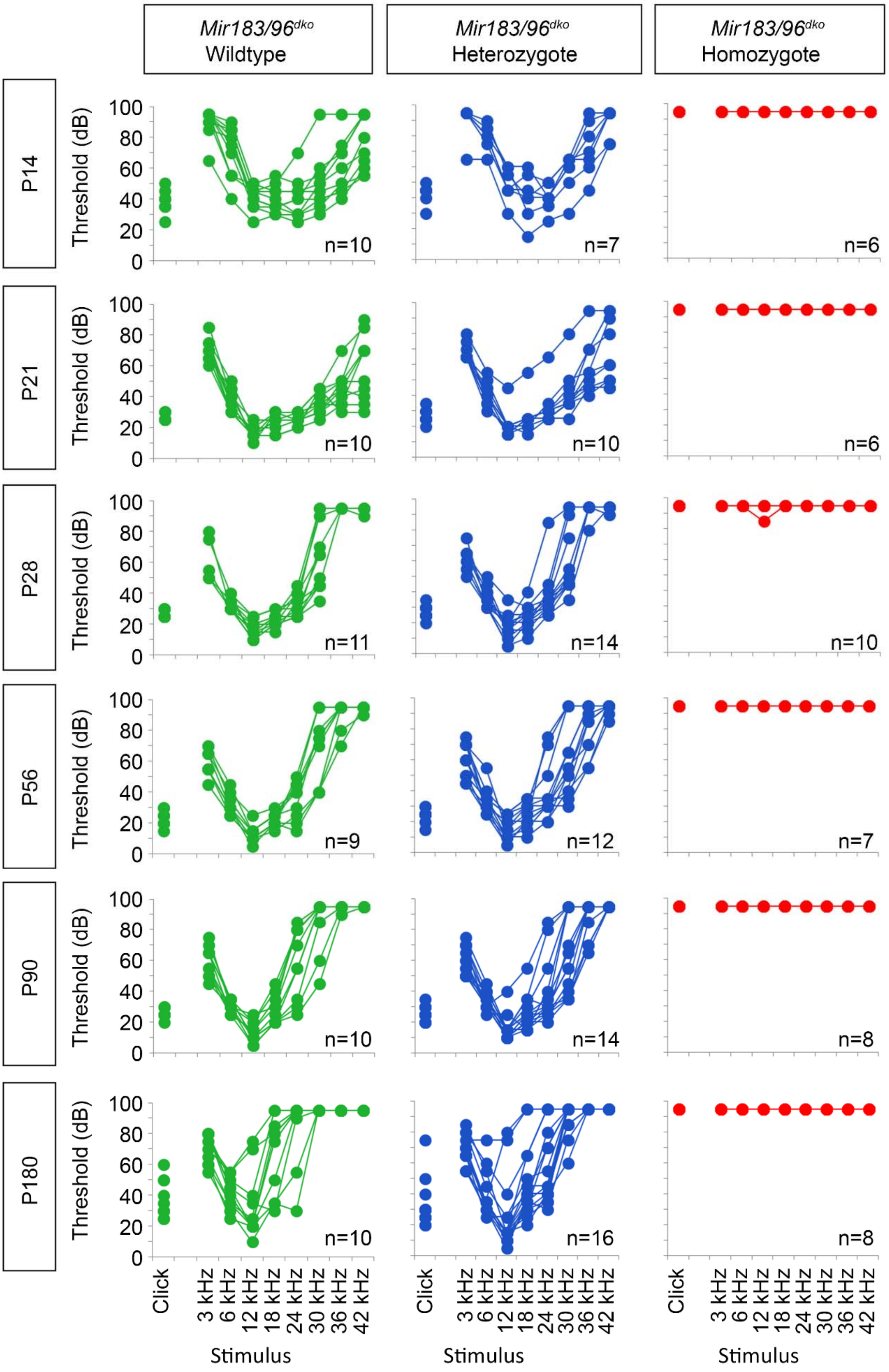

Disease Models & Mechanisms • Supplementary information

Figure S2. Individual ABR thresholds of wildtype, heterozygous and homozygous *Mir183/96*^{dko} mice at all ages tested. Number of mice of each genotype tested at each age is shown on the threshold plot.

Disease Models & Mechanisms • Supplementary information

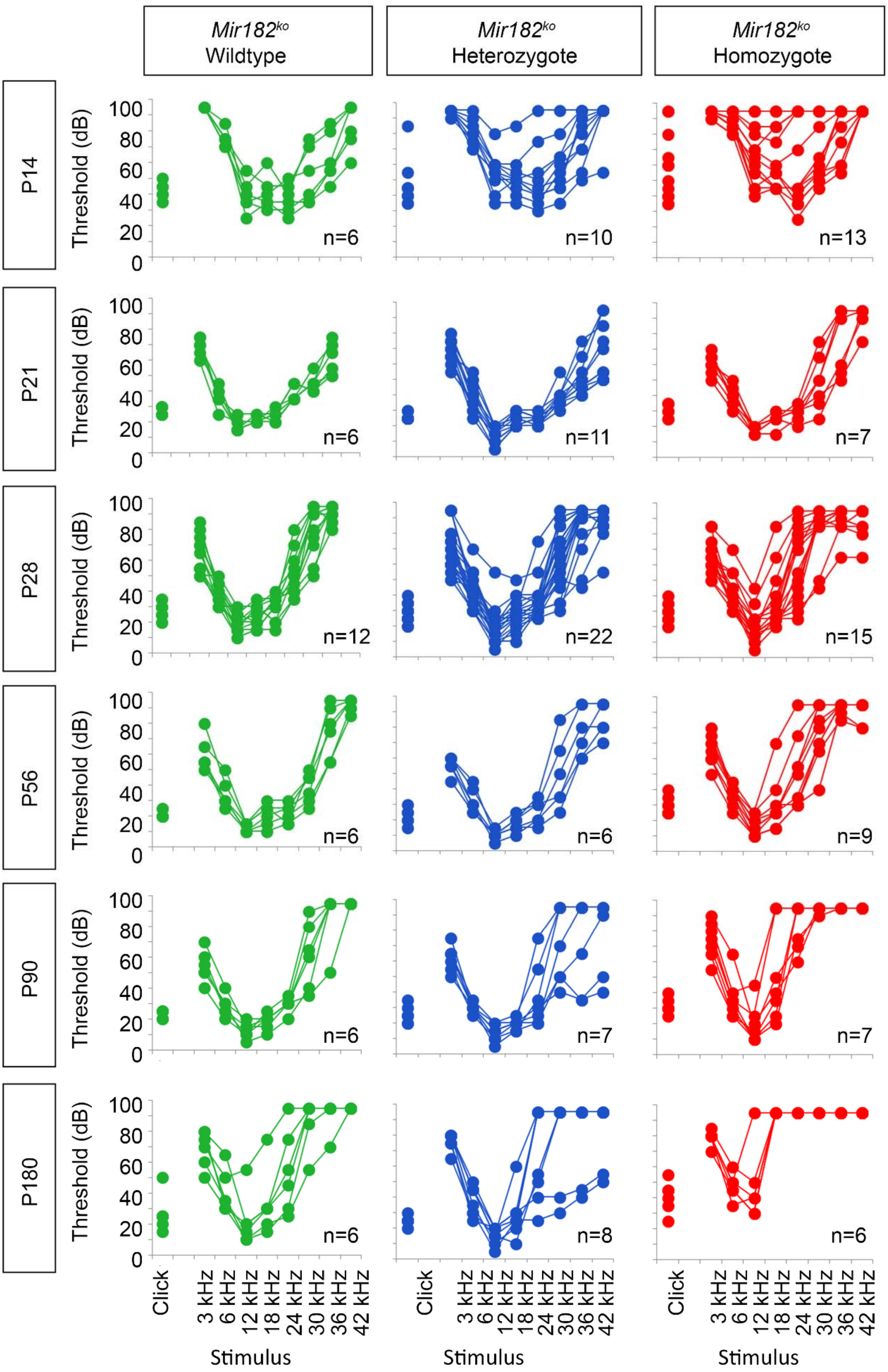

Disease Models & Mechanisms • Supplementary information

Figure S3. Individual ABR thresholds of wildtype, heterozygous and homozygous *Mir182*^{ko} mice at all ages tested. Number of mice of each genotype tested at each age is shown on the threshold plot.

Disease Models & Mechanisms • Supplementary information

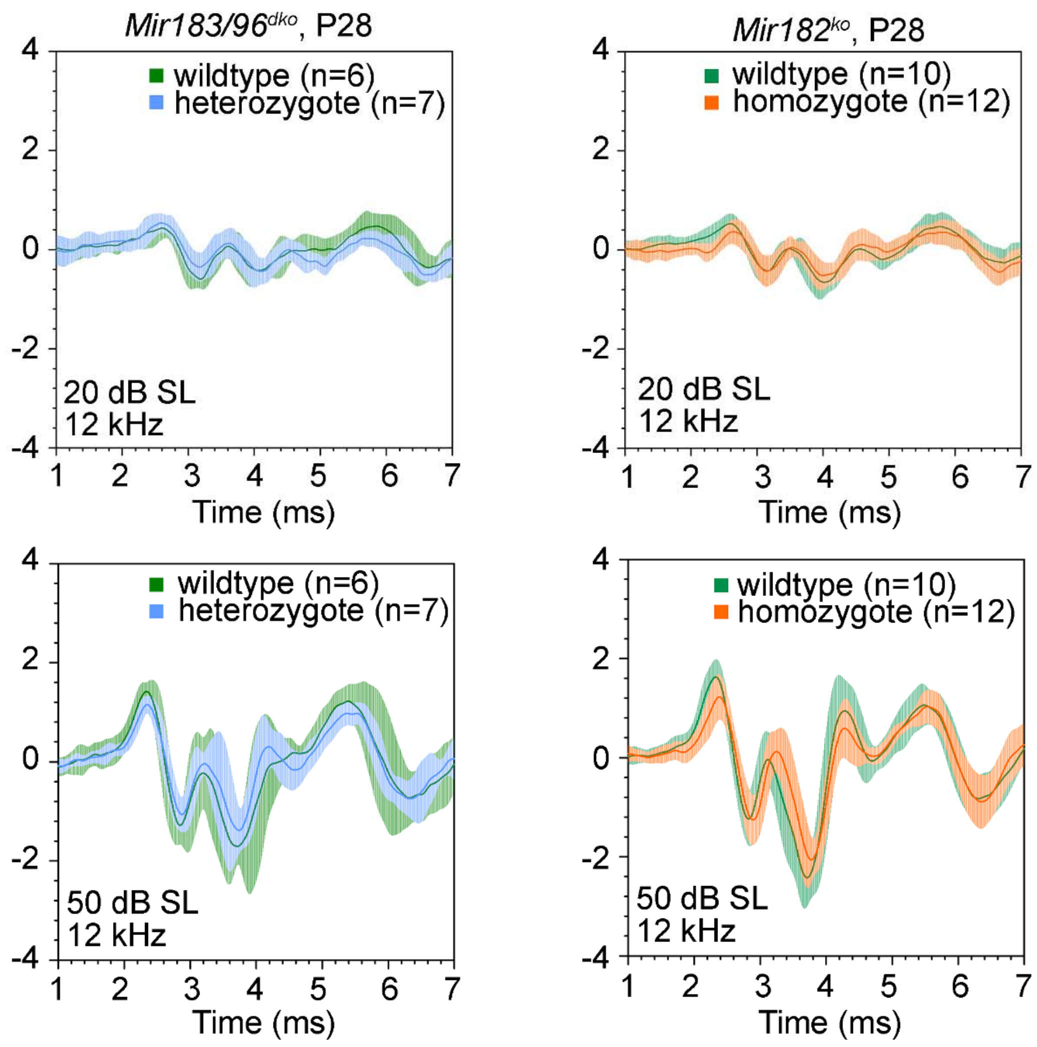

Figure S4. Mean ABR waveforms at 12 kHz, shown at 20 dB (top) and 50 dB (bottom) above threshold (sensation level, SL) ± standard deviation, at four weeks old. There is no obvious difference between *Mir183/96*^{dko} heterozygous (blue, n=7) and wildtype mice (green, n=6) (left), or between *Mir182*^{ko} homozygous (orange, n=12) and wildtype mice (green, n=10) (right) at either sensation level.

Disease Models & Mechanisms • Supplementary information

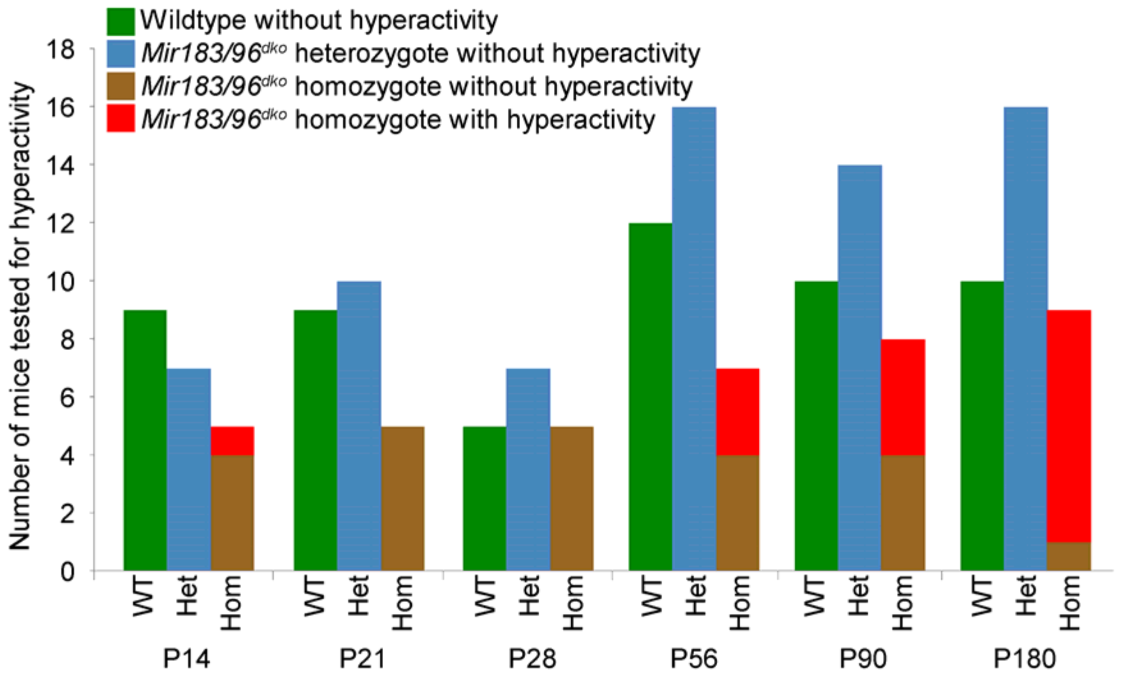

Figure S5. Numbers of mice assessed for hyperactivity prior to ABR at different ages in wildtype (green, WT), heterozygote (blue, Het) and homozygote (red and brown, Hom) *Mir183/96*^{dko} mice. Only homozygotes showed any vestibular phenotype, the incidence of which increased with age. Bright red indicates homozygous mice with hyperactive behaviour, and brown indicates homozygotes without hyperactivity.

Disease Models & Mechanisms • Supplementary information

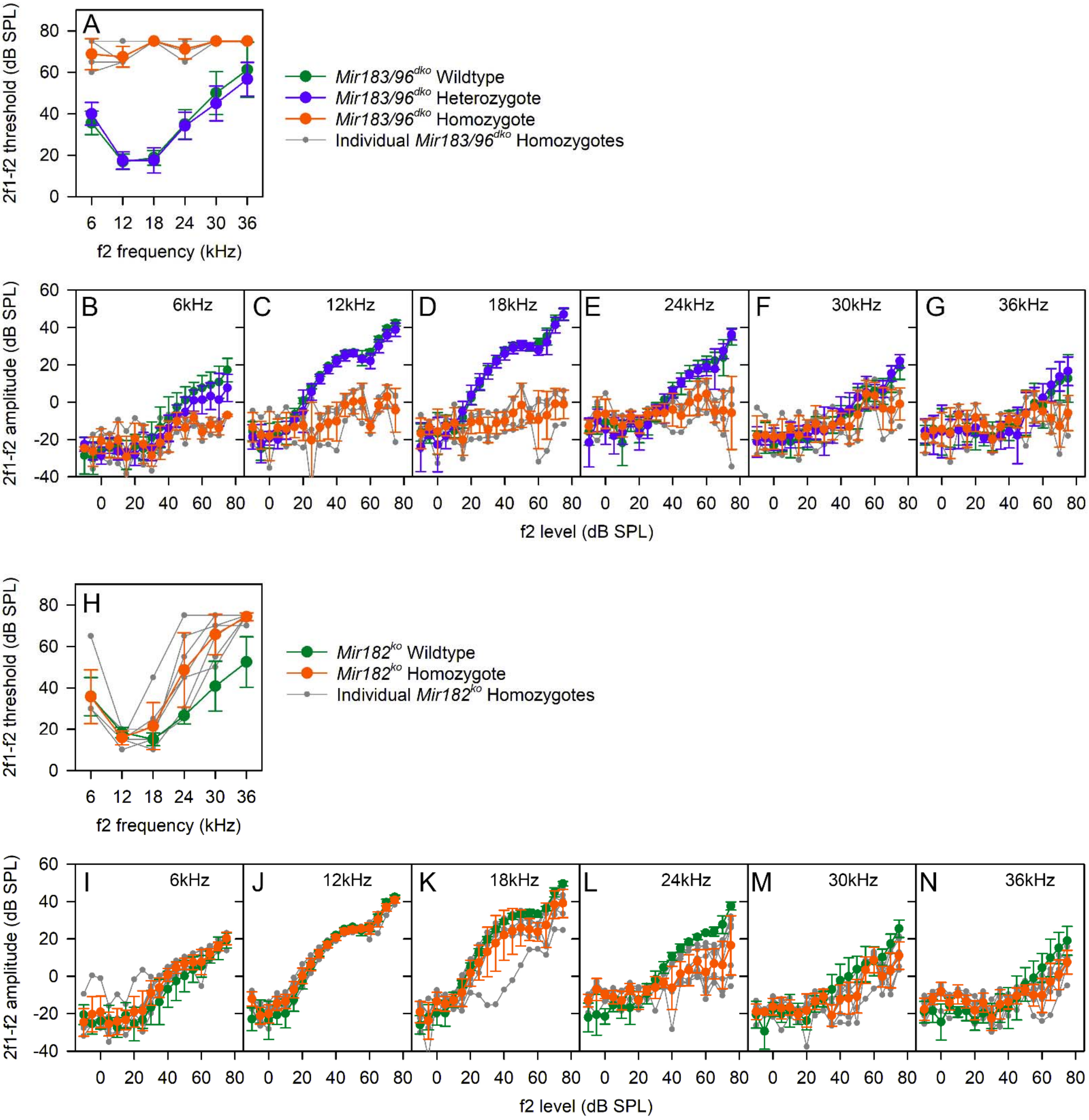

Figure S6. Distortion Product Otoacoustic Emission (DPOAE) measurements from *Mir183/96^{dko}* and *Mir182^{ko}* mice. (A-G) DPOAEs recorded from *Mir183/96^{dko}* mice; wildtype n=8, heterozygote n=6, homozygote n=4. (H-N) DPOAEs recorded from *Mir182^{ko}* mice; wildtype n=6, homozygote n=7. Mean

Disease Models & Mechanisms • Supplementary information

responses (±standard deviation) are indicated by green (wildtype), purple (heterozygote) and orange (homozygote) lines & symbols. Responses from individual homozygote animals are indicated in grey lines & symbols. (A, H) The threshold of the 2f1-f2 DPOAE (as defined in the methods) is plotted as a function of f2 frequency. (B-G, I-N) The amplitude of the 2f1-f2 DPOAE is plotted as a function of f2 level (dB SPL) for the range of f2 tones used; 6 kHz (B & I), 12 kHz (C & J), 18 kHz (D & K), 24 kHz (E & L), 30 kHz (F & M) and 36 kHz (G & N).

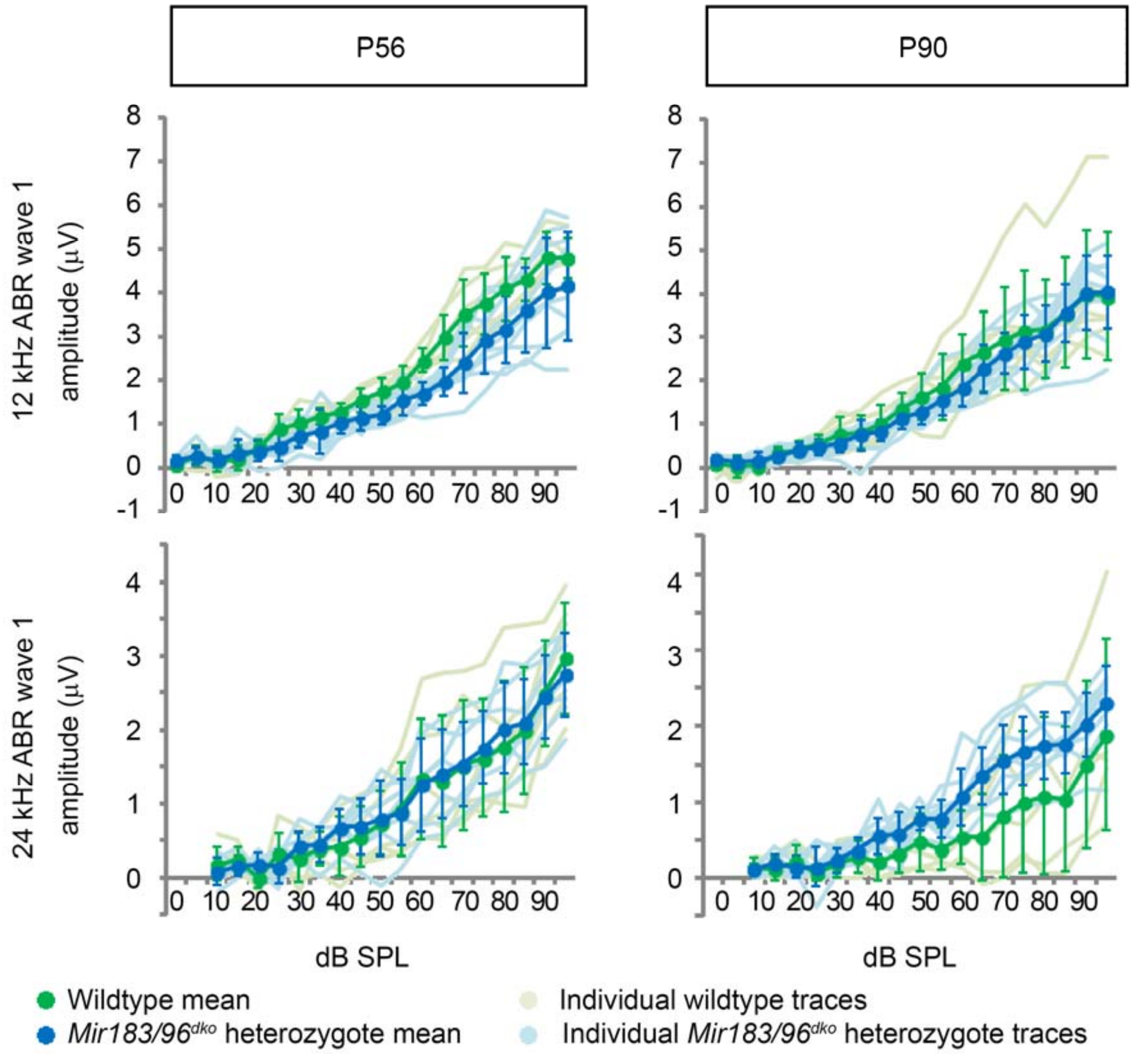

Figure S7. Mean ABR wave 1 amplitudes at 12 kHz and 24 kHz for *Mir183/96*^{dko} wildtype and heterozygous mice at P56 and P90. Wildtypes are shown in green (n=6 at P56, n=7 at P90) and heterozygotes in blue (n=9 at P56, n=12 at P90). Individual wave 1 amplitudes are also plotted in pale green (wildtype) and pale blue (heterozygote). Heterozygous amplitudes appear similar to wildtype at both ages and both frequencies. Error bars are standard deviation.

Disease Models & Mechanisms • Supplementary information

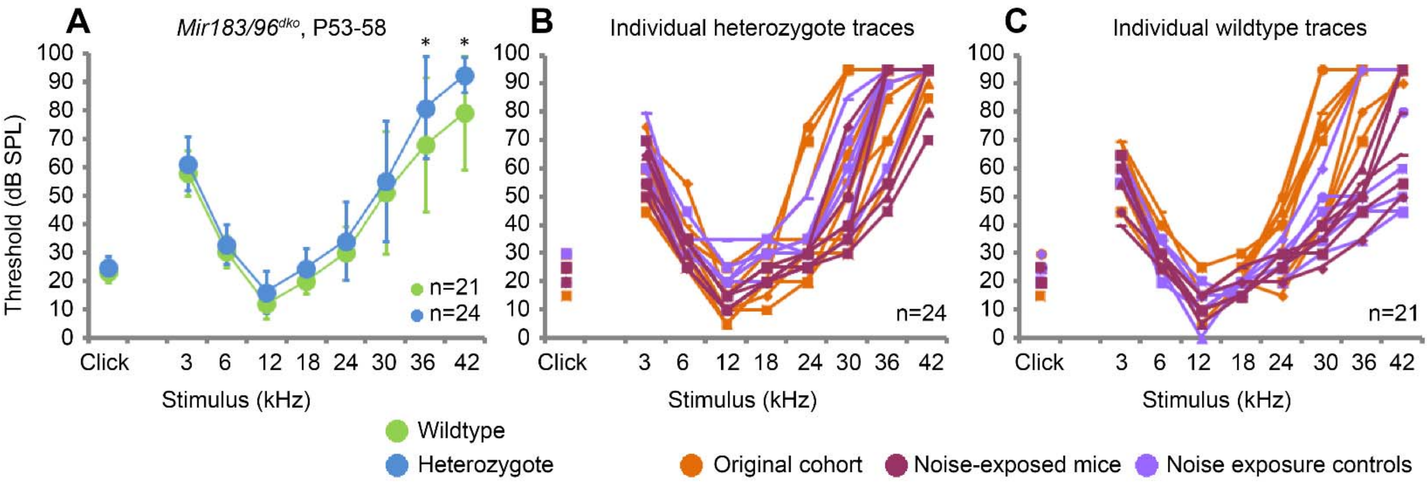

Figure S8. All ABR thresholds from *Mir183/96^{dko}* heterozygous and wildtype mice at 8 weeks old (53-58 days). (A) Means of all mice tested. Heterozygotes are shown in blue (n=24) and wildtypes in green (n=21). Error bars are standard deviation (* = Bonferroni-corrected $p < 0.05$, mixed linear model pairwise comparison; see supplementary data for all p-values). (B) Individual thresholds from heterozygous mice. (C) Individual thresholds from wildtype mice. In (B) and (C), the original mice tested are coloured orange, the noise-exposed mice are coloured dark maroon and the noise exposure control mice are coloured lilac.

Disease Models & Mechanisms • Supplementary information

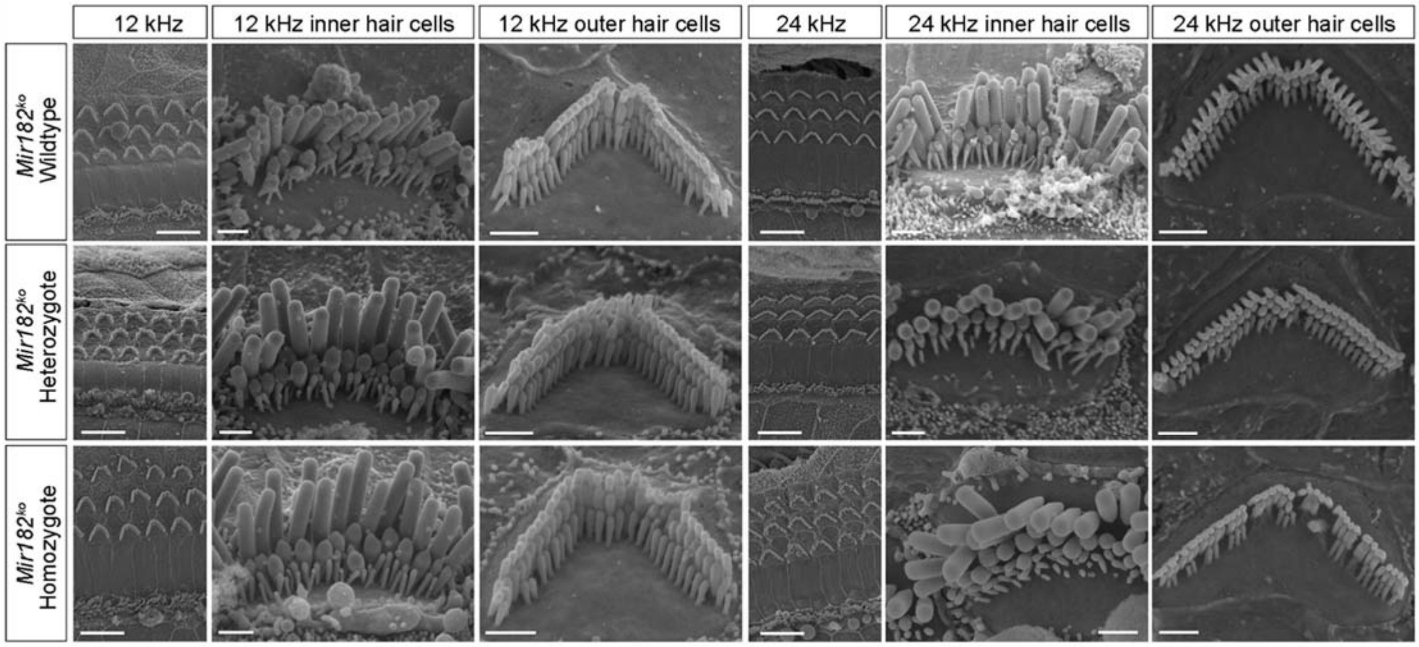

Figure S9. Scanning electron micrographs of *Mir182^{ko}* mice at P28. Two best-frequency regions of the organ of Corti are shown; 12 kHz (68% of the way along the organ of Corti from base to apex) and 24 kHz (43% of the way along the organ of Corti from base to apex). For each region, the left-hand column shows a zoomed-out image with inner and outer hair cell rows (scale bars=10 μm), and the other two columns show an inner and an outer hair cell close up (scale bars=1 μm). The top row shows wildtype hair cells (n=1), the middle row shows heterozygote hair cells (n=2) and the bottom row shows homozygote hair cells (n=1).

Disease Models & Mechanisms • Supplementary information

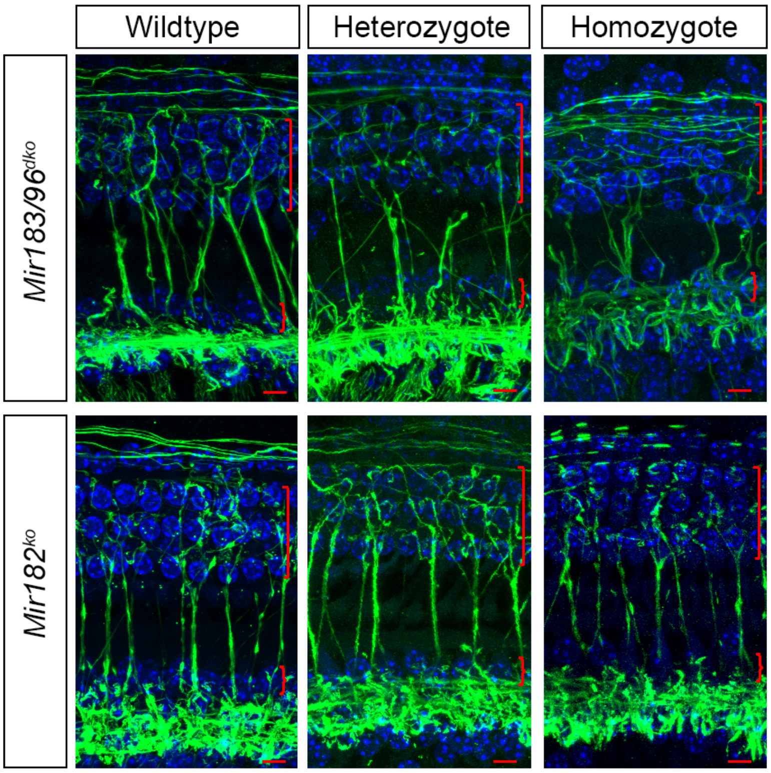

Figure S10. Innervation of inner and outer hair cells of *Mir183/96^{dko}* , and *Mir182^{ko}* mice at P28.

Nerve fibres are stained with anti-neurofilament antibody (green) and nuclei are labelled with DAPI (blue). Mir183/*96^{dko}*: wildtype n=9, heterozygote n=8, homozygote n=4. *Mir182^{e}*: wildtype n=5, heterozygote n=6, homozygote n=10. All panels show the 12 kHz best-frequency region. Square brackets indicate the three rows of outer hair cell nuclei, and curly brackets the single row of inner hair cell nuclei. Scale bar = 5 µm.

Disease Models & Mechanisms • Supplementary information

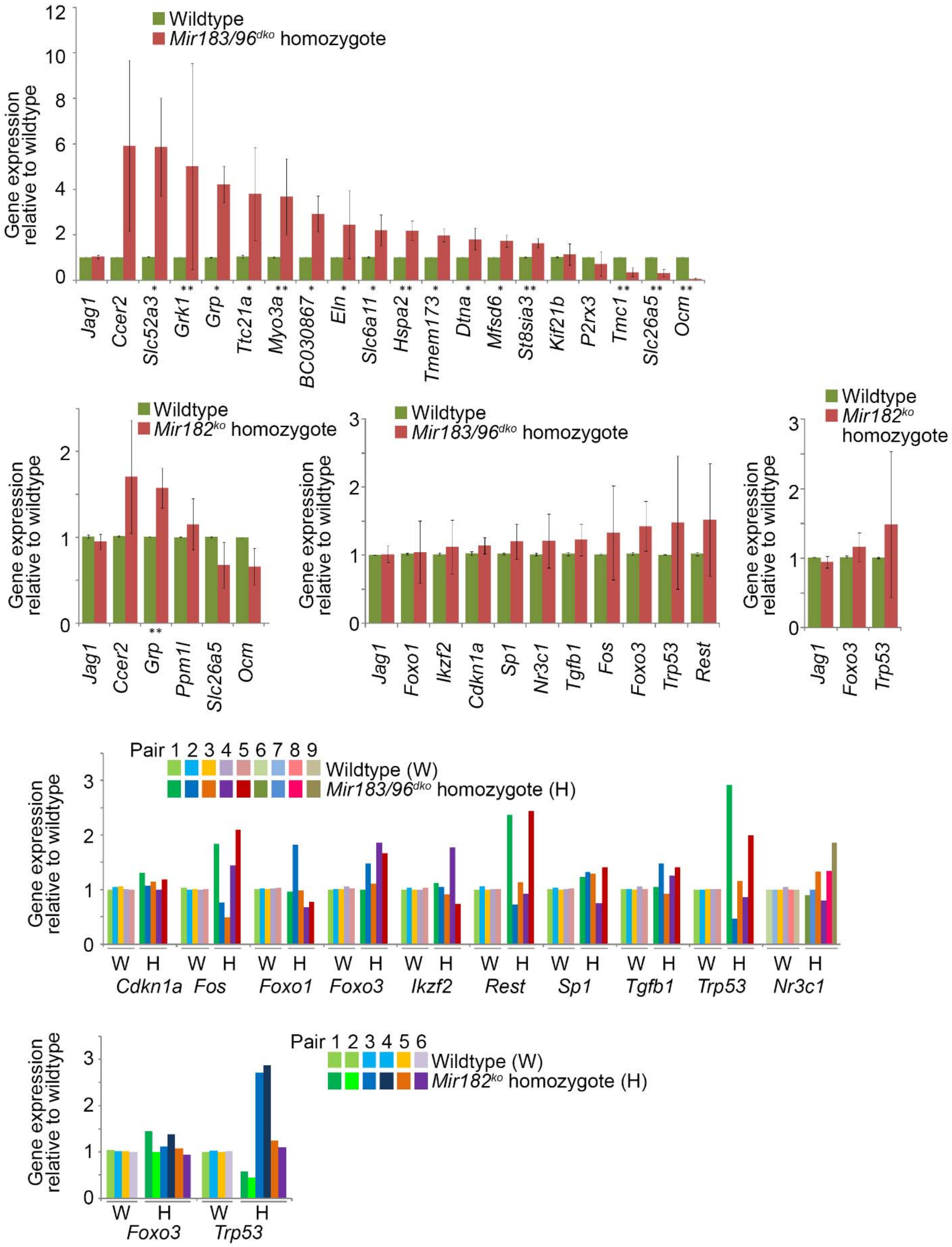

Figure S11. Confirmation testing of RNA-seq results and testing of network nodes. qPCR was carried out on cDNA from P4 organs of Corti in wildtype (green; left bar in A-D) and homozygote

Disease Models & Mechanisms • Supplementary information

(red; right bar in A-D) littermates to test gene expression changes. Mean expression levels were calculated from individual expression levels from each mouse, normalised to expression in a wildtype littermate, and relative to *Hprt*, with the exception of *Ocm* and *Slc26a5*, which were normalised to *Jag1*, which is expressed in supporting cells. At least three technical replicates were carried out for each experiment. (E) and (F) show the expression levels of the network genes from (C) and (D) in individual wildtype-homozygote pairs, showing the high variability between mice. Error bars are standard deviation (* = P < 0.05, ** = P < 0.01). All p-values were calculated using the Wilcoxon rank sum test (two-tailed, no adjustment for multiple testing). (A) Testing RNA-seq results for *Mir183/96*^{dko}. *Jag1* n=7 pairs, p=0.38; *Ccer2* n=6 pairs, p=0.065; *Slc52a3* n=6, pairs, p=0.0022; *Grk1* n=6 pairs, p=0.0022; *Grp* n=6 pairs, p= 0.0022; *Myo3a* n=6 pairs, p=0.0022; *Ttc21a* n=6 pairs, p=0.065; *BC030867* n=6 pairs, p=0.0022; *Slc6a11* n=6 pairs, p=0.0022; *Eln* n=6 pairs, p=0.0022; *Hspa2* n=6 pairs, p=0.0022; *Tmem173* n=6 pairs, p=0.0022; *Mfsd6* n=6 pairs, p=0.0022; *Dtna* n=6 pairs, p=0.0022; *St8sia3* n=6 pairs, p=0.015; *Kif21b* n=6 pairs, p=0.39; *P2rx3* n=6 pairs, p=0.065; *Tmc1* n=6 pairs, p=0.0022; *Slc26a5* n=6 wildtypes, 7 homozygotes, p=0.0012; *Ocm* n=6 wildtypes, 7 homozygotes, p=0.0012. (B) Testing RNA-seq results for *Mir182*^{ko}. *Jag1* n=6 pairs, p=0.70; *Ccer2* n=6 pairs, p=0.065; *Grp* n=6 pairs, p=0.0022; *Ppm1l* n=6 pairs, p=0.39; *Slc26a5* n=6 pairs, p=0.065; *Ocm* n=6 pairs, p=0.065. (C, E) Testing network nodes in *Mir183/96*^{dko}. *Jag1* n=9 pairs, p=0.73; *Foxo1* n=5 pairs, p=0.15; *Ikzf2* n=5 pairs, p=0.69; *Cdkn1a* n=5 pairs, p=0.15; *Sp1* n=5 pairs, p=0.15; *Nr3c1* n=6 pairs, p=1; *Tgfb1* n=5 pairs, p=0.22; *Fos* n=5 pairs, p=0.69; *Foxo3* n=5 pairs, p=0.15; *Trp53* n=5 pairs, p=0.69; *Rest* n=5 pairs, p=0.69. (D, F) Testing network nodes ion *Mir182*^{ko}. *Jag1* n=4 wildtypes, 6 homozygotes, p= 0.11; *Foxo3* n=4 wildtypes, 6 homozygotes p=0.48; *Trp53* n=4 wildtypes, 6 homozygotes p=0.48.

Disease Models & Mechanisms • Supplementary information

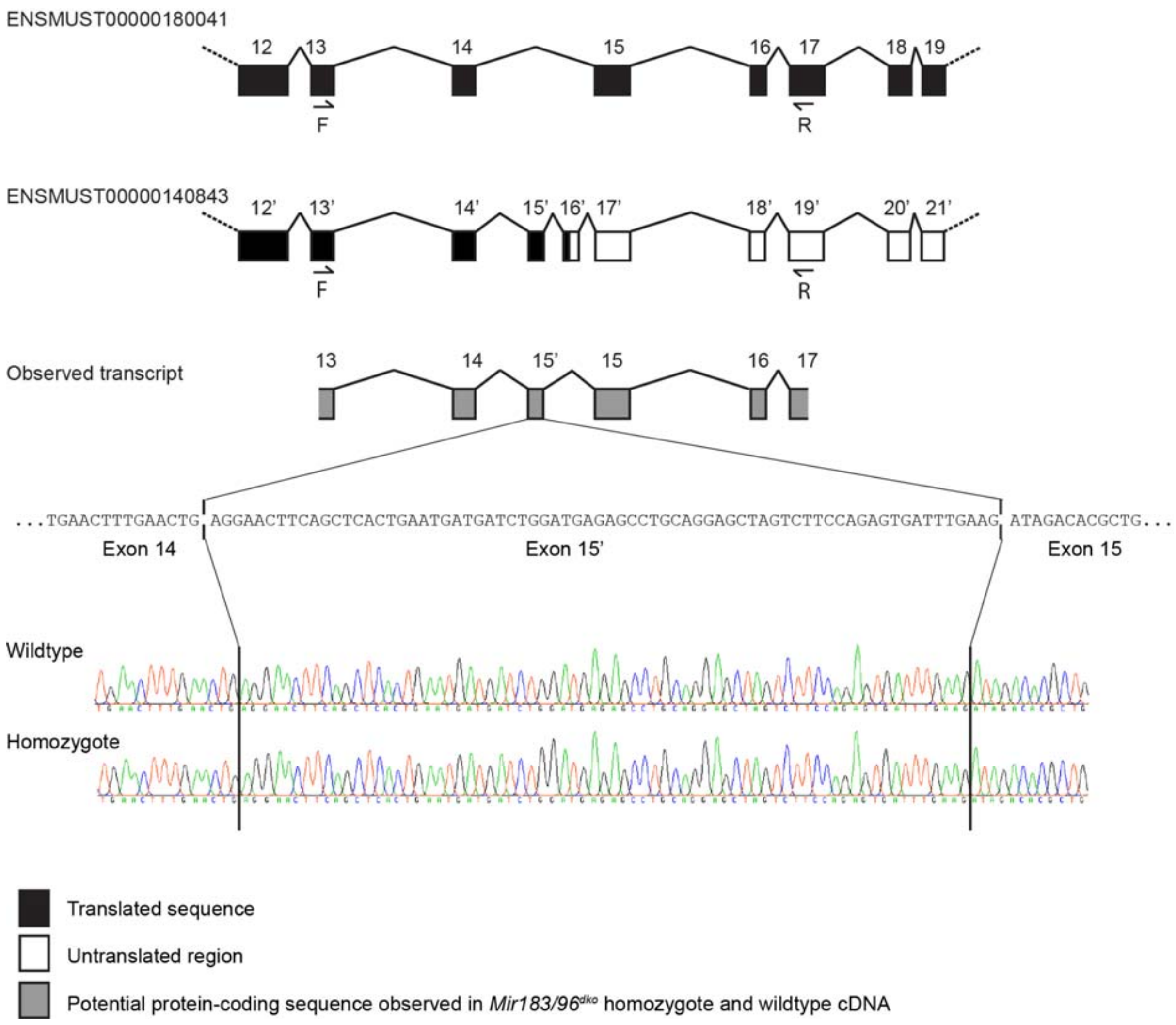

Figure S12. Schematic of the novel splice pattern in *Stard9* predicted by JunctionSeq and observed in 4 wildtype and 4 homozygous *Mir183/96*^{dko} mice. Exons 12-19 of the Ensembl protein-coding transcript ENSMUST00000180041 are shown at the top, and exons 12-21 of the nonsense-mediated decay transcript ENSMUST00000140843 underneath. We sequenced exons 13-17 from the protein-coding transcript (the positions of the primers used are marked with “F” and “R”) and found an exon between exons 14 and 15 corresponding to exon 15’ from the nonsense-mediated decay transcript (ENSMUSE00001437951). The sequence and traces are shown at the bottom. Exon 15’ does not introduce a frameshift, and its inclusion may result in a functional Stard9 protein with 24 extra amino acids. Both wildtype and homozygous sequences included exon 15’ and neither showed any sign of alternative splicing around it.

Disease Models & Mechanisms • Supplementary information

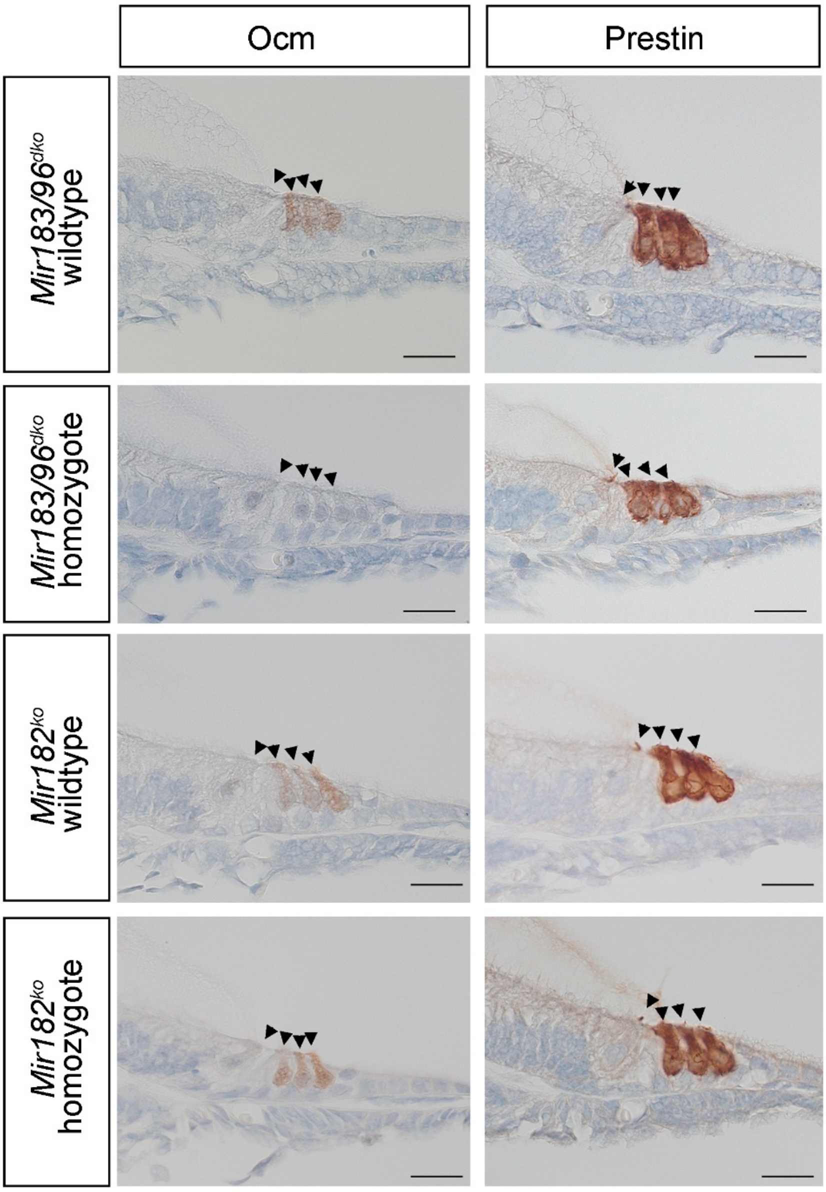

Figure S13. Ocm (left) and Prestin (right) antibody stains in *Mir183/96*^{dko} wildtypes and homozygotes , and *Mir182*^{ko} wildtypes and homozygotes. No Ocm stain is visible in *Mir183/96*^{dko} homozygotes. Hair cells are indicated by arrowheads. Scale bar = 10 µm. 3 homozygotes and 3 wildtype littermates were tested with each antibody.

Disease Models & Mechanisms • Supplementary information

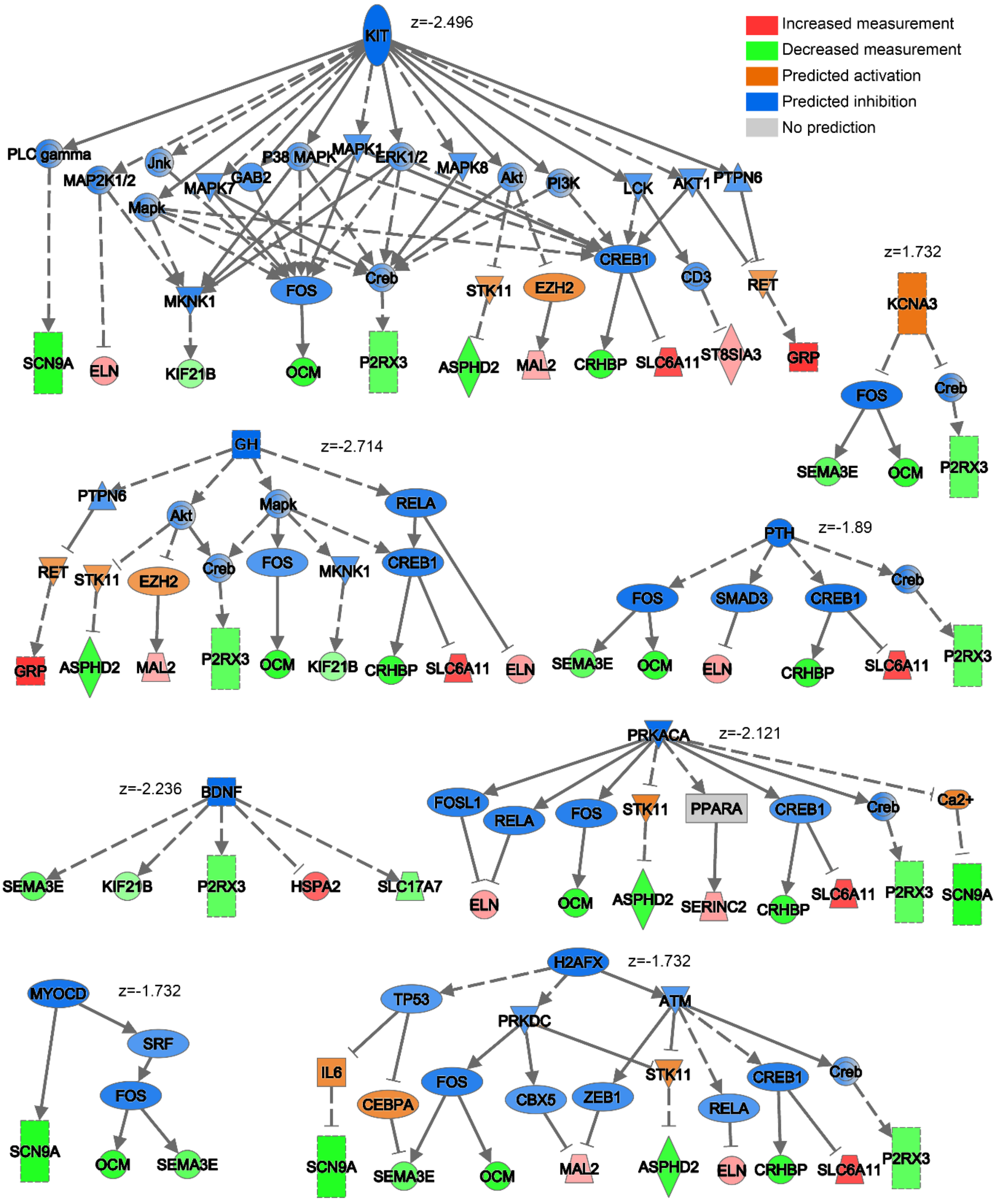

Figure S14. Networks generated by Ingenuity Pathway Analysis from the *Mir183/96*^{dko} RNA-seq data, showing predicted upstream regulators which may be responsible for some of the misregulation observed in the data. Misregulated genes are arranged on the lowest row, coloured according to observed misregulation (pink/red = upregulated, green = downregulated in mutants).

The top row(s) contain predicted regulators (orange = predicted upregulation, blue = predicted downregulation). Predicted links inconsistent with the observed misregulation have been removed. The intensity of the colour indicates the level of observed or predicted misregulation. Dotted lines represent indirect regulation, and solid lines direct regulation. The z-score of each network, which is both a prediction of the direction of misregulation of the root regulator and a measure of the match of observed and predicted gene misregulation, is shown in the figure. A significant z-score is one with an absolute value greater than 2. A negative score indicates downregulation and a positive score upregulation of the root regulator. miR-96 is not one of the identified upstream regulators.

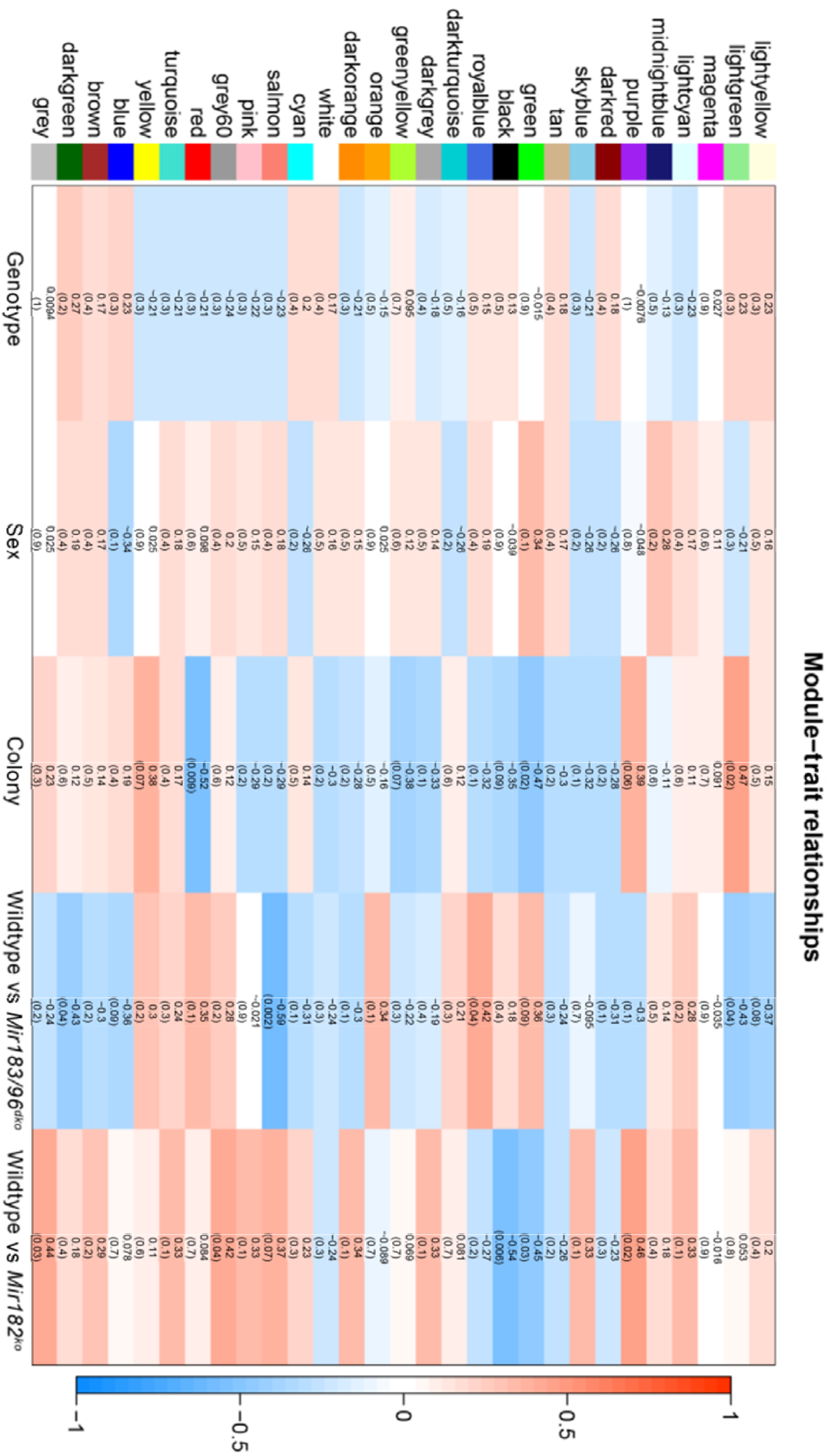
Module−trait relationships
lightyellow
lightgreen
magenta
lightcyan
midnightblue
purple
darkred
skyblue
tan
green
black
royalblue
darkturquoise
darkgrey
greenyellow
orange
darkorange
white
cyan
salmon
pink
grey60
red
turquoise
yellow
blue
brown
darkgreen
grey
Genotype
Sex
Colony
Wildtype vs *Mir183/96^dko^*
Wildtype vs *Mir182^ko^*
1
0.5
0
−0.5
−1

Figure S15. Associations of the different module eigengenes (rows) with traits (columns). Cell colour indicates correlation level; each cell contains the correlation score and the p-value in brackets.

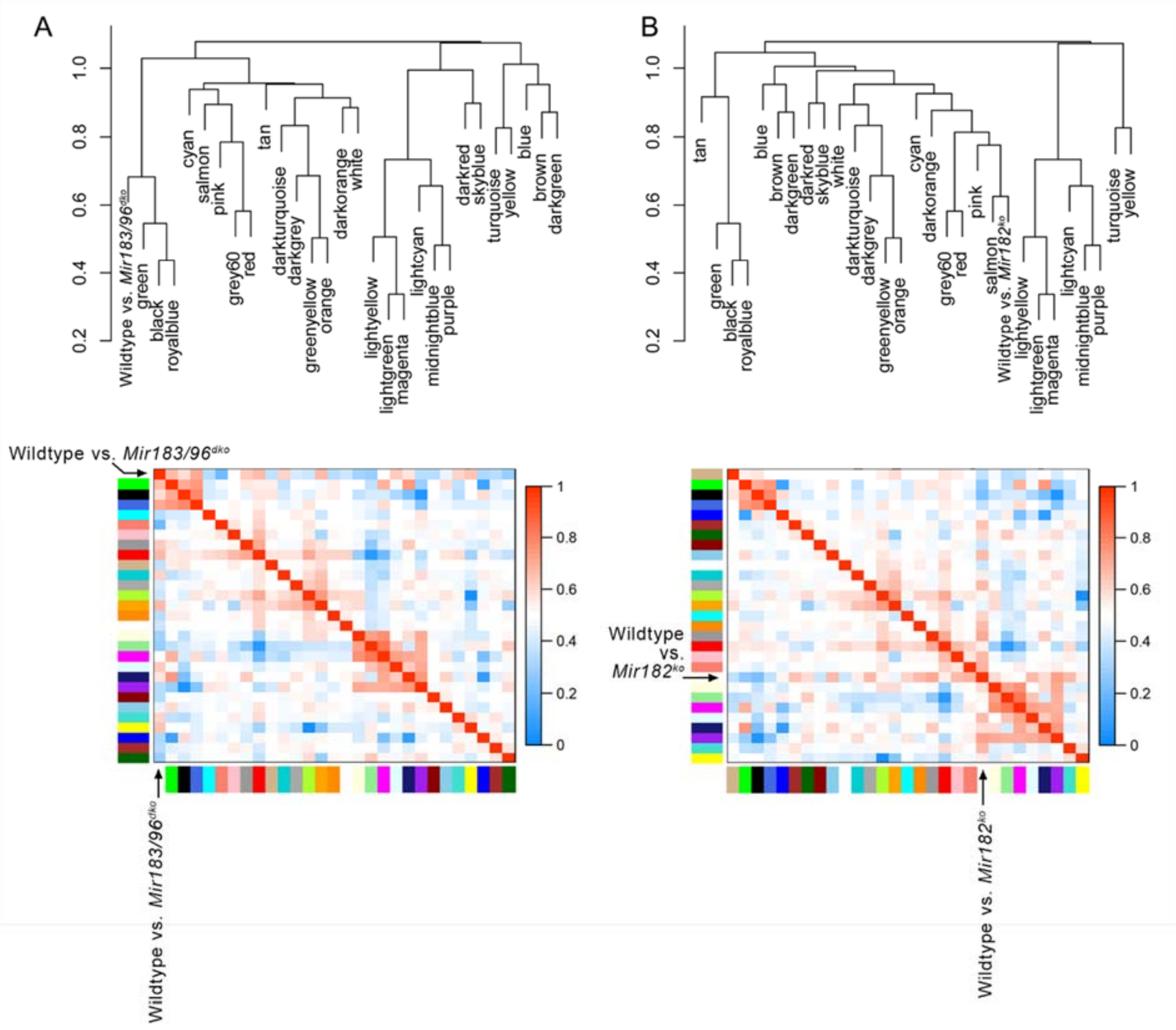

Figure S16. Two visualisations of the relationships between the modules and the two genotype traits we examined (wildtype vs *Mir183/96*^{dko} (A) and wildtype vs *Mir182*^{ko} (B)). The top panel shows a hierarchical clustering dendrogram, and the bottom panel shows a heatmap of eigengene correlations. For the heatmaps, the row and column showing the correlation of the genotype trait with the module eigengenes corresponds to that trait's column in Fig S15.

Disease Models & Mechanisms • Supplementary information

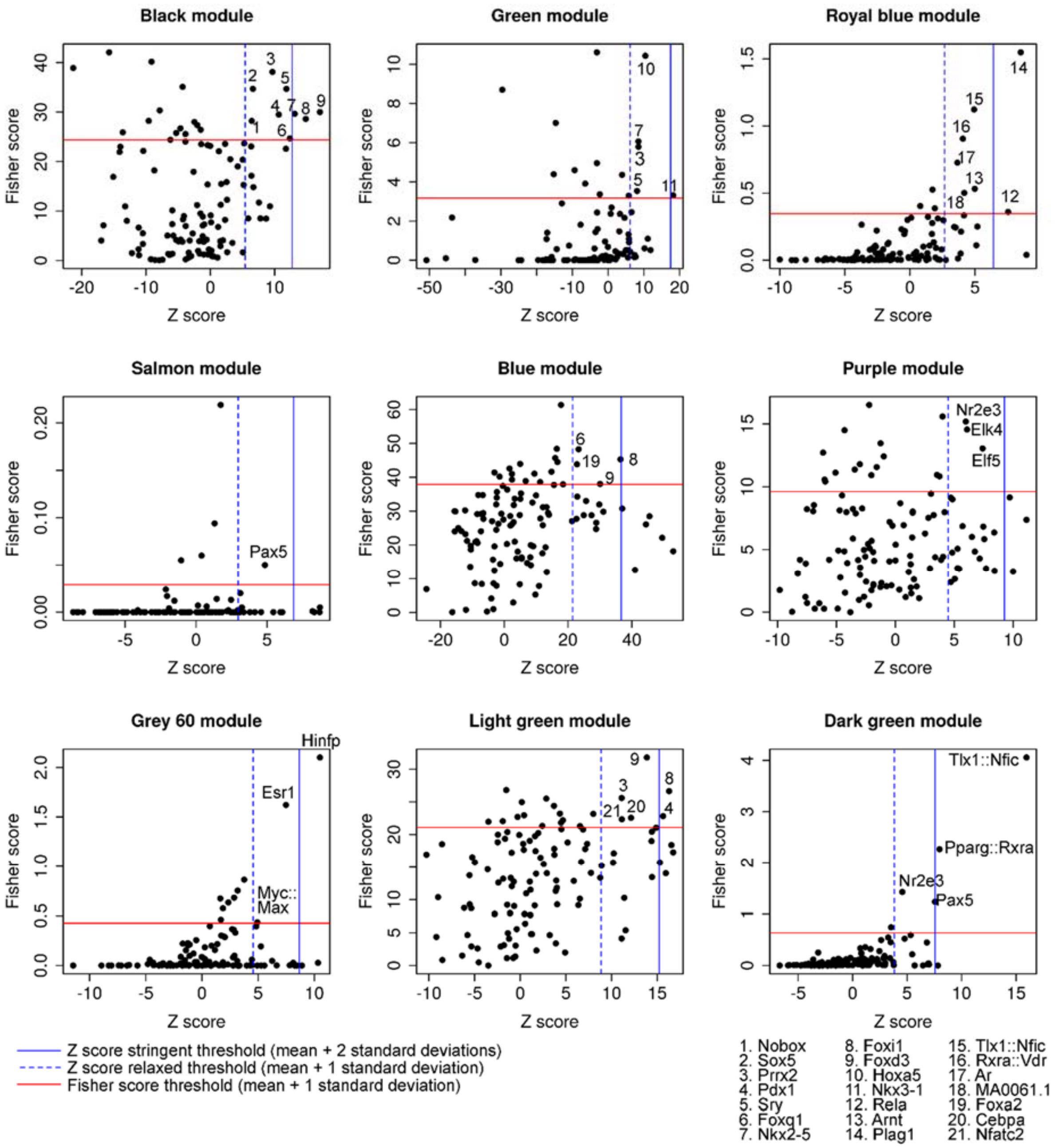

Figure S17. Predicted transcription factors for each module, with Z score plotted along the x axis and Fisher score along the y axis. Chosen thresholds are shown in red (for Fisher score) and blue (Z score; the less stringent threshold is a broken line). Transcription factors scoring above both thresholds are labelled.

Disease Models & Mechanisms • Supplementary information

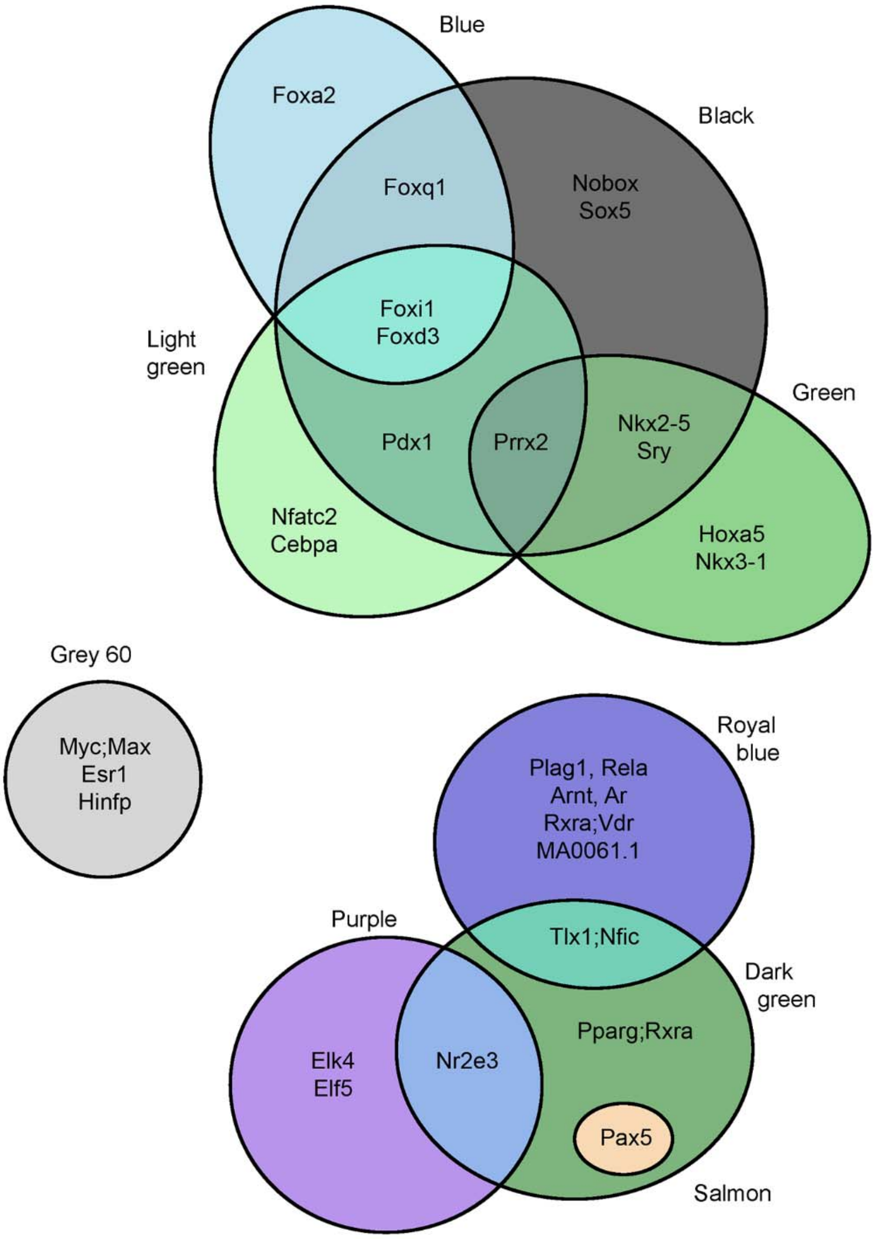

Figure S18. Venn diagram showing which of the oPOSSUM transcription factor predictions are shared between modules.

Disease Models & Mechanisms • Supplementary information

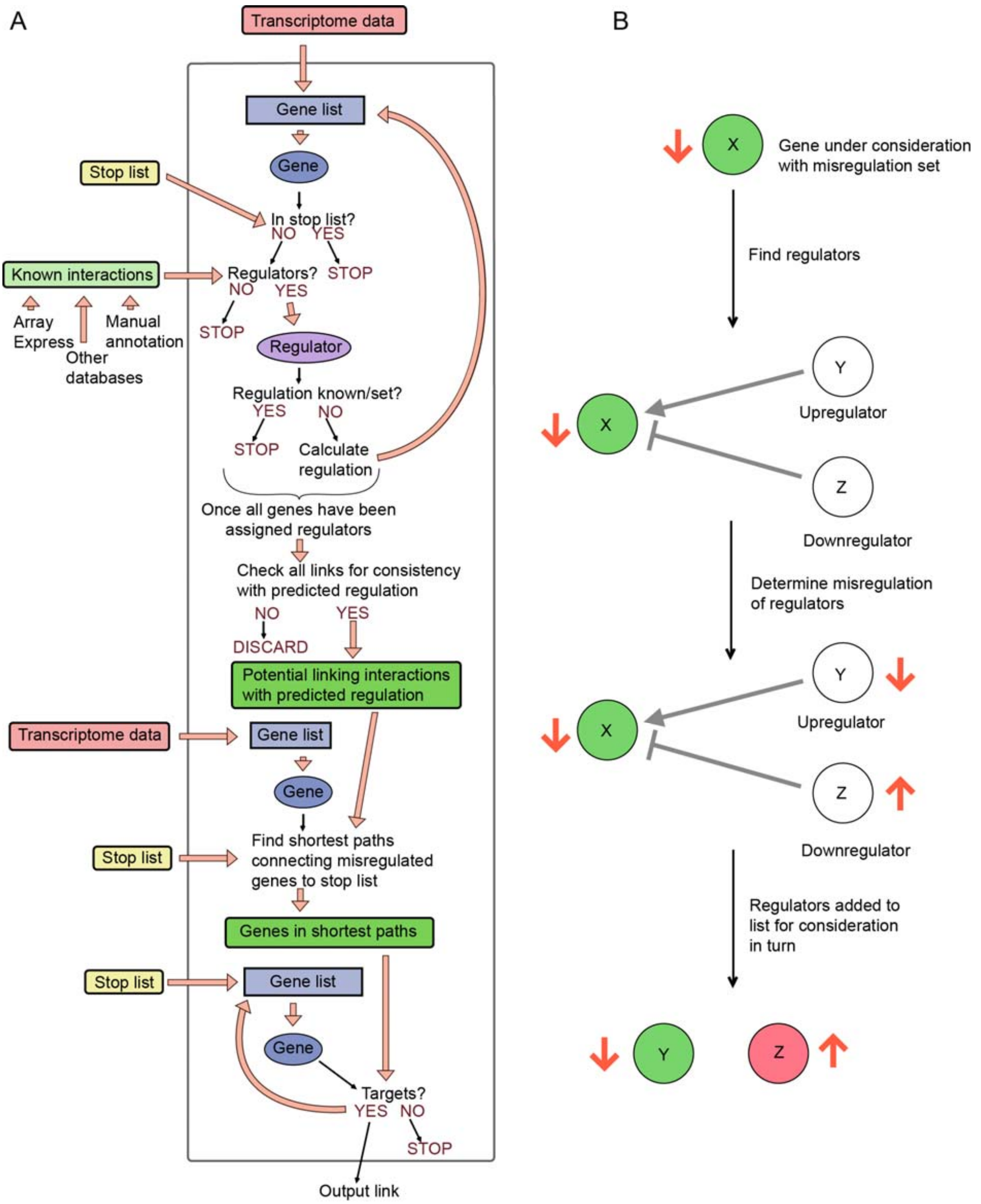

Figure S19. The PoPCoRN script. (A) Schematic showing the steps of network construction carried out by the PoPCoRN script. The stop list is a list of genes at which the algorithm stops its upstream searching. In this case, the stop list consisted of the relevant microRNAs (*Mir182* for the *Mir182*^{ko},

Disease Models & Mechanisms • Supplementary information

and *Mir183* and *Mir96* for the *Mir183/96*^{dko}). (B) Diagram of the determination of direction of regulation of upstream regulators. Red arrows indicate direction of misregulation. Green indicates a downregulated gene, and pink an upregulated gene.

Disease Models & Mechanisms • Supplementary information

Table S1. Matches for the complement of the miR-96 seed region (GTGCCAA) in 3'UTRs from C57BL/6 and C3H/HeJ sequence. Only genes with at least one match to the miR-96 seed region in their 3' UTRs in one or both strains are shown (1733 in total). The number of matches is colour-coded to aid viewing, from no matches (green) to four matches (red). Direct targets of miR-96 used for creating networks are indicated in the fourth column.

Click here to Download Table S1

Table S2. Genes with significant differential splicing predicted by Cuffdiff, Leafcutter and JunctionSeq in *Mir183/96^dko^* homozygotes (A) and *Mir182^ko^* homozygotes (B). * indicates which predictions were tested by sequencing. None of the differential splicing predictions were confirmed.

(A) *Mir183/96^{dko}*

Cuffdiff		Leafcutter		JunctionSeq	
**Gene**	**Significance (adjusted p value)**	**Gene**	**Significance (FDR)**	**Gene**	**Significance (adjusted p value)**
Rars	0.00972143	Rnf157	0.000261808	Wdpcp*	0.000364
Ncor1	0.00972143	Ppp3cb	0.000437474	Dlgap3*	0.000566
Srr	0.00972143	Slc22a15	0.001567787	Tm2d1	0.00114
Npepps	0.00972143	Spag9	0.001780359	Cdc73	0.00158
Slc38a10	0.00972143	Slc8a1	0.001780359	Stard9*	0.00158
Naa35	0.00972143	Pik3c2a	0.002821861	Nav2*	0.00158
Wdr37	0.00972143	Tnc*	0.003622023	Insc	0.00296
Adgrb1	0.00972143	Abi2	0.004435971	Ddx11*	0.00455
Tmbim6	0.00972143	Nsfl1c	0.00585876	Dnah8*	0.00487
Scn8a	0.00972143	Cap1	0.007645588	Zfp618*	0.0091
Acvrl1	0.00972143	Plscr3	0.007783801	Igf2*	0.0124
Rapgef3	0.00972143	Camk2g	0.009450284	Slc44a5*	0.0151
Cfap126	0.00972143	Nfasc	0.009450284	Adamtsl4*	0.024
Tnk2	0.00972143	Zfp280d	0.010903811	Ppp3cb*	0.029
A930003A15Rik,Gm27883	0.00972143	Tmod1	0.018799915	Nrxn2	0.029
Atat1	0.00972143	Skp1a	0.026059743	Rims3	0.0413
Fam98a	0.00972143	Adgrl3	0.026059743	Greb1l*	0.0435
Cabyr	0.00972143	Raver1	0.026059743	Fam96a*	0.0435
Snhg1	0.00972143	Zfp37	0.027564421	Zfp280c	0.0443

Disease Models & Mechanisms • Supplementary information

Snx15	0.00972143	Xpot	0.030579786	Ranbp17*	0.0482
Slc3a2	0.00972143	Rnf180	0.030579786	Onecut2	0.0482
Fam107b	0.00972143	Sgms1	0.030579786	Stx16	0.0482
Scn2a1	0.00972143	Fcrlb	0.030579786	Gpnmb*	0.0482
Fmn1	0.00972143	Selenom	0.030677894		
Smox	0.00972143	Ppip5k2	0.030677894		
Zeb2	0.00972143	Rbms3	0.030898362		
P2rx3	0.00972143	Sgcd	0.031113858		
Ubr1	0.00972143	Efemp1	0.031665004		
Tcfl5	0.00972143	Slc4a1ap	0.033113361		
Rhoc	0.00972143	Mgat5	0.035600128		
Mcoln3	0.00972143	Clec16a	0.036013137		
Cryz	0.00972143	Cobll1	0.036013137		
Sec24b	0.00972143	Kazn	0.036013137		
Sec61b	0.00972143	Ubn2	0.036013137		
Megf6	0.00972143	Snrk	0.036013137		
Mrpl20	0.00972143	Nckap5l	0.037180232		
Tnfrsf4	0.00972143	Mrpl24	0.037180232		
Ptbp3	0.00972143	Ppfia2	0.037990292		
Brca2	0.00972143	Nufip2	0.037990292		
G3bp2	0.00972143	Copa	0.037990292		
Slc15a4	0.00972143	Fbxl20	0.040667577		
5930412G12Rik	0.00972143	Efr3a	0.040667577		
Auts2	0.00972143	Pfdn5	0.040667577		
Il17re	0.00972143	Ablim1	0.040667577		
Sec13	0.00972143	Gab2	0.040667577		
Clpb	0.00972143	Hpf1	0.040667577		
Anpep	0.00972143	Zfp651	0.040667577		
Spats2l	0.00972143	Rpl26	0.043842403		
Apc2	0.00972143	2810428I15Rik	0.043842403		
Smarcc1	0.00972143	Rnps1	0.044056022		
Birc3	0.00972143	Tial1	0.044056022		
Cntn5	0.00972143	Tbata	0.044623796		
Gramd1b	0.00972143	Tlk2	0.044623796		
Agtr2	0.00972143	Cryl1	0.044623796		
Gm27875,Jpx	0.00972143	Mdga1	0.044623796		
Ctps2	0.00972143	Lcor	0.044623796		
Wrn	0.0191018	Slc25a44	0.044623796		
		Phf13	0.044623796		
		Prkcsh	0.044623796		

Disease Models & Mechanisms • Supplementary information

	Cdv3	0.044623796	
	Decr2	0.044950165	
	Mbd5	0.044950165	
	Carm1	0.046056306	
	Calml4	0.046056306	
	Cd200	0.046119016	
	Pdzd2	0.04741293	
	Zfpm2	0.04741293	
	Arfgef2	0.04741293	
	Zfp618	0.04741293	
	Deaf1	0.04741293	
	Appl1	0.047696496	
	Pdgfc	0.047696496	
	Elovl1	0.047696496	
	Prickle2	0.047696496	
	Il17re	0.047696496	
	Rpl10	0.04782345	
	Dtnb	0.049562528	
	Nol4l	0.049716919	

(B) Mir182^{ko}

**Cuffdiff**		**Leafcutter**	
**Gene**	**Significance (adjusted p value)**	**Gene**	**Significance (FDR)**
Tada2a	0.037425	Ube4a*	0.003625717
Tnfaip2	0.037425	Prdm4	0.013483712
Psmd6	0.037425	Irf2bp2	0.013483712
Snhg7	0.037425	Atrx	0.013483712
Ptpn4	0.037425	Tmem184b	0.016348513
Ptpn3	0.037425	Rad17	0.017626834
Nfyc	0.037425	Pbrm1	0.017626834
Rpl6	0.037425	Nrxn1*	0.017626834
Auts2	0.037425	Fubp3	0.017626834
Casc1	0.037425	Adgrl3	0.017626834
Nr3c2	0.037425	Ddx59	0.017790912
Mre11a	0.037425	Arhgef1	0.017790912
Usp2	0.037425	Kctd17	0.029537826
Ryk	0.037425	Kdm1a	0.029865303

Disease Models & Mechanisms • Supplementary information

	Ptprt	0.036976191
	Adamtsl1	0.036976191
	Cbx1	0.037836631
	Ttc3	0.037836631
	Rsrc1	0.037836631
	Pan3	0.037836631
	Sec23ip	0.037836631
	Col4a5	0.037836631
	Slc39a8	0.038458003
	Ncor2	0.040186555
	Trit1	0.040463733
	Tfpi	0.044934868
	Zfp719	0.045815348

Disease Models & Mechanisms • Supplementary information

Table S3. Primers used for genotyping, differential splicing and qPCR.

(A) Primers for genotyping

Allele	Primer F (5'-3')	Primer R (5'-3')
Mir183/96^{dko}	tattgggatgtgatgggaaactctg	tagcagaaggctagaccccaaagac
Mir182^{ko} (1)	gggtacagtgcctttgagagcagt	gggaaacattaagggtcacttccag
Mir182^{ko} (2)	gcttgaggaggttttacactgg	ttcctggtgatcggcagg

(B) Primers for sequencing

Gene	Transcript	Primer F (5'-3')	Primer R (5'-3')
Wdpcp	ENSMUST00000020568	TCTTAGACAGAGGCTCACACC	CATGCGCCCTTCTTCTTGC
Dlgap3	ENSMUST00000106094	ACACCGAGAACAGGAGTCG	CTGGAAGGTGGGCACAGG
Stard9	ENSMUST00000180041	ACATCATCAACAAGCCACGG	TCTCTCTCAATCCACTGGCC
Ddx11	ENSMUST00000163605	TGCCCCTATTACGGAAGCC	CATGTACTGGAGCAACTGGG
Dnah8	ENSMUST00000170651	CAGGAGGGAGGAAGATGACG	GGTCAAAGTATCCACAGCCG
Zfp618	ENSMUST00000030043	CTGGAAGGAAAAGCGCGG	GATCCCGCATTCATAGGACC
Igf2	ENSMUST00000000033	GAGTTCAGAGAGGCCAAACG	TGTTCTGTTCTTCTCCTTGGG
Slc44a5	ENSMUST00000089948	TTAGCTACCTTCCCAGTGCG	AGATCCAAAGGCCAGAGACC
Adamtsl4	ENSMUST00000117782	TAACGCCATGCTCCTCCC	TGGGCTTCTGGATGTCTTGG
Ppp3cb	ENSMUST00000159027	CAGTTTAATTGCTCTCCACATCC	CAGCACGCTTTCACTCTCC
Greb1l	ENSMUST00000048977	TCAAACAGCCCACCAATTCC	CAAAGAGCAGAGGATTGTCGG
Fam69a	ENSMUST00000034945	CGGATCATGGAAGAGAAAGCG	CACTGTTCCACGATTTCCCG
Ranbp17	ENSMUST00000102815	TTGACAATGTACTCCAGGCC	GGTTCTGTTCCACTCCTTCC
Gpnmb	ENSMUST00000031840	TCTGCCCACATTATCAACACC	TCGGAGATGATCGTACAGGC
Tnc	ENSMUST00000107377	GTTACCGCCTCAACTACAGC	TGAGGTTCTGGACAGTCTGG
Ube4a	ENSMUST00000117506	TAGCTGTGAGGTGTCGTCG	AGTCATCTGGGCTTGCTGG
Nrxn1	ENSMUST00000160844	GCACACCTGATGATGGGC	GCTGATTTCCCTGTGTGAAGC

Disease Models & Mechanisms • Supplementary information

(C) qPCR primer/probes

Gene	Primer/probe catalogue number
Hprt	Mm01318747_g1
Ppm1l	Mm00618786_m1
Jag1	Mm01270190_m1
Ccer2	Mm01179046_g1
Grp	Mm00612977_m1
Grk1	Mm01220714_m1
Tmc1	4331348 (manual design using Applied Biosystems software)
Hspa2	Mm00434069_s1
Myo3a	4331348 (manual design using Applied Biosystems software)
St8sia3	4331348 (manual design using Applied Biosystems software)
Tmem173	Mm01158119_g1
Slc52a3	Mm00510191_g1
Kif21b	Mm01285309_g1
Mfsd6	Mm00505561_m1
BC030867	Mm00463529_m1
Eln	Mm00514696_g1
P2rx3	Mm01278228_g1
Ttc21a	Mm01351694_g1
Slc6a11	Mm01190466_m1
Dtna	Mm01135282_m1
Ikzf2	Mm00496108_m1

Disease Models & Mechanisms • Supplementary information

Rest	Mm00803267_m1
Cebpa	Mm01265914_s1
Cdkn1a	Mm00432448_m1
Fos	Mm01302932_g1
Nr3c1	Mm01260500_m1
Foxo3	Mm01185722_m1
Foxo1	Mm00490672_m1
Tgfb1	Mm00441729_g1
Sp1	Mm03053855_g1
Trp53	Mm01337166_mH
Ocm	Mm00712881_g1
Slc26a5	4331348 (manual design using Applied Biosystems software)

(D) microRNA qPCR primer sets

Gene	Primer set catalogue number
hsa-miR-96-5p	YP00204417
mmu-miR-182-5p	YP00205089
hsa-miR-183-5p	YP00206030
hsa-miR-99a-5p	YP00204521

Disease Models & Mechanisms • Supplementary information

Table S4. Modules detected by WGCNA, their correlation with the two genotype traits we examined (wildtype vs *Mir183/96*^{dko} and wildtype vs *Mir182*^{ko}) and the number of genes and differentially expressed genes in each module. The first nine modules were chosen for further analysis.

**Module**	**black**	**green**	**royal blue**	**salmon**	**blue**	**purple**	**grey60**	**light green**	**dark green**	**pink**
Correlation for wildtype vs. *Mir183/96*^{dko} homozygote trait	0.176	0.358	0.418	-0.594	-0.357	-0.304	0.275	-0.428	-0.430	-0.021
p-value for wildtype vs. *Mir183/96*^{dko} homozygote trait	0.410	0.086	0.042	0.002	0.087	0.148	0.193	0.037	0.036	0.924
Correlation for wildtype vs. *Mir182*^{ko} homozygote trait	-0.543	-0.454	-0.270	0.375	0.078	0.457	0.416	0.053	0.181	0.328
p-value for wildtype vs. *Mir182*^{ko} homozygote trait	0.006	0.026	0.203	0.071	0.717	0.025	0.043	0.807	0.398	0.117
Number of genes	858	1624	261	583	3307	695	314	305	240	851
Number of differentially expressed genes (*Mir183/96*^{dko})	0	3	0	0	13	1	1	2	0	0
Number of differentially expressed genes (*Mir182*^{ko})	0	0	0	0	2	0	0	0	0	0
**Module**	**brown**	**cyan**	**dark grey**	**grey**	**dark red**	**dark turquoise**	**green yellow**	**dark orange**	**light cyan**	**light yellow**
Correlation for wildtype vs. *Mir183/96*^{dko} homozygote trait	-0.297	-0.313	-0.194	-0.245	-0.312	0.210	-0.218	-0.305	0.279	-0.368
p-value for wildtype vs. *Mir183/96*^{dko} homozygote trait	0.159	0.136	0.364	0.250	0.138	0.325	0.306	0.147	0.186	0.077
Correlation for wildtype vs. *Mir182*^{ko} homozygote trait	0.295	0.233	0.330	0.438	-0.232	0.081	0.069	0.341	0.325	0.195
p-value for wildtype vs. *Mir182*^{ko} homozygote trait	0.162	0.273	0.115	0.032	0.276	0.707	0.750	0.103	0.121	0.360
Number of genes	2257	575	131	5972	258	152	666	96	354	298
Number of differentially expressed genes (*Mir183/96*^{dko})	4	0	0	1	0	0	0	0	0	0
Number of differentially expressed genes (*Mir182*^{ko})	1	0	0	0	0	0	0	0	0	0
**Module**	**magenta**	**red**	**orange**	**tan**	**sky blue**	**midnight blue**	**turquoise**	**white**	**yellow**	
Correlation for wildtype vs. *Mir183/96*^{dko} homozygote trait	-0.035	0.347	0.343	-0.242	-0.095	0.138	0.239	-0.238	0.301	
p-value for wildtype vs. *Mir183/96*^{dko} homozygote trait	0.872	0.096	0.100	0.254	0.659	0.520	0.261	0.263	0.153	
Correlation for wildtype vs. *Mir182*^{ko} homozygote trait	-0.016	0.084	-0.089	-0.259	0.326	0.177	0.334	-0.237	0.114	
p-value for wildtype vs. *Mir182*^{ko} homozygote trait	0.940	0.697	0.678	0.222	0.120	0.409	0.111	0.265	0.595	
Number of genes	758	1019	104	622	86	483	8793	90	2225	
Number of differentially expressed genes (*Mir183/96*^{dko})	2	5	0	0	0	0	1	0	1	
Number of differentially expressed genes (*Mir182*^{ko})	0	0	0	0	0	0	0	0	0	

Table S5. REACTOME enrichment scores for the nine WGCNA modules tested. If no entries are shown for a module, no pathway terms were returned at all.

Click here to Download Table S5

Table S6. GO enrichment scores for the nine WGCNA modules tested. If no entries are shown for a module, no GO terms were returned at all.

Click here to Download Table S6

Disease Models & Mechanisms • Supplementary information

Table S7. Transcription factors predicted by oPOSSUM to be involved in the nine modules examined.

Gene symbol	Ensembl	Mir96 seed regions	Mir183 seed regions	Mir182 seed regions	Deafness gene	Module
Nkx2-5	ENSMUSG00000015579					black, green
Foxi1	ENSMUSG00000047861				Yes	black, blue, light green
Foxd3	ENSMUSG00000067261					black, blue, light green
Hoxa5	ENSMUSG00000038253	2		1		green
Sox5	ENSMUSG00000041540	3		2		black
Prrx2	ENSMUSG00000039476					black, green, light green
Sry	ENSMUSG00000069036					black, green
Pdx1	ENSMUSG00000029644			1		black, light green
Nobox	ENSMUSG00000029736					black
Foxq1	ENSMUSG00000038415	1		1		black, blue
Nkx3-1	ENSMUSG00000022061	1	1	1		green
Foxa2	ENSMUSG00000037025					blue
Nfatc2	ENSMUSG00000027544					light green
Cebpa	ENSMUSG00000034957	1		1		light green
Plag1	ENSMUSG00000003282			1		royal blue
Rela	ENSMUSG00000024927					royal blue

Disease Models & Mechanisms • Supplementary information

Pparg;Rxra	ENSMUSG00000000440; ENSMUSG00000015846	1 (Rxra)				dark green
Pax5	ENSMUSG00000014030	3	1	2		dark green, salmon
Tlx1;Nfic	ENSMUSG00000025215; ENSMUSG00000055053	1 (Nfic)	1 (Nfic)	1 (Nfic)		dark green, royal blue
Arnt	ENSMUSG00000015522					royal blue
MA0061.1	Profile no longer in JASPAR database					royal blue
Ar	ENSMUSG00000046532	1	1	1		royal blue
Rxra;Vdr	ENSMUSG00000015846; ENSMUSG00000022479	1 (Rxra)				royal blue
Nr2e3	ENSMUSG00000032292	1				purple, dark green
Elk4	ENSMUSG00000026436		1			purple
Elf5	ENSMUSG00000027186					purple
Hinfp	ENSMUSG00000032119	1	1	2		grey 60
Esr1	ENSMUSG00000019768	1	1	1		grey 60
Myc;Max	ENSMUSG00000022346; ENSMUSG00000059436				Yes (Myc)	grey 60

Disease Models & Mechanisms • Supplementary information

Table S8. Nodes with high edge count in the *Mir183/96*^{dko} network.

Name	Deafness genes	microRNA target	Betweenness Centrality	Edge count
Trp53			0.09422711	23
Foxo1		Yes (both)	0.08571448	21
Sp1			0.06173317	18
Stat5a			0.03490919	17
Jun			0.04868463	16
Nfkb1	Yes		0.03928189	16
Hnf4a			0.04198241	15
Fos	Yes		0.04456005	13
Tnf	Yes		0.05881021	12
Tgfb1			0.02574521	12
Mir155			0.01078878	12
E2f2			0.04550675	11
Pkd1	Yes		0.02115304	11
Tcf7			0.02033637	11
Kit	Yes		0.0124918	11
Ezh2			0.03284497	10
Zfp36			0.02575902	10
Mir200a			0.02321144	10
Elk1			0.01517501	10
Cdkn1a		Yes (miR-96)	0.03321781	23
Mir96	Yes		0.04831501	22
Rest	Yes		0.03424156	17
Mir183			0	16
Egr1		Yes (miR-183)	0.0752374	15
Stat5b			0.00432024	14
Nr3c1		Yes (miR-96)	0.04511961	13
Bcl6			0.03888551	13
Foxo3	Yes	Yes (miR-96)	0.02891802	13
Mir141			0.04075138	12
Smad4		Yes (both)	0.05817905	11
Mir145a			0.02869746	11
Mir182			0.02760045	11
Cebpa			0.00923269	11
Zeb1	Yes	Yes (both)	0.03369301	10

Disease Models & Mechanisms • Supplementary information

Table S9. Data used as a basis for building the regulatory networks.

Database	Format example	Number of Unique Entries	Reference
ArrayExpress and the Gene Expression Atlas	If a null allele of gene A results in a decrease in expression of gene B, gene A upregulates gene B	87424	(Athar et al., 2019; Petryszak et al., 2016)
ORegAnno	If there is a binding site in gene B for gene A, and the outcome of the interaction is NEGATIVE, gene A downregulates gene B	1044	(Lesurf et al., 2016)
miRTarBase	If microRNA X has target gene A, X downregulates A	6199	(Chou et al., 2018)
TransmiR	If gene A represses microRNA X, A downregulates X	1596	(Tong et al., 2019)
TRRUST	If the relationship between gene A and gene B is activation, gene A upregulates gene B	8265	(Han et al., 2018)
n/a	Various	519	(Hertzano et al., 2007; Lewis et al., 2016)
n/a	If a null allele of microRNA X results in an increase in expression of gene A, and gene A has seed region matches for X, microRNA X downregulates gene A	18	Current study

Table S10. Data used for checking the PoPCoRN predictions.

Upstream regulator	Perturbation	Tissue	Age	Intermediate gene	Paper confirmation	PoPCoRN prediction	Number of input genes	Reference
Tbx1	Reduced function due to missense mutation	Inner ear	E16.5	*Esrrb*	Downregulated	Downregulated	39	(Tian and Johnson, 2020)
Stk11	Knockout	Chondrocytes	P30	*Runx2*	Upregulated	Upregulated	962	(Liang et al., 2018)
Stk11	Knockout	Chondrocytes	P30	*Nfatc1*	Upregulated	Downregulated	962	(Liang et al., 2018)
Stk11	Knockout	Chondrocytes	P30	*Mir134*	Upregulated	Upregulated	962	(Liang et al., 2018)
Yy1	Knockout	Haematopoietic stem cells	-	*Kit*	Downregulated	Upregulated	55	(Lu et al., 2018)
Sox2	Induced knockout	Acinar cells of salivary gland	E16.5	*Sox10*	Downregulated	Upregulated	7	(Emmerson et al., 2017)
Trp53	Knockout	Cultured osteoblasts	-	*Runx2*	Upregulated	Upregulated	3	(Artigas et al., 2017)
Trp53	Knockout	Cultured osteoblasts	-	*Sp7*	Upregulated	Upregulated	3	(Artigas et al., 2017)

Disease Models & Mechanisms • Supplementary information

Yy1	Inhibition by siRNA	HUVECs	-	*Rbpj*	Upregulated	Upregulated	15	(Zhang et al., 2020a)
Brca1	Inhibition by siRNA	HUVECs	-	*Cdkn1a*	Downregulated	Downregulated	3	(Zeng et al., 2020)
Hif1a	Induction by hypoxia	HepG2/HeLa cells	-	*Bclaf1*	Upregulated	No prediction	4	(Shao et al., 2020)
Gata4	Inhibition by shRNA	Dental pulp stem cells	-	*Fbp1*	Upregulated	No prediction	9	(Zhang et al., 2020b)
Arntl	Knockout	Mandibular condyle	P28	*Ihh*	Downregulated	No prediction	7	(Yu et al., 2020)
Arntl	Knockout	Mandibular condyle	P28	*Ptch1*	Downregulated	No prediction	7	(Yu et al., 2020)

Data S1. Data underlying the graphs and charts presented in this study.

Click here to Download Data S1

Disease Models & Mechanisms • Supplementary information

Supplementary References

Artigas, N., Gamez, B., Cubillos-Rojas, M., Sanchez-De Diego, C., Valer, J. A., Pons, G., Rosa, J. L. & Ventura, F. (2017). p53 inhibits SP7/Osterix activity in the transcriptional program of osteoblast differentiation. *Cell Death Differ,* **24,** 2022-2031.

Emmerson, E., May, A. J., Nathan, S., Cruz-Pacheco, N., Lizama, C. O., Maliskova, L., Zovein, A. C., Shen, Y., Muench, M. O. & Knox, S. M. (2017). SOX2 regulates acinar cell development in the salivary gland. *Elife,* **6**.

Liang, S., Zhang, J. M., Lv, Z. T., Cheng, P., Zhu, W. T. & Chen, A. M. (2018). Identification of Skt11-regulated genes in chondrocytes by integrated bioinformatics analysis. *Gene,* **677,** 340-348.

Lu, Z., Hong, C. C., Kong, G., Assumpcao, A., Ong, I. M., Bresnick, E. H., Zhang, J. & Pan, X. (2018). Polycomb Group Protein YY1 Is an Essential Regulator of Hematopoietic Stem Cell Quiescence. *Cell Rep,* **22,** 1545-1559.

Shao, A., Lang, Y., Wang, M., Qin, C., Kuang, Y., Mei, Y., Lin, D., Zhang, S. & Tang, J. (2020). Bclaf1 is a direct target of HIF-1 and critically regulates the stability of HIF-1alpha under hypoxia. *Oncogene*.

Tian, C. & Johnson, K. R. (2020). TBX1 is required for normal stria vascularis and semicircular canal development. *Dev Biol,* **457,** 91-103.

Yu, S., Tang, Q., Xie, M., Zhou, X., Long, Y., Xie, Y., Guo, F. & Chen, L. (2020). Circadian BMAL1 regulates mandibular condyle development by hedgehog pathway. *Cell Prolif,* **53,** e12727.

Zeng, Z. M., Du, H. Y., Xiong, L., Zeng, X. L., Zhang, P., Cai, J., Huang, L. & Liu, A. W. (2020). BRCA1 protects cardiac microvascular endothelial cells against irradiation by regulating p21-mediated cell cycle arrest. *Life Sci,* **244,** 117342.

Zhang, S., Kim, J. Y., Xu, S., Liu, H., Yin, M., Koroleva, M., Guo, J., Pei, X. & Jin, Z. G. (2020a). Endothelial-specific YY1 governs sprouting angiogenesis through directly interacting with RBPJ. *Proc Natl Acad Sci U S A,* **117,** 4792-4801.

Zhang, Y., Fang, M., Yang, Z., Qin, W., Guo, S., Ma, J. & Chen, W. (2020b). GATA Binding Protein 4 Regulates Tooth Root Dentin Development via FBP1. *Int J Biol Sci,* **16,** 181-193.
